# Search for Fibrous Aggregates Potentially Useful in Regenerative Medicine Formed under Physiological Conditions by Self-Assembling Short Peptides Containing Two Identical Aromatic Amino Acid Residues

**DOI:** 10.3390/molecules23030568

**Published:** 2018-03-02

**Authors:** Justyna Fraczyk, Wojciech Lipinski, Agata Chaberska, Joanna Wasko, Kamil Rozniakowski, Zbigniew J. Kaminski, Maciej Bogun, Zbigniew Draczynski, Elzbieta Menaszek, Ewa Stodolak-Zych, Marta Kaminska, Beata Kolesinska

**Affiliations:** 1 Institute of Organic Chemistry, Lodz University of Technology, Zeromskiego 116, 90-924 Lodz, Poland; justyna.fraczyk@p.lodz.pl (J.F.); wojtek.p.lipinski@gmail.com (W.L.); agata.chaberska@p.lodz.pl (A.C.); joanna.wasko@p.lodz.pl (J.W.); kamilrozniakowski@wp.pl (K.R.); zbigniew.kaminski@p.lodz.pl (Z.J.K.); 2 Department of Material and Commodity Sciences and Textile Metrology, Lodz University of Technology, Zeromskiego 116, 90-924 Lodz, Poland; maciej.bogun@p.lodz.pl (M.B.); zbigniew.draczynski@p.lodz.pl (Z.D.); 3 Department of Cytology, CMUJ—Jagiellonian University Medical College, Swietej Anny 12, 31-008 Krakow, Poland; elzbieta.menaszek@uj.edu.pl; 4 Department of Biomaterials, AGH—University of Science and Technology, A. Mickiewicz 30, 30-059 Krakow, Poland; stodolak@agh.edu.pl; 5 Division of Biophysics, Institute of Materials Science and Engineering, Lodz University of Technology, Stefanowskiego 1/15, 90-924 Lodz, Poland; marta.kaminska@p.lodz.pl

**Keywords:** peptides self-assembling, aromatic peptides, enantiomeric aggregates, cytocompatible scaffold, regenerative medicine

## Abstract

This study investigates the propensity of short peptides to self-organize and the influence of aggregates on cell cultures. The dipeptides were derived from both enantiomers of identical aromatic amino acids and tripeptides were prepared from two identical aromatic amino acids with one cysteine or methionine residue in the C-terminal, N-terminal, or central position. The formation or absence of fibrous structures under physiological conditions was established using Congo Red and Thioflavine T assays as well as by microscopic examination using normal and polarized light. The in vitro stability of the aggregates in buffered saline solution was assessed over 30 days. Materials with potential for use in regenerative medicine were selected based on the cytotoxicity of the peptides to the endothelial cell line EA.hy 926 and the wettability of the surfaces of the films, as well as using scanning electron microscopy. The criteria were fulfilled by H-*d*Phe*d*Phe-OH, H-*d*Cys*d*Phe*d*Phe-OH, H-CysTyrTyr-OH, H-*d*Phe*d*Phe*d*Cys-OH, H-TyrTyrMet-OH, and H–TyrMetTyr–OH. Our preliminary results suggest that the morphology and cell viability of L919 fibroblast cells do not depend on the stereochemistry of the self-organizing peptides.

## 1. Introduction

Self-assembled peptide nanomaterials [1,2,3,4] are becoming increasingly important in medicine. They combine the attributes of nanomaterials with properties characteristic of biomolecules, due to the biodegradability, biocompatibility, and biomimicry of peptides. Self-assembled peptide nanomaterials have many applications, such as for drug delivery [5,6,7], in nanobiotechnology [8], and in regenerative medicine [9,10]. Several nanomaterials formed by self-assembled peptides have been identified as promising scaffolds for tissue engineering [11,12]. The self-assembly of peptides is triggered mainly by hydrophobic interactions. The structures are stabilized by electrostatic forces, although other interactions such as van der Waals interactions, π-π interactions and hydrogen bonds may also be involved. Combined, these weak forces can modify the physicochemical properties of the parent molecules dramatically, creating new materials with novel characteristics.

Amphiphilic peptides [13,14,15], usually composed of repeating units of positively charged amino acid residues and negatively charged amino acid residues separated by hydrophobic neutral amino acid residues, have been widely used in regenerative medicine. The presence of alternative hydrophilic and hydrophobic fragments strongly promotes the process of self-assembly, leading to highly ordered supramolecules. These supramolecules remain stable across wide variations in temperature and pH, and despite the presence of denaturing agents. The best-characterized examples in this class are: RADA16-I (Ac-RADARADARADARADA-NH_2_) and RADA16-II (Ac-RARADADARARADADA-NH_2_). A mixture of peptides, RADA16 RADA16-I and-II, is commercially available under the trade name PuraMatrixTM. These peptides have been used as scaffolds for the regeneration of bones, blood vessels, cartilage, nerves, and nervous tissue [16,17,18,19]. Another peptide from the group of amphiphilic peptides with an ionic structure is AcN-KLDLKLDLKLDL-CNH_2_, which has been used in the regeneration of intervertebral discs [20].

Peptides which aggregate to form amyloid fibrils or amyloid-type fibrils are also applied in regenerative medicine. A characteristic feature of this group of peptides is the formation of fibrous nanostructures [21,22] containing a β-sheet motif. The formation of fibrous nanostructures has been linked to amyloid deposits and amyloidosis (associated with diseases including Alzheimer’s, Parkinson’s, and diabetes). A common motif composed of phenylalanine residues (FF) has been identified in naturally occurring β-amyloid deposits [23]. It is characterized by relatively weak interactions between aromatic rings upholding β-sheet structures [24,25]. This finding triggered intensive research into possible applications for short-aromatic (hydrophobic) peptides in medicine [26,27,28,29,30,31,32].

Given the unusual properties of the FF peptide, confirmed by extensive use, the present study set out to answer the following questions: (i) is FF the only aromatic dipeptide with the propensity to form ordered structures through self-assembly? (ii) is it possible to obtain highly ordered peptide materials composed of other aromatic amino acids with a moderately hydrophilic cysteine residue in the C*-*terminal position? (iii) is the presence of cysteine in peptides containing aromatic amino acids a prerequisite for the formation of ordered structures? (iv) what effect does the presence of the moderately hydrophobic methionine residue in the peptide have? (v) is it possible to use aromatic peptides to obtain useful materials for scaffolds? 

## 2. Results and Discussion

Gazit and co-authors report that even dipeptides composed of phenylalanine or phenylglycine are capable of forming nanotubes and nanospheres, respectively [33,34,35,36,37,38]. In our study, we focused on the possible applications of short peptides containing hydrophobic aromatic amino acid residues. In the preliminary stage of our study, we investigated the morphology of structures formed by aggregation under physiological conditions (37 °C, pH 7.2). It was assumed that H–PhePhe–OH (**1**) would also form highly ordered structures under these conditions and that this would constitute an amyloid-like peptide model. The effects of peptide configuration on self-organization were also investigated, using H–*d*Phe*d*Phe–OH (**1-*ent***). To answer the question of whether only dipeptides composed of phenylalanine and phenylglycine aggregated to form ordered structures, H–TrpTrp–OH (**2**) and H–TyrTyr–OH (**3**) were also tested. All the peptides were synthesized according to SPPS protocols, using 4-(4,6-dimethoxy-1,3,5-triazin-2-yl)-4-methylmorpholinium toluene-4-sulfonate (DMT/NMM/TosO^−^) as a coupling reagent [39]. The purity of the crude isolated peptides ranged from 96.5 to 99.95% (see Supporting Materials).

**Figure 1:**
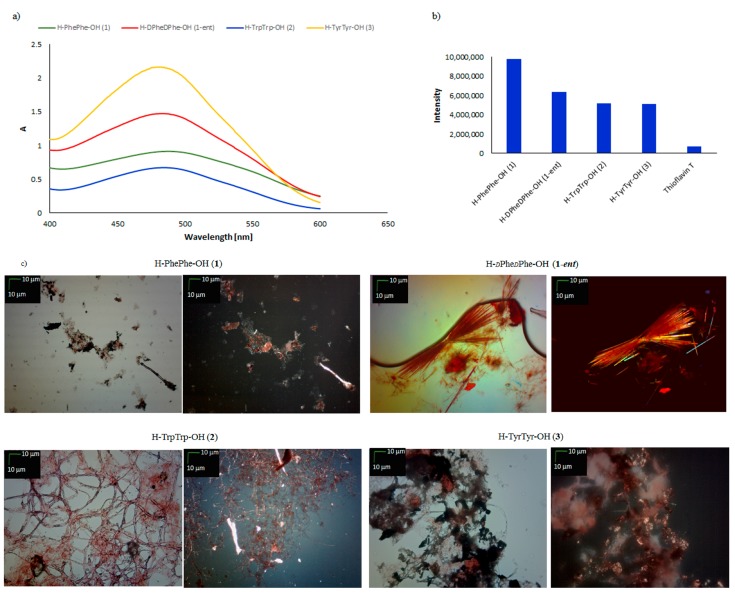
(**a**) UV-Vis spectra of Congo Red (CR) in the presence of H–PhePhe–OH (**1**), H–*d*Phe*d*Phe–OH (**1-*ent***), H–TrpTrp–OH (**2**) and H–TyrTyr–OH (**3**). Spectra were obtained on the fourth day of incubation; (**b**) fluorescence intensity of ThT in the presence of peptides **1**, **1-*ent***, **2**, and **3**. Spectra were obtained on the fourth day of incubation; (**c**) pictures of peptides **1**, **1-*ent***, **2**, and **3**, taken without a polarized filter (left side) and with a polarized filter (right side) (10×) (Scale bars, 10 μm). Samples for microscopic examination were taken on the fourth day of incubation.

Susceptibility to self-organization was tested using three standard and independent methods [40,41,42,43,44,45,46]: the Congo Red (CR) assay, the Thioflavin T (ThT) assay, and microscopic examination. In the CR assay, characteristic shifts of the absorbance maximum were observed in the UV-Vis spectra for peptide **1** and its enantiomer **1-*ent***, from 489 nm to 542.5 nm (Figure 1a). The characteristic absorbance fell during incubation (see Supporting Materials). Similar results were obtained for the fluorescence ThT assay. In both cases, the expected increase in fluorescence intensity was observed. For peptide **1**, the intensity of fluorescence was around 10 × 10^6^, and that of its enantiomer (**1-*ent***) 6 × 10^6^. The fluorescence intensity of Thioflavin T incubated under the same conditions was 7 × 10^5^. Based on a CR assay, peptide **2** was classified as amyloid-like, because of the characteristic shift of its maximum absorbance. The amyloid-like properties of peptide **2** were also confirmed by a ThT test (intensity of fluorescence = 5.2 × 10^6^). Peptide **3** showed a significant increase in fluorescence intensity during incubation (see Supporting Materials), from 2 × 10^6^ in the early stages to over 5.5 × 10^6^ on the fourth day of incubation. However, the CR assay results were ambiguous, because the rapid increase in absorbance exceeded the limits of measurement, causing problems for data interpretation (Figure 1a).

However, microscopic examination under normal and polarized light revealed the presence of typical fibril structures in the dipeptides H–PhePhe–OH (**1**), H–*d*Phe*d*Phe–OH (**1-*ent***) and H–TrpTrp–OH (**2**) (Figure 1c). The results of microscopic studies for dipeptide H–TyrTyr–OH (**3**) were ambiguous and difficult to interpret.

**Figure 2:**
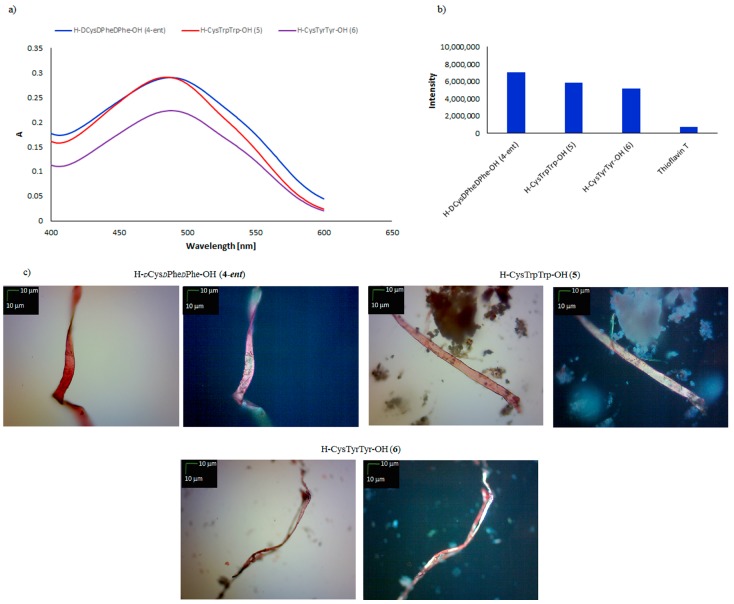
(**a**) UV-Vis spectra of CR in the presence of H–*d*Cys*d*Phe*d*Phe–OH (**4-*ent***), H–CysTrpTrp–OH (**5**), and H–CysTyrTyr–OH (**6**). Spectra were obtained on the fourth day of incubation; (**b**) fluorescence intensity of ThT in the presence of peptides **4-*ent***, **5**, and **6**. Spectra were obtained on the fourth day of incubation; (**c**) pictures of peptides **4-*ent***, **5**, and **6**, taken without a polarized filter (**left side**) and with a polarized filter (**right side**) (10×) (Scale bars, 10 μm). Samples for microscopic examination were taken on the fourth day of incubation.

From the literature, it is known that the introduction of cysteine residues in the N-terminal position changes the morphology of the aggregates, from nanotubes for H–PhePhe–OH to nanospheres for H–CysPhePhe–OH [38]. Therefore, in order to determine the influence of the configuration and structure of the side chains in aromatic amino acids, H–*d*Cys*d*Phe*d*Phe–OH (**4-*ent***), H–CysTrpTrp–OH (**5**) and H–CysTyrTyr–OH (**6**) were obtained and tested. Although it had been reported that the enantiomer deposited from *l*-amino acids forms nanospheres as a result of self-assembly [38], it was found that H–*d*Cys*d*Phe*d*Phe–OH (**4-*ent***) aggregated under physiological conditions to amyloid-like structures. Microscopic examinations (Figure 2c), both with a polarized filter (right panel) and under normal light (left panel), revealed fibrous structures with a characteristic color. The **4-*ent*** peptide also fulfilled the criteria according to the CR and ThT aggregation assays. In the CR test, shifts were observed of the absorbance maximum from 489 nm to 542.5 nm (Figure 2a), whilst in ThT assay the fluorescence intensity was measured at 7 × 10^6^ (Figure 2b).

**Figure 3:**
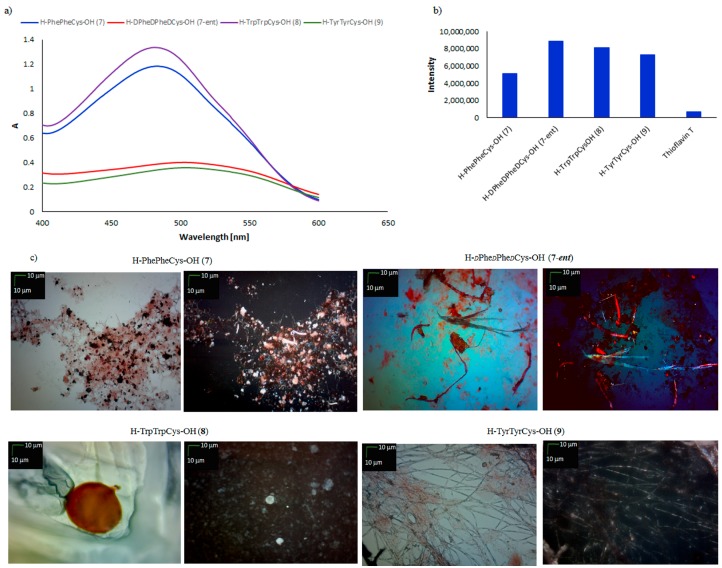
(**a**) UV-Vis spectra of CR in the presence of H–PhePheCys–OH (**7**), H–*d*Phe*d*Phe*d*Cys–OH (**7-*ent***), H–TrpTrpCys–OH (**8**), and H–TyrTyrCys–OH (**9**). Spectra were obtained on the fourth day of incubation; (**b**) fluorescence intensity of ThT in the presence of peptides **7**, **7-*ent***, **8**, and **9**. Spectra were obtained on the fourth day of incubation; (**c**) pictures of peptides **7**, **7-*ent***, **8**, and **9**, taken without a polarized filter (left side) and with a polarized filter (right side) (10×) (Scale bars, 10 μm). Samples for microscopic examination were taken on the fourth day of incubation.

Similar results were obtained for peptides **5** and **6**. According to measurements, the structures containing a cysteine residue in the N-terminal position can be categorized as amyloid-like, because all three tested peptides showed characteristic amyloid properties (positive results in CR, ThT, and microscopic assays) (Figure 2). The impact of the C-terminal position of the cysteine residue on the predisposition for self-assembly was also investigated, in the cases of the tripeptides H–PhePheCys–OH (**7**), H–*d*Phe*d*Phe*d*Cys–OH (**7-*ent***), H–TrpTrpCys–OH (**8**), and H–TyrTyrCys–OH (**9**) (Figure 3). 

Spectroscopic examination of the peptides containing cysteine residue in the C-terminal position showed, in all cases, shifting of the absorbance maximum on successive days during incubation (CR assay) [47,48,49,50]. However, characteristic lowering of the absorbance maximum over time was observed only in the cases of H–*d*Phe*d*Phe*d*Cys–OH (**7-*ent***) and H–TyrTyrCys–OH (**9**). Fluorescence measurements (ThT assay) revealed a characteristic increase in the fluorescence intensity (Figure 3b) of all the peptides; but the fluorescence intensity of peptide **7** was lower (5 × 10^6^) than those of peptides **7-*ent*** and **8** (fluorescence intensity <8 × 10^6^). Microscopic observation of tripeptides stained with CR showed that peptides **7**, **7-*ent***, **8**, and **9** formed different types of ordered structure (Figure 3b). For enantiomeric peptides **7** and **7-*ent***, the typical fibers were either amyloid-like structures (H–*d*Phe*d*Phe*d*Cys–OH, **7-*ent***) or clusters of aggregates with characteristic staining, which was visible both with and without a polarized filter in the case of H–PhePheCys–OH (**7**). Peptide H–TyrTyrCys–OH (**9**) fulfilled all criteria of amyloid-like structures (positive results in all independent assays). However, microscopic examination of peptide H–TrpTrpCys–OH (**8**) revealed the surprising presence of spherical structures. This result confirms that incorporating additional cysteine residues in the C-terminal position changes the morphology of the aggregate (for H–TrpTrp–OH (**2**) and H–CysTyrTyr–OH (**6**) fibril structures were observed, compare Figure 1 and Figure 2). 

**Figure 4:**
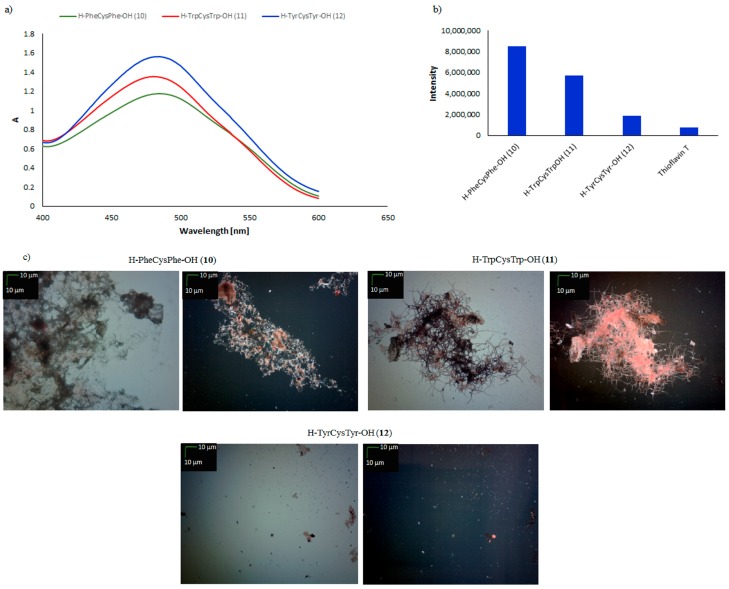
(**a**) UV-Vis spectra of CR in the presence of H–PheCysPhe–OH (**10**), H–TrpCysTrp–OH (**11**), and H–TyrCysTyr–OH (**12**). Spectra were obtained on the fourth day of incubation; (**b**) fluorescence intensity of ThT in the presence of peptides **10**, **11**, and **12**. Spectra were obtained on the fourth day of incubation; (**c**) pictures of peptides **10**, **11**, and **12**, taken without a polarized filter (left side) and with a polarized filter (right side) (10×) (Scale bars, 10 μm). Samples for microscopic examination were taken on the fourth day of incubation.

Analysis of tripeptides containing cysteine residue in the central position (H–PheCysPhe–OH (**10**), H–TrpCysTrp–OH (**11**), H–TyrCysTyr–OH (**12**)) clearly indicated that the position of the cysteine residue is very important from the point of view of susceptibility to aggregation and the formation of ordered structures (Figure 4). For peptides **10** and **11**, a characteristic new maximum absorbance was observed at 542.5 nm in a CR test, which could indicate amyloidogenic properties, confirmed additionally by the ThT test (fluorescence intensities 8.4 × 10^6^ and 5.7 × 10^6^, respectively) and by microscopic examination, which revealed the presence of clusters of aggregates formed from fibrous structures (Figure 4c).

**Figure 5:**
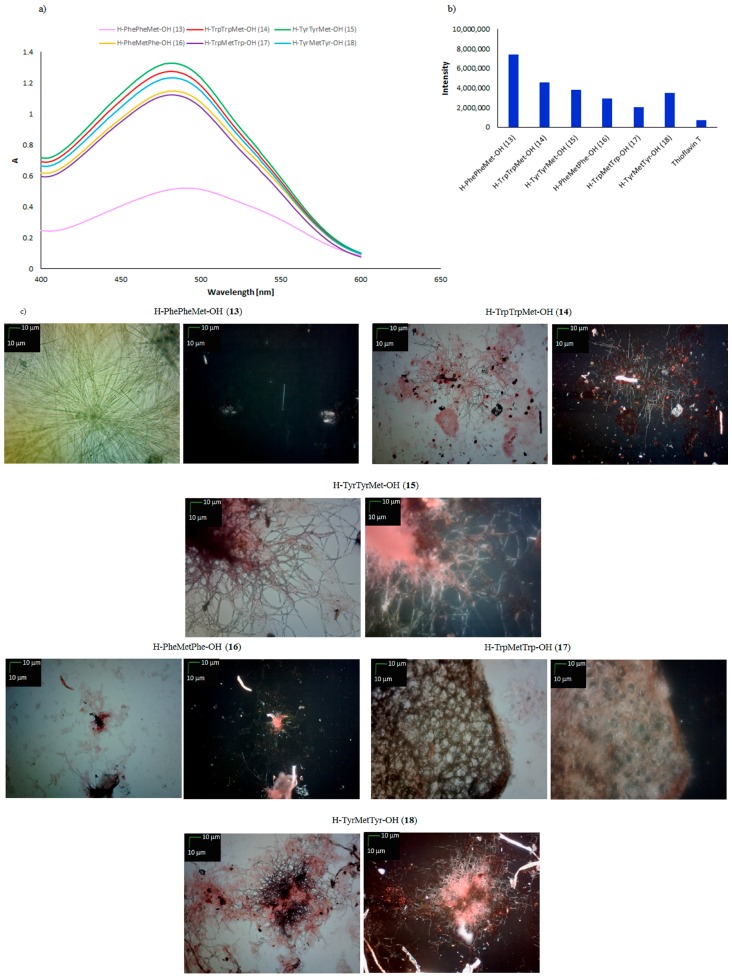
(**a**) UV-Vis spectra of CR in the presence of H–PhePheMet–OH (**13**), H–TrpTrpMet–OH (14), H–TyrTyrMet–OH (**15**), H–PheMetPhe–OH (**16**), H–TrpMetTrp–OH (**17**), and H–TyrMetTyr–OH (**18**). Spectra were obtained on the fourth day of incubation; (**b**) fluorescence intensity of ThT in the presence of peptides **13**–**18**. Spectra were obtained on the fourth day of incubation; (**c**) pictures of peptides **13**–**18**, taken without a polarized filter (left side) and with a polarized filter (right side) (10×) (Scale bars, 10 μm). Samples for microscopic examination were taken on the fourth day of incubation.

For peptide **12**, the results of CR and ThT tests were ambiguous. No increase in fluorescence was observed. Moreover, microscopic examination of the morphology of the deposits suggested an absence of fibrinous structures but the presence of spheroid material. Identical tests were performed using peptides **13**–**18**, containing a methionine residue instead of a cysteine residue in the C-terminal and central positions. It was expected that these tests would confirm whether the susceptibility of short aromatic peptides **4**–**12** to aggregate is related to the presence of cysteine residue. In the pool of tested peptides, only H–PhePheMet–OH (**13**) can be included in the group of amyloid-type peptides. A CR test unambiguously indicated the formation of fibrous structures (Figure 5a). A characteristic decrease in absorbance and a shift of the absorption maximum to 540 nm was visible in the UV-Vis spectrum. This result was also confirmed by a ThT test, in which a characteristic increase in fluorescence intensity up to 8 × 10^6^ was observed (Figure 5b). 

Microscopic examination both with and without a polarizing filter indicated the formation of fibrous structures by H–PhePheMet–OH (**13**) (Figure 5c). For the other peptides in this group (**14**–**18**), the results of CR assays were ambiguous. A slight bulge on the UV-Vis spectra was observed at wavelengths above 500 nm, which allows them to be classified as amyloidogenic peptides. However, no characteristic decreases in absorbance were observed (Figure 5a). A small increase in fluorescence intensity was noted for peptides **14**–**18** (from 4.5 × 10^6^ to 3 × 10^6^), whereas for H–TrpMetTrp–OH (**17**), the fluorescence intensity was only 2 × 10^6^ (Figure 5b). Microscopic examination of peptide **17** revealed the formation of amorphous structures. The remaining peptides containing a methionine residue in the peptide chain were observed to form clusters of aggregates built from fibrous and amorphous structures (Figure 5c). Table 1 presents a summary of the susceptibility to aggregation of short aromatic peptides containing a cysteine or methionine residue. 

We next investigated the predisposition of the ordered structures formed as a result of the self-organization of short aromatic peptides under physiological conditions to be a biocompatible material. Peptides H–*d*Phe*d*Phe–OH (**1-*ent***), H–TrpTrp–OH (**2**), H–*d*Cys*d*Phe*d*Phe–OH (**4-*ent***), H–CysTrpTrp–OH (**5**), H–CysTyrTyr–OH (**6**), H–*d*Phe*d*Phe*d*Cys–OH (**7-*ent***), H–TyrTyrCys–OH (**9**), H–TrpCysTrp–OH (**11**), H–PhePheMet–OH (**13**), H–TrpTrpMet–OH (**14**), H–TyrTyrMet–OH (**15**), and H–TyrMetTyr–OH (**18**) were tested to determine their physicochemical properties and verify whether their fibrous structures fulfilled the criteria of cytocompatibility. Peptides **1-*ent***, **2**, **4-*ent***, **5**, **6**, **7-*ent***, **9**, **11**, and **13** met the criteria for categorization as amyloid-like peptides, i.e., positive results in CR and ThT assays, with microscopic examination confirming the formation of fibrous structures. For peptides **14**, **15**, and **18** the results of CR and ThT tests were ambiguous. However, microscopic examination revealed the presence of clusters of predominantly fibrous structures (Figure 5c).

**Figure 6:**
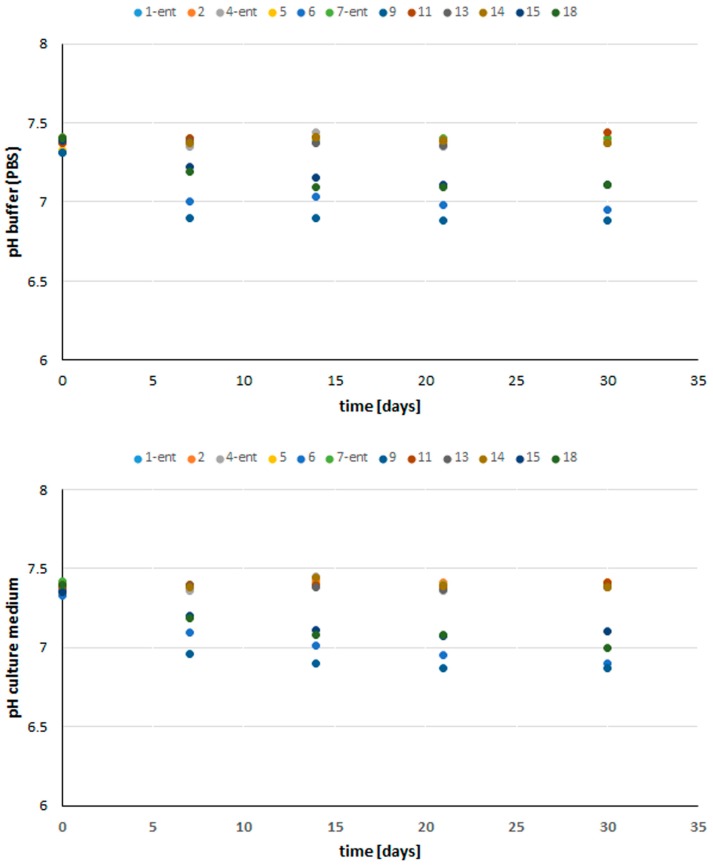
Stability of selected peptides in PBS buffer and culture medium.

The in vitro stability of the selected peptides in buffered saline solution was observed and tested using pH measurements. All the tested peptides were found to be stable (Figure 6). For peptides **8**–**11**, the changes observed in the pH were negligible. In the cases of peptides **7** and **12**, small changes were noted in the hydrogen ion concentration over the course of the experiment. The pH increased slightly, to 8, only in the case of peptide **12**. This small change was probably due to the nature of the side chain structures of the amino acid residues incorporated in the peptide chain. Tyrosine, with an acidic phenolic group, is characterized by a pK_a_ of around 10, while the pK_a_ of cysteine is 8.3. The pH may also have been affected by the polarity of the peptides. According to the classification of amino acids, F, W, Y, and M have a moderately hydrophobic character, and only cysteine has polar properties. 

Analysis of the surface wettability of films formed by the selected peptides showed hydrophilic properties (Table 2).

Hydrophilicity and surface properties are important features for any biomaterial in contact with cells. Hydrophilic properties are known to have a positive effect on the adhesion and activity of many types of cell, as a result of greater adsorption [51,52]. Analysis of the contact angle of the peptide films revealed that all of the tested peptide structures had very high surface hydrophilicity. Nevertheless, there were significant differences between them. The structure of the peptide, and hence the arrangement of individual amino acid residues in the peptide chain, is of great importance. The films of peptides containing tyrosine and cysteine/methionine residues (samples H–TyrTyrCys–OH (**9**) and H–TyrTyrMet–OH (**15**)) showed the lowest contact angles, between 11.2° and 14.3°, and hence the highest surface hydrophilicity. Peptides built from tryptophan had significantly higher contact angles, above 40°. Thus, the design of the primary peptide structure provides an opportunity for obtaining materials with different degrees of hydrophilicity, which is very important for the production of biomaterials for use in the regeneration of various types of tissue.

**Figure 7:**
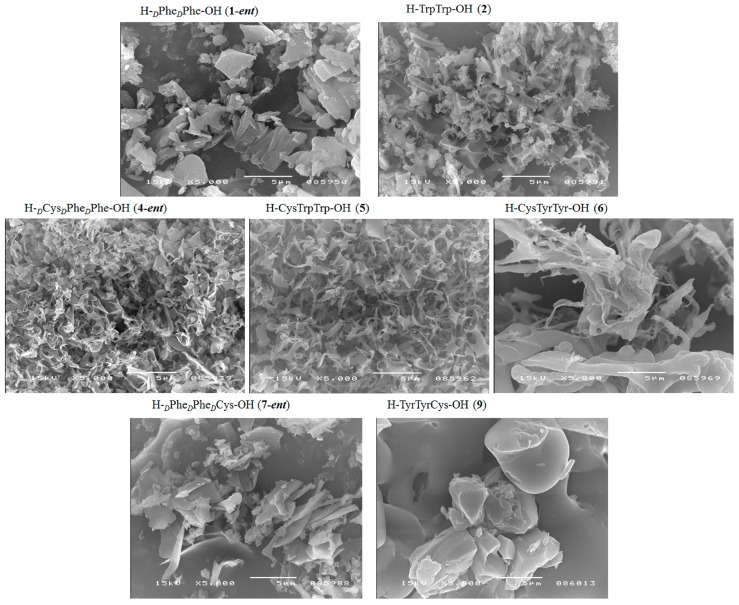

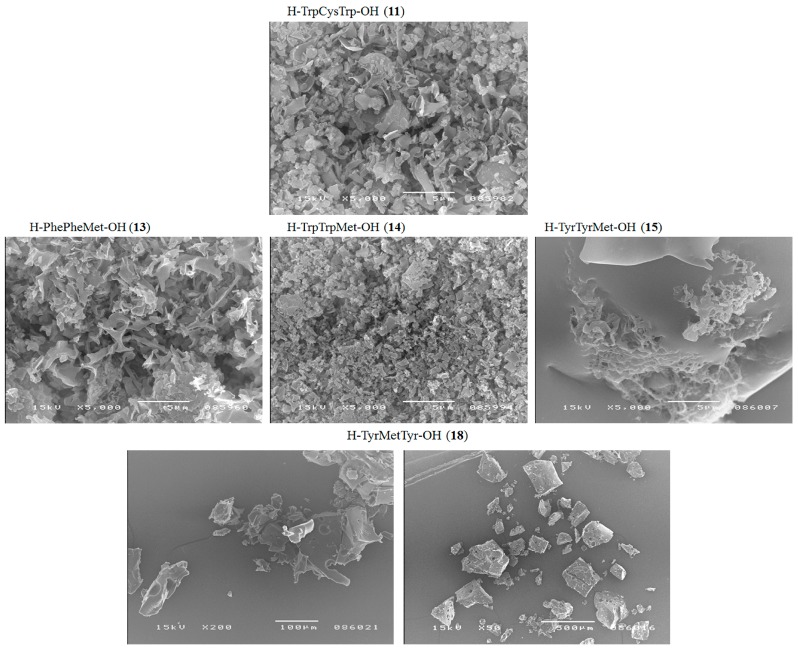
3D structures of selected peptides, scanning electron microscopy images.

Microscopic examination of the short peptide structures using scanning electron microscopy showed differences in the susceptibility of the materials to the formation of 3D spatial structures. Both highly porous structures and separated crystalline spatial structures were observed (Figure 7). It can be concluded that it may be possible to create biomaterials with complex architectures, with porous structures (appropriate pore distributions) and with the degree of roughness required for proper interactions at the cell–biomaterial interface [53], through correct selection of the primary peptide structure and careful design and control of synthesis and self-organization of the biomaterial surface. Peptide materials could therefore be used as one of the layers in advanced functional implant material. 

**Figure 8:**
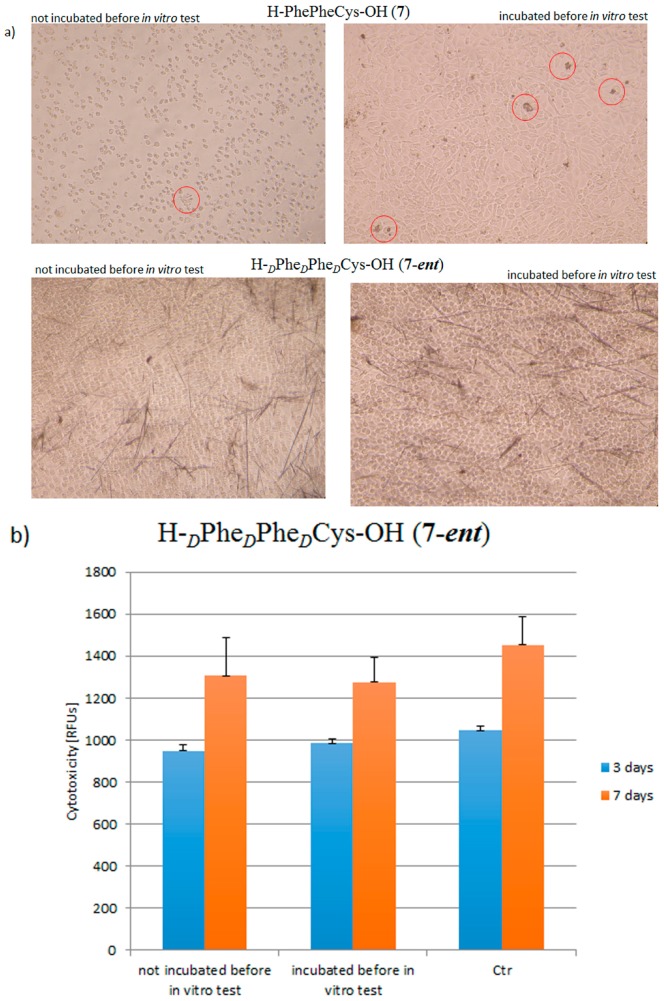
(**a**) Microscopic examination of murine L919 fibroblast cells incubated in the presence of pre-aggregated and aggregating peptides **7** and **7-*ent***. Images taken on the fourth day of incubation; (**b**) results of PrestoBlue assay evaluating the vitality of L919 fibroblast cells incubated in the presence of peptide materials formed by the **7-*ent*** peptide (RFUs—Relative Fluorescence Units).

In vitro tests were performed on the morphology of murine L919 fibroblast cells in the presence of **7** and **7-*ent*** peptides. The fibroblasts involved in the synthesis of extracellular matrix and collagen, a natural cell environment, provide the structural integrity of connective tissues. In addition, fibroblasts morphology (a branched cytoplasm surrounding an elliptical or speckled nucleus) can be used for microscopic examination of the influence of peptide aggregates. The purpose of this phase of the study was to compare the effects on morphology and cell viability of pre-aggregated peptides and of material formed by self-assembly directly in the culture medium. Two types of material were used: peptides pre-aggregated before in vitro testing and peptides not incubated before in vitro testing. The pre-aggregation conditions were as follows: the peptides were dissolved in 1,1,1,3,3,3-hexafluoro-2-propanol (HFIP), concentration = 100 mg/mL and diluted with water to a concentration of 2 mg/mL. Aggregation was carried out for 72 h at room temperature. The samples were then lyophilized to ensure the removal of HIPF. The results are shown in Figure 8a.

In the images of the **7-*ent*** peptide formed directly in the culture medium, fibrous structures were visible in significantly larger quantities than when incubation was performed prior to the in vitro tests. The case was different for peptide **7** (composed entirely of proteinogenic amino acids). The additional incubation step caused agglomeration of the peptide and the formation of clusters of aggregates marked on the picture by a circle. For the same peptide, without incubation prior to the in vitro test, only a few fibrous structures were visible. However, it is important to note that no cytotoxic effect was observed with either method. Microscopic examination on the fourth day of the in vitro assay showed normal cell morphology in both cases. Thus, regardless of how the peptide material was prepared, no negative effect on the cells was observed. A PrestoBlue assay was performed on materials formed by **7-*ent*** peptide. It was found that the peptide materials were neither toxic nor influenced the viability of the L919 cells (Figure 8b).

The last stage of the study examined the cytotoxicity of the selected peptides. Proteins and peptides that form amyloid deposits are known to be cytotoxic [54,55]. The selected peptides were tested for cytotoxicity on the endothelial cell line EA.hy 926. The choice of the endothelial cell line EA.hy 926 to study the cytotoxicity of all selected peptides was due to the fact that Human Endothelial Cell Line can be used to study the activation and response of immune system [56,57]. Peptides and proteins are one of the most potent immunogenic factors, which is a disadvantage of potential peptide/protein scaffolds. Cytotoxicity tests were performed in five replications. The presented results are an average value. Low values of standard deviation indicate high reproducibility of the obtained data. For all tested peptides, except H–TrpTrpMet–OH (**14**), statistically significant results (marked with star) were found.

**Figure 9:**
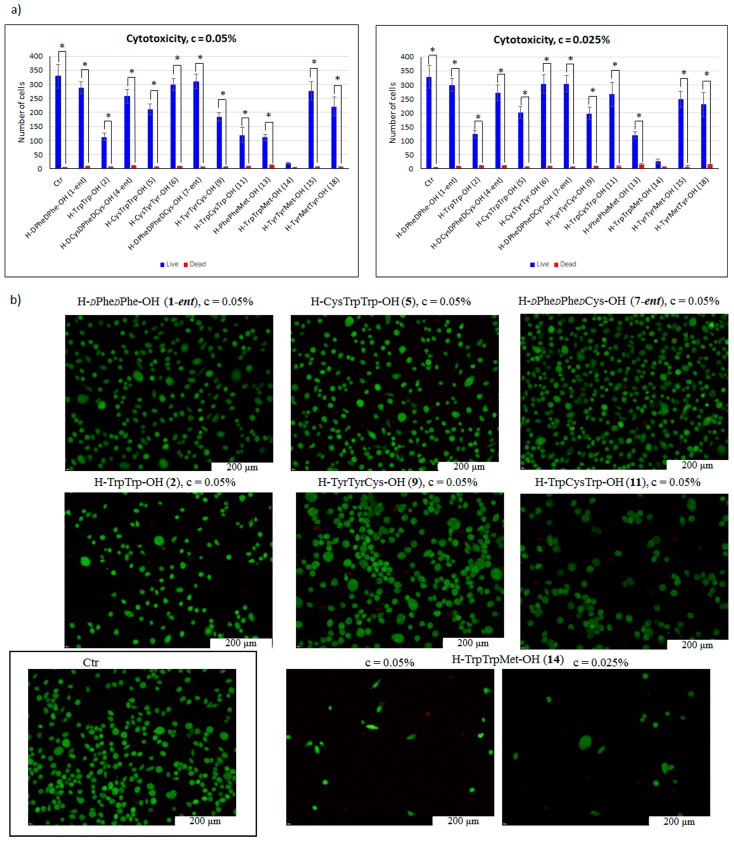
(**a**) Cytotoxicity of selected peptides at two different concentrations (c = 0.05% and 0.025%), determined for the endothelial cell line EA.hy 926 using the live/dead assay, statistically significant results are marked with star; (**b**) two fluorescent dyes: Calcein AM (green color for live cells) and ethidium homodimer-1 (EthD-1, stains dead cells red). Additional statistical data are presented in Supporting Materials.

The most cytotoxic peptide, which inhibited the proliferation of the endothelial cell line EA.hy 926 in both tested concentrations, was H–TrpTrpMet–OH (**14**) (Figure 9a). Significant toxicity was also observed for peptides **2**, **11**, and **15** at a concentration of c = 0.05% and for peptides **2** and **13** at a concentration of 0.025%. Importantly, two residues of tryptophan are present in both peptides **2** and **11**. Clear inhibition of proliferation of EA.hy 926 is visible in Figure 9b (minimal amount of live cells). With other peptides, low or moderate cytotoxicity was observed. Due to their low cytotoxicity, the following peptides showed potential for use in regenerative medicine: **1-*ent***, **4-*ent***, **6**, **7-*ent***, and **15**.

## 3. Materials and Methods

### 3.1. General Information

Fmoc-protected amino acids were purchased from Novabiochem (San Diego, CA, USA) or Bachem AG (Bubendorf, Switzerland). Anal. HPLC. Performed on a Waters HPLC system (Waters Corporation, Milford, MA, USA), using a Kintex 2.6u, C18, 100A column (100 × 4.6 mm) with a gradient of 0.1% TFA in H_2_O (B) and 0.1% TFA in CH_3_CN (A), at a flow rate of 0.4 mL/min with UV detection at 220 and 254 nm.

MS analysis. Performed on MS Bruker (Bruker Corporation, Billerica, MA, USA) micrOTOF-QIII spectrometer.

### 3.2. Peptide Synthesis

Peptides H–PhePhe–OH (**1**), H–*d*Phe*d*Phe–OH (**1-*ent***), H–TrpTrp–OH (**2**), H–TyrTyr–OH (**3**), H–*d*Cys*d*Phe*d*Phe–OH (**4-*ent***), H–CysTrpTrp–OH (**5**), H–CysTyrTyr–OH (**6**), H–PhePheCys–OH (**7**), H–*d*Phe*d*Phe*d*Cys–OH (**7-*ent***), H–TrpTrpCys–OH (**8**), H–TyrTyrCys–OH (**9**), H–PheCysPhe–OH (**10**), H–TrpCysTrp–OH (**11**), H–TyrCysTyr–OH (**12**), H–PhePheMet–OH (**13**), H–TrpTrpMet–OH (**14**), H–TyrTyrMet–OH (**15**), H–PheMetPhe–OH (**16**), H–TrpMetTrp–OH (**17**), H–TyrMetTyr–OH (**18**) were synthesized in a syringe reactor using Fmoc methodology. All peptides were synthesized in an appropriate glass reactor or a syringe.

#### Manual Solid-Phase Peptide Synthesis (SPPS)

Loading of the 2-Chlorotrityl Chloride Resin (GP 1): The amino acid (3 equiv. rel. to the resin) and 6 equiv. of DIPEA were dissolved in CH_2_Cl_2_ (10 mL per 1 g of the resin), containing, if necessary, a small amount of DMF to facilitate dissolution of the amino acid. The 2-chlorotrityl chloride resin was pre-swollen in CH_2_Cl_2_ for 1 h, and, after that the solution containing the amino acid was added. The resin was shaken for 30–120 min, then washed three times with CH_2_Cl_2_/MeOH/DIPEA (17:2:1), twice with DMF and three times with CH_2_Cl_2_.

Standard Coupling Procedure (GP 2): Three equiv. of amino acid, 3 equiv. of 4-(4,6 dimethoxy-[1,3,5]triazin-2-yl)-4-methylmorpholinium toluene-4-sulfonate (DMT/NMM/TosO^−^) and 6 equiv. of NMM were mixed and added to the resin. The resin was shaken for 1–2 h. The Kaiser test was used to confirm completion of the reaction [58].

Deprotection (GP 3): The Fmoc protecting group was removed using a solution of 20% piperidine in DMF (2 × 5 min).

Cleavage from the Resin (GP 4): The peptides were cleaved from the resin using TFA/Et_3_SiH/H_2_O 95:2.5:2.5 (ca. 2 mL/0.1 g of resin). Cleavage was performed over 4 h. The resin was filtered off and the filtrate evaporated. To precipitate the peptide, Et_2_O was added to the oily residue. The resulting solid was filtered off, washed with Et_2_O, dried and lyophilized. 

Cleavage from the Resin (GP 4A): The peptides were cleaved from the resin using TFA/EDT/Et_3_SiH/H_2_O 94:2.5:2.5:1 (ca. 2 mL/0.1 g of resin). Cleavage was performed over 4 h. The resin was filtered off and the filtrate evaporated. To precipitate the peptide, Et_2_O was added to the oily residue. The resulting solid was filtered off, washed with Et_2_O, dried and lyophilized.

### 3.3. Aggregation Studies

#### 3.3.1. Spectroscopic Measurements with Congo Red

To initiate the aggregation process, peptide samples were dissolved in 1 mL of phosphate buffer (0.1 M, pH 7.2) and incubated for 7 days at 37.4 °C. The final concentration of the incubated peptides was 1.44 mM. The samples were then treated with 1 mL of Congo Red (c = 45 μM, PBS, pH 7.2). The samples were incubated for a further 4 days at rt, during which absorbance was measured in the wavelength range of 800–400 nm. A solution of Congo Red (c = 45 μM, PBS, pH 7.2) was also incubated for 4 days at RT and was used as the reference. All measurements were performed using a Hitachi UV spectrophotometer (Hitachi, Tokyo, Japan).

#### 3.3.2. Spectroscopic Measurements with Thioflavin T

To initiate the aggregation process, peptide samples were incubated for 7 days at 37.4 °C in 2 mL of PBS (0.1 M, pH 6.0), c = 0.658 mM. Two mL of Thioflavin T solution (c = 57 mM, PBS, pH 6.0) was then added to each sample. The samples were incubated for a further 4 days, during which the fluorescence intensity was measured daily, in the wavelength range of 470–600 nm (λexcit = 440 nm). A solution of Thiofavin T (c = 57 mM, PBS, pH 6.0), also incubated for 4 days at RT (room temperature), was used as the reference. All measurements were performed using a FLUOROMAX-3 from Horiba Scientific (Edison, NJ, USA), in the wavelength range of 470–600 nm.

#### 3.3.3. Peptide Analysis under Polarized Light

A sample for microscopic analysis (peptide and Congo Red solution) was centrifuged at 12,000–14,000 rpm in a centrifuge tube to pellet the fibrils, then washed three times with water. The fibrils were then suspended in a small amount of water and placed on a glass microscope slide. The sample was air-dried and analyzed under polarized light using a Delta Optical Genetic Pro microscope.

### 3.4. Surface Properties of Peptides

The contact angle was determined using the sitting drop method with a DSA10 Kruss goniometer. Between 7 and 10 drops (0.25–0.35 μL) were applied to each of the peptide films.

SEM studies were performed using a Bruker MultiMode V microscope in Tapping Mode, using antimony doped silicon cantilevers with a spring constant of 40 N/m and a nominal tip diameter of 8 nm.

### 3.5. Stability and Biological Properties of Peptide Materials

#### 3.5.1. Stability of Peptides under In Vitro Conditions

The properties of the peptides in the culture medium (Dulbecco EAGLE MEM) and in buffered saline solution (PBS) were monitored using pH measurements. Peptide extracts (1:100) were incubated under in vitro conditions (37 °C/5% CO_2_/1 msc) and the pH values measured each week.

#### 3.5.2. Cell Cytotoxicity Test

Preparation of samples: The peptides were suspended in 70% ethanol and dispensed to the bottom of a sterile test plate, reaching final concentrations of 0.05% and 0.025%. When the solvent at the bottom of the wells was evaporated, a peptide layer was obtained.

Cell culture: The peptides were tested in contact with the endothelial cell line EA.hy 926 (ATCC, Manassas, VA, USA) [59]. The cells were cultured in Dulbecco’s Modified Eagle’s Medium, containing l-glutamine (4 mM), glucose (4.5 g/L), 15% fetal bovine serum (Biological Industries) and 0.5% mixtures of streptomycin sulphate and penicillin G (Pen: 10.000 UmL^−1^; Strep: 10 mgmL^−1^; Biological Industries) under standard conditions (37 °C, a humidified atmosphere of 5% CO_2_ in air). The cells were seeded into wells containing the peptides with an initial seeding density of 3.0 × 10^5^ cells/cm^3^. The culture lasted for 48 h.

PrestoBlueTM Cell Cytotoxicity assay: The cytocompatibility of the peptides was analyzed after 3 and 7 days of the culture with the use of PrestoBlueTM Cell Viability assay (Invitrogen, Carlsbad, CA, USA). This assay is useful for rapid evaluation the viability and proliferation of a wide range of cell types. PrestoBlue™ (Thermo Fisher Scientific, Waltham, MA, USA) reagent is quickly reduced by metabolically active cells, providing a quantitative measure of viability and cytotoxicity. PrestoBlueTM Cell Viability assay was used to determine the amount of intracellular redox reaction of resazurin into fluorescent resorufin which corresponded to cells viability. In compliance with the manufacturer’s protocols, 20 μL of the PrestoBlue reagent was added per well and plates were returned to an incubator for 1 h. The fluorescence was read at an excitation/emission wavelength of 560/590 nm on the microplate reader POLARstar Omega (BMG Labtech, Ortenberg, Germany).

Live/dead assay: To evaluate cell viability the ‘live/dead’ test was performed, using a Live/Dead Viability/Cytotoxicity Kit (Molecular Probes, Thermo Fisher Scientific, Waltham, MA, USA). This test is based on two fluorescent dyes: Calcein AM (showing a green color for live cells) and ethidium homodimer-1 (EthD-1, which stains dead cells red). The ‘live/dead’ test was performed according to the manufacturer’s instructions. Briefly, the endothelial cells were cultured in the presence of peptides for 48 h. The samples were then washed twice with phosphate-buffered saline (PBS) and incubated in a solution of dyes (0.6 μM Calcein AM1 and 1.5 μM EthD-1) for 30 min at 37 °C. The stain was removed by washing with PBS. Microscopic observations were then made using a fluorescence microscope (Olympus GX71, Olympus, Tokyo, Japan).

The live and dead cells were counted using the Image J program. The results have been presented as a mean ± standard deviation (SD). The statistical analysis of the obtained results was carried out using one-way analysis of variance (ANOVA). Results with *p* > 0.05 were considered to be statistically significant.

#### 3.5.3. Cytotoxicity Test

Cell Culture: The mouse fibroblast cell line L929 (ATCC, American Type Culture Collection (ATCC), Manassas, VA, USA) was expanded in 75 cm^2^ tissue culture flasks with Eagle’s Minimum Essential Medium supplemented with 10% horse serum (ATCC, Manassas, VA, USA) at 37 °C in a humidified, 5% CO_2_ atmosphere. The medium was changed every 3 days until a 70% confluent cell monolayer developed. The cells were then detached from the culture flasks using 5% Trypsin-EDTA (GE Healthcare—HyClone Laboratories Inc., Logan, UT, USA). After flushing and centrifugation, the cells were concentrated to 5 × 10^4^ cells/mL in the culture medium. Next, 200 μL of the cell suspension was placed into wells in 96-well culture plates (ThermoSci, Nunc, Roskilde, Denmark). The cells were allowed to adhere for 24 h, before 10 μL of peptide suspension was added to each well. The peptides **7** and **7-*ent***, in the form of powder, were resuspended in PBS (10 μg/mL). The study was conducted for 3 and 7 days. The TCPS plates served as a positive control.

Cell Morphology: Three cultures of L929 cells were stained with 0.01% acridine orange (AO) for 1 min. The samples were next rinsed in PBS, observed and photographed under an Olympus CX41 (Olympus, Tokyo, Japan) fluorescence microscope to evaluate their morphology and attachment to the examined materials.

## 4. Conclusions

Understanding the complex relations between the structure of di- and tripeptides and their susceptibility for self-assembly remains a challenge. Self-aggregating fibrous structures could be used as scaffolds in regenerative medicine. However, they require careful design and sequencing, taking into account the multidimensionality of the scaffold structure, its mechanical parameters, cytocompatibility, toxicity, and susceptibility to degradation under physiological conditions into bioneutral metabolites. In this study, aggregation susceptibility tests were conducted on a pool of 20 di- and tripeptides, composed of aromatic amino acids and cysteine/methionine. Twelve of the compounds formed amyloid-like fibrous structures. The selected short peptides, composed of *l* or *d* amino acids, were tested with regard to their usefulness as scaffolds, taking into account the form, hydrophilicity, and toxicity of the aggregates. 

The cytotoxicity of the peptides was tested on the endothelial cell line EA.hy 926. The peptides that did not fulfil the standards were: H–TrpTrpMet–OH (**14**), H–TrpTrp–OH (**2**), H–TrpCysTrp–OH (**11**), and H–PhePheMet–OH (**13**). Those that showed potential for use in regenerative medicine were H–*d*Phe*d*Phe–OH (**1-*ent***), H–*d*Cys*d*Phe*d*Phe–OH (**4-*ent***), H–CysTyrTyr–OH (**6**) and H–*d*Phe*d*Phe*d*Cys–OH (**7-*ent***). This group may be expanded to include H–TyrTyrMet–OH (**15**) and H–TyrMetTyr–OH (**18**), in which cysteine is replaced with methionine residue. Significantly, none of the selected materials were derived from tryptophan peptides. The influence of peptide aggregates on the morphology of L919 fibroblast cells was also studied. Preliminary findings suggest that the stereochemistry of the peptide had no effect on either the morphology or viability of the cells. This is particularly important because, in the case of peptides consisting of *d* amino acids, increased resistance to proteolytic enzymes might be expected, whereas in amyloid structures and in the case of systems composed of natural *l* amino acids, increased resistance to proteases was observed.

## Figures and Tables

**Table 1:** Results of susceptibility to aggregation of peptides **1**–**18**.

Peptide	CR Assay	ThT Assay	Microscopic Examination, Morphology
H–PhePhe–OH (**1**)	+	+	+ fibrous structure
H–*d*Phe*d*Phe–OH (**1-*ent***)	+	+	+ fibrous structure
H–TrpTrp–OH (**2**)	+	+	+ fibrous structure
H–TyrTyr–OH (**3**)	+/−	+	+/− cluster of fibrous and amorphous structures
H–*d*Cys*d*Phe*d*Phe–OH (**4-*ent***)	+	+	+ fibrous structure
H–CysTrpTrp–OH (**5**)	+	+	+ fibrous structure
H–CysTyrTyr–OH (**6**)	+	+	+ fibrous structure
H–PhePheCys–OH (**7**)	+/−	+/−	+/− cluster of fibrous and amorphous structures
H–*d*Phe*d*Phe*d*Cys–OH (**7-*ent***)	+	+	+ fibrous structure
H–TrpTrpCys–OH (**8**)	+/−	+	+/− sphere
H–TyrTyrCy–OH (**9**)	+	+	+ fibrous structure
H–PheCysPhe–OH (**10**)	+	+	+/− cluster of fibrous and amorphous structures
H–TrpCysTrp–OH (**11**)	+	+	+/− fibrous structure
H–TyrCysTyr–OH (**12**)	−	−	−
H–PhePheMet–OH (**13**)	+	+	+ fibrous structure
H–TrpTrpMet–OH (**14**)	+/−	+/−	+/− cluster of fibrous and amorphous structures
H–TyrTyrMet–OH (**15**)	+/−	+/−	+/− cluster of fibrous and amorphous structures
H–PheMetPhe–OH (**16**)	+/−	+/−	+/− cluster of fibrous and amorphous structures
H–TrpMetTrp–OH (**17**)	+/−	−	− amorphous structures
H–TyrMetTyr–OH (**18**)	+/−	+/−	+/− cluster of fibrous and amorphous structures

**Table 2:** Surface wettability of films formed by selected peptides.

Peptide	THETA (°) ^1^
H–*d*Phe*d*Phe–OH (**1-*ent***)	34.1
H–TrpTrp–OH (**2**)	42.1
H–*d*Cys*d*Phe*d*Phe–OH (**4-*ent***)	39.2
H–CysTrpTrp–OH (**5**)	46.8
H–CysTyrTyr–OH (**6**)	14.3
H–*d*Phe*d*Phe*d*Cys–OH (**7-*ent***)	40.1
H–TyrTyrCys–OH (**9**)	11.2
H–TrpCysTrp–OH (**11**)	49.3
H–PhePheMet–OH (**13**)	27.5
H–TrpTrpMet–OH (**14**)	20.2
H–TyrTyrMet–OH (**15**)	11.6
H–TyrMetTyr–OH (**18**)	12.7

^1^ Average values of 5 measurements.

## Supplementary Materials

Supplementary materials are available online. Information contained all details concerning the synthesis of peptides **1**–**18**.
