# Supplementary material for: Search for Fibrous Aggregates Potentially Useful in Regenerative Medicine Formed under Physiological Conditions by Self-Assembling Short Peptides Containing Two Identical Aromatic Amino Acid Residues
Source: Molecules. 2018 Mar 2;23(3):568. doi: 10.3390/molecules23030568 (PMC6017032; doi:10.3390/molecules23030568)
Supplement: Supplementary file 1 [file molecules-23-00568-s001.pdf]

Supporting Materials

Search for Fibrous Aggregates Potentially Useful in Regenerative Medicine Formed under Physiological Conditions by Self-Assembling Short Peptides Containing Two Identical Aromatic Amino Acid Residues

Justyna Fraczyk [1], Wojciech Lipinski [1], Agata Chaberska [1], Joanna Wasko [1], Kamil Rozniakowski [1], Zbigniew J. Kaminski [1], Maciej Bogun [2], Zbigniew Draczynski [2], Elzbieta Menaszek [3], Ewa Stodolak-Zych [4], Marta Kaminska [5], and Beata Kolesinska [1,*]

[1] Institute of Organic Chemistry, Lodz University of Technology, Zeromskiego 116, 90-924 Lodz, Poland
[2] Department of Material and Commodity Sciences and Textile Metrology, Lodz University of Technology, Zeromskiego 116, 90-924 Lodz, Poland
[3] Department of Cytology, CMUJ — Jagiellonian University Medical College, Swietej Anny 12, 31-008 Krakow, Poland
[4] AGH - University of Science and Technology, Department of Biomaterials, A. Mickiewicz 30, 30-059 Krakow, Poland
[5] Division of Biophysics, Institute of Materials Science and Engineering, Lodz University of Technology, Stefanowskiego 1/15, 90-924 Lodz, Poland

* Correspondence: beata.kolesinska@p.lodz.pl; Tel.: +48-42-631-3149

Synthesis of H–PhePhe–OH (**1**)

2-Chlorotrityl chloride resin (0.5 g, 1.0 mmol/g, 0.5 mmol) was esterified with Fmoc-Phe–OH (0.581 g, 1.5 mmol) in the presence of DIPEA (540 μL, 3.0 mmol) according to (GP 1), followed by Fmoc deprotection (GP 3). Subsequently the peptides chains were elongated (GP 2), respectively, with Fmoc–Phe–OH (0.581 g, 1.5 mmol) in the presence DMT/NMM/TosO$^-$ (0.619 g, 1.5 mmol) and NMM (330 μL, 3.0 mmol). After the last deprotection (GP 3), the peptide was cleaved from the resin (GP 4). Anal. RP-HPLC (3–97%B in 45 min): t_R 17.68 min, purity 98.9%. LC/MS: 313.6 ([M + H]$^+$, $C_{18}H_{20}N_2O_3^+$; calc. 312.37).

Synthesis H–*D*Phe*D*Phe–OH (**1-*ent***)

Starting materials: 2-Chlorotrityl chloride resin (0.5 g, 1.0 mmol/g, 0.5 mmol) was esterified with Fmoc-Phe–OH (0.581 g, 1.5 mmol) in the presence of DIPEA (540 μL, 3.0 mmol) according to (GP 1), followed by Fmoc deprotection (GP 3). Subsequently the peptides chains were elongated (GP 2), respectively, with Fmoc–Phe–OH (0.581 g, 1.5 mmol) in the presence DMT/NMM/TosO$^-$ (0.619 g, 1.5 mmol) and NMM (330 μL, 3.0 mmol). After the last deprotection (GP 3), the peptide was cleaved from the resin (GP 4). Anal. RP-HPLC (3–97%B in 45 min): t_R 17.66 min., purity = 99.52%. LC-MS: 313.61 ([M + H]$^+$, $C_{18}H_{21}N_2O_3^+$, calc. 313.49).

Synthesis of H–TrpTrp–OH (**2**)

Starting materials: 2-chlorotrityl chloride resin (0.5 g, 1.0 mmol/g, 0.5 mmol), Fmoc–Trp(Boc)–OH (0.790 g, 1.5 mmol), DIPEA (540 μL, 3.0 mmol), Fmoc–Trp(Boc)–OH (0.790 g, 1.5 mmol),

DMT/NMM/TosO⁻ (0.619 g, 1.5 mmol) and NMM (330 μL, 3.0 mmol). The peptide was cleaved from the resin according to GP 4. Product: H–TrpTrp–OH (**2**). Anal. RP-HPLC (3–97%B in 45 min): t_R 17.36 min, purity 97.4%. LC/MS: 391.6 ([M + H]$^+$, $C_{22}H_{22}N_4O_3^+$; calc. 390.45).

Synthesis H–TyrTyr–OH (**3**)

Starting materials: 2-chlorotrityl chloride resin (0.5 g, 1.0 mmol/g, 0.5 mmol), Fmoc–Tyr(tBu)–OH (0.689 g, 1.5 mmol), DIPEA (540 μL, 3.0 mmol), Fmoc–Tyr(tBu)–OH (0.689 g, 1.5 mmol), DMT/NMM/TosO⁻ (0.619 g, 1.5 mmol) and NMM (330 μL, 3.0 mmol). The peptide was cleaved from the resin according to GP 4. Product: H–TyrTyr–OH (**3**). Anal. RP-HPLC (3–97%B in 45 min): t_R 12.38 min, purity 98.9%. LC/MS: 345.5 ([M + H]$^+$, $C_{18}H_{20}N_2O_5^+$;calc. 344.37).

Synthesis of H–*D*Cys*D*Phe*D*Phe–OH (**4-*ent***)

Starting materials: 2-chlorotrityl chloride resin (0.5 g, 1.0 mmol/g, 0.5 mmol), Fmoc–*D*Phe–OH (0.581 g, 1.5 mmol), DIPEA (540 μL, 3.0 mmol), Fmoc–*D*Phe–OH (0.581 g, 1.5 mmol), Fmoc–*D*Cys(Trt)–OH (0.878 g, 1.5 mmol), 2 × DMT/NMM/TosO⁻ (0.619 g, 1.5 mmol), 2 × NMM (330 μL, 3.0 mmol). The peptide was cleaved from the resin according to GP 4A. Product: H–*D*Cys*D*Phe*D*Phe–OH (**4-*ent***). Anal. RP-HPLC (3–97%B in 45 min): t_R 13.62 min, purity 96.9%. LC/MS: 416.1684 ([M + H]$^+$, $C_{21}H_{25}N_3O_4S^+$; calc. 415.52).

Synthesis of H–CysTrpTrp–OH (**5**)

Starting materials: 2-chlorotrityl chloride resin (0.5 g, 1.0 mmol/g, 0.5 mmol), Fmoc–Trp(Boc)–OH (0.790 g, 1.5 mmol), DIPEA (540 μL, 3.0 mmol), Fmoc–Trp(Boc)–OH (0.790 g, 1.5 mmol), Fmoc–Cys(Trt)–OH (0.878 g, 1.5 mmol), 2 × DMT/NMM/TosO⁻ (0.619 g, 1.5 mmol), 2 × NMM (330 μL, 3.0 mmol). The peptide was cleaved from the resin according to GP 4A. Product: H–CysTrpTrp–OH (**5**). Anal. RP-HPLC (3–97%B in 45 min): t_R 14.01 min, purity 99.2%. LC/MS: 494.1904 ([M + H]$^+$, $C_{25}H_{27}N_5O_4S^+$; calc. 493.59).

Synthesis of H–CysTyrTyr–OH (**6**)

Starting materials: 2-chlorotrityl chloride resin (0.5 g, 1.0 mmol/g, 0.5 mmol), Fmoc–Tyr(tBu)–OH (0.689 g, 1.5 mmol), DIPEA (540 μL, 3.0 mmol), Fmoc–Tyr(tBu)–OH (0.689 g, 1.5 mmol), Fmoc–Cys(Trt)–OH (0.878 g, 1.5 mmol), 2 × DMT/NMM/TosO⁻ (0.619 g, 1.5 mmol), 2 × NMM (330 μL, 3.0 mmol). The peptide was cleaved from the resin according to GP 4A. Product: H–CysTyrTyr–OH (**6**). Anal. RP-HPLC (3–97%B in 45 min): t_R 9.21 min, purity 98.1%. LC/MS: 448.1528 ([M + H]$^+$, $C_{21}H_{25}N_3O_6S^+$; calc. 447.51).

Synthesis of H–PhePheCys–OH (**7**)

Starting materials: 2-chlorotrityl chloride resin (0.5 g, 1.0 mmol/g, 0.5 mmol), Fmoc–Cys(Trt)–OH (0.878 g, 1.5 mmol), DIPEA (540 μL, 3.0 mmol), 2 × Fmoc–Phe–OH (0.581 g, 1.5 mmol), 2 × DMT/NMM/TosO⁻ (0.619 g, 1.5 mmol), 2 × NMM (330 μL, 3.0 mmol). The peptide was cleaved from the resin according to GP 4A. Product: H–PhePheCys–OH (**7**). Anal. RP-HPLC (3–97%B in 45 min): t_R 2.01 min, purity 97.5%. LC/MS: 416.7 ([M + H]$^+$, $C_{21}H_{25}N_3O_4S^+$; calc. 415.52).

Synthesis of H–*D*Phe*D*Phe*D*Cys–OH (**7-*ent***)

The synthesis was carried out according to procedure described above. Spectroscopic data of product identical with peptide **7**.

Synthesis of H–TrpTrpCys–OH (**8**)

Starting materials: 2-chlorotrityl chloride resin (0.5 g, 1.0 mmol/g, 0.5 mmol), Fmoc–Cys(Trt)–OH (0.878 g, 1.5 mmol), DIPEA (540 μL, 3.0 mmol), 2 × Fmoc–Trp(Boc)–OH (0.790 g, 1.5 mmol), 2 × DMT/NMM/TosO⁻ (0.619 g, 1.5 mmol) and 2 × NMM (330 μL, 3.0 mmol). The peptide was cleaved

from the resin according to GP 4A. Product: H–TrpTrpCys–OH (**8**). Anal. RP-HPLC (3–97%B in 45 min): t_R 1.85 min, purity 99.7%. LC/MS: 494.6 ([M + H]$^+$, $C_{25}H_{27}N_5O_4S^+$; calc. 493.59).

Synthesis of H–TyrTyrCys–OH (**9**)

Starting materials: 2-chlorotrityl chloride resin (0.5 g, 1.0 mmol/g, 0.5 mmol), Fmoc–Cys(Trt)–OH (0.878 g, 1.5 mmol), DIPEA (540 μL, 3.0 mmol), 2 × Fmoc–Tyr(tBu)–OH (0.689 g, 1.5 mmol), 2 × DMT/NMM/TosO$^-$ (0. 619 g, 1.5 mmol) and 2 × NMM (330 μL, 3.0 mmol). The peptide was cleaved from the resin according to GP 4A. Product: H–TyrTyrCys–OH (**9**). Anal. RP-HPLC (3–97%B in 45 min): t_R 4.22 min, purity 98.9%. LC/MS: 448.6 ([M + H]$^+$, $C_{21}H_{25}N_3O_6S^+$; calc. 447.51).

Synthesis of H–PheCysPhe–OH (**10**)

Starting materials: 2-chlorotrityl chloride resin (0.5 g, 1.0 mmol/g, 0.5 mmol), Fmoc–Phe–OH (0.581 g, 1.5 mmol), DIPEA (540 μL, 3.0 mmol), Fmoc–Cys(Trt)–OH (0.879 g, 1.50 mmol), Fmoc–Phe–OH (0.581 g, 1.50 mmol), 2 × DMT/NMM/TosO$^-$ (0.619 g, 1.50 mmol) and 2 × NMM (330 μL, 3.0 mmol). The peptide was cleaved from the resin according to GP 4A. Product: H–PheCysPhe–OH (**10**). Anal. RP-HPLC (3–97%B in 45 min): t_R 1.99 min, purity 95.9%. LC/MS: 414.5 ([M − H]$^+$, $C_{21}H_{25}N_3O_4S^+$; calc. 415.52).

Synthesis of H–TrpCysTrp–OH (**11**)

Starting materials: 2-chlorotrityl chloride resin (0.5 g, 1.0 mmol/g, 0.5 mmol), Fmoc–Trp(Boc)–OH (0.790 g, 1.5 mmol), DIPEA (540 μL, 3.0 mmol), Fmoc–Cys(Trt)–OH (0.878 g, 1.5 mmol), Fmoc–Trp(Boc)–OH (0.790 g, 1.5 mmol), 2 × DMT/NMM/TosO$^-$ (0. 619 g, 1.5 mmol) and 2 × NMM (330 μL, 3.0 mmol). The peptide was cleaved from the resin according to GP 4A. Product: H–TrpCysTrp–OH (**11**). Anal. RP-HPLC (3–97%B in 45 min): t_R 1.87 min, purity 99.7%. LC/MS: 494.6 ([M + H]$^+$, $C_{25}H_{27}N_5O_4S^+$; calc. 493.59).

Synthesis of H–TyrCysTyr–OH (**12**)

Starting materials: 2-chlorotrityl chloride resin (0.5 g, 1.0 mmol/g, 0.5 mmol), Fmoc–Tyr(tBu)–OH (0.689 g, 1.5 mmol), DIPEA (540 μL, 3.0 mmol), Fmoc–Cys(Trt)–OH (0.878 g, 1.5 mmol), Fmoc–Tyr(tBu)–OH (0.689 g, 1.5 mmol), 2 × DMT/NMM/TosO$^-$ (0.619 g, 1.5 mmol) and 2 × NMM (330 μL, 3.0 mmol). The peptide was cleaved from the resin according to GP 4A. Product: H–TyrCysTyr–OH (**12**). Anal. RP-HPLC (3–97%B in 45 min): t_R 20.70 min, purity 97.3%. LC/MS: 448.6 ([M + H]$^+$, $C_{21}H_{25}N_3O_6S^+$; calc. 447.51).

Synthesis of H–PhePheMet–OH (**13**)

Starting materials: 2-chlorotrityl chloride resin (0.5 g, 1.0 mmol/g, 0.5 mmol), Fmoc–Met–OH (0.557 g, 1.5 mmol), DIPEA (540 μL, 3.0 mmol), 2 × Fmoc–Phe–OH (0.581 g, 1.5 mmol), 2 × DMT/NMM/TosO$^-$ (0.619 g, 1.5 mmol) and 2 × NMM (330 μL, 3.0 mmol). The peptide was cleaved from the resin according to GP 4A. Product: H–PhePheMet–OH (**13**). Anal. RP-HPLC (3–97%B in 45 min): t_R 22.24 min, purity 99.2%. LC/MS: 442.7 ([M − H]$^+$, $C_{23}H_{29}N_3O_4S^+$; calc. 443.57).

Synthesis of H–TrpTrpMet–OH (**14**)

Starting materials: 2-chlorotrityl chloride resin (0.5 g, 1.0 mmol/g, 0.5 mmol), Fmoc–Met–OH (0.557 g, 1.5 mmol), DIPEA (540 μL, 3.0 mmol), 2 × Fmoc–Trp(Boc)–OH (1.027 g, 1.95 mmol), 2 × Fmoc–Trp(Boc)–OH (0.790 g, 1. 5 mmol), 2 × DMT/NMM/TosO$^-$ (0.619 g, 1. 5 mmol) and NMM (330 μL, 3.0 mmol). The peptide was cleaved from the resin according to GP 4A. Product: H–TrpTrpMet–OH (**14**). Anal. RP-HPLC (3–97%B in 45 min): t_R 3.23 min, purity 99.5%. LC/MS: 522.7 ([M + H]$^+$, $C_{27}H_{31}N_5O_4S^+$; calc. 521.64).

Synthesis of H–TyrTyrMet–OH (**15**)

Starting materials: 2-chlorotrityl chloride resin (0.5 g, 1.0 mmol/g, 0.5 mmol), Fmoc–Met–OH (0.557 g, 1.5 mmol), DIPEA (540 μL, 3.0 mmol), 2 × Fmoc–Tyr(tBu)–OH (0.689 g, 1.5 mmol), 2 ×

DMT/NMM/TosO⁻ (0.619 g, 1.5 mmol) and 2 × NMM (330 μL, 3.0 mmol). The peptide was cleaved from the resin according to GP 4A. Product: H–TyrTyrMet–OH (**15**). Anal. RP-HPLC (3–97%B in 45 min): t_R 6.94 min, purity 99.5%. LC/MS: 476.6 ($[M + H]^+$, $C_{23}H_{29}N_3O_6S^+$; calc. 475.57).

Synthesis of H–PheMetPhe–OH (**16**)

Starting materials: 2-chlorotrityl chloride resin (0.5 g, 1.0 mmol/g, 0.5 mmol), Fmoc–Phe–OH (0.581 g, 1.5 mmol), DIPEA (540 μL, 3.0 mmol), Fmoc–Met–OH (0.557 g, 1.50 mmol), Fmoc–Phe–OH (0.581 g, 1.50 mmol), 2 × DMT/NMM/TosO⁻ (0.620 g, 1.50 mmol) and 2 × NMM (330 μL, 3.0 mmol). The peptides was cleaved from the resin according to GP 4A. Product: H–PheMetPhe–OH (**16**). Anal. RP-HPLC (3–97%B in 45 min): t_R 2.22 min, purity 99.2%. LC/MS: 444.7 ($[M + H]^+$, $C_{23}H_{29}N_3O_4S^+$; calc. 443.57).

Synthesis of H–Trp-Met-Trp–OH (**17**)

Starting materials: 2-chlorotrityl chloride resin (0.5 g, 1.0 mmol/g, 0.5 mmol), Fmoc–Trp(Boc)–OH (0.790 g, 1.5 mmol), DIPEA (540 μL, 3.0 mmol), Fmoc–Met–OH (0.557 g, 1.5 mmol), Fmoc–Trp(Boc)–OH (0.790g, 1.5 mmol), 2 × DMT/NMM/TosO⁻ (0.619 g, 1.5 mmol) and 2 × NMM (330 μL, 3.0 mmol). The peptide was cleaved from the resin according to GP 4A. Product: H–TrpMetTrp–OH (**17**). Anal. RP-HPLC (3–97%B in 45 min): t_R 1.95 min, purity 99.7%. LC/MS: 522.8 ($[M + H]^+$, $C_{27}H_{31}N_5O_4S^+$; calc. 521.64).

Synthesis of H–TyrMetTyr–OH (**18**)

Starting materials: 2-chlorotrityl chloride resin (0.5 g, 1.0 mmol/g, 0.5 mmol), Fmoc–Tyr(tBu)–OH (0.689 g, 1.5 mmol), DIPEA (540 μL, 3.0 mmol), Fmoc–Met–OH (0.557 g, 1.5 mmol), Fmoc–Tyr(tBu)–OH (0.689 g, 1.5 mmol), 2 × DMT/NMM/TosO⁻ (0.619 g, 1.5 mmol) and 2 × NMM (330 μL, 3.0 mmol). The peptide was cleaved from the resin according to GP 4A. Product: H–TyrMetTyr–OH (**18**). Anal. RP-HPLC (3–97%B in 45 min): t_R 1.79 min, purity 99.7%. LC/MS: 476.6 ($[M + H]^+$, $C_{23}H_{29}N_3O_6S^+$; calc. 475.57).

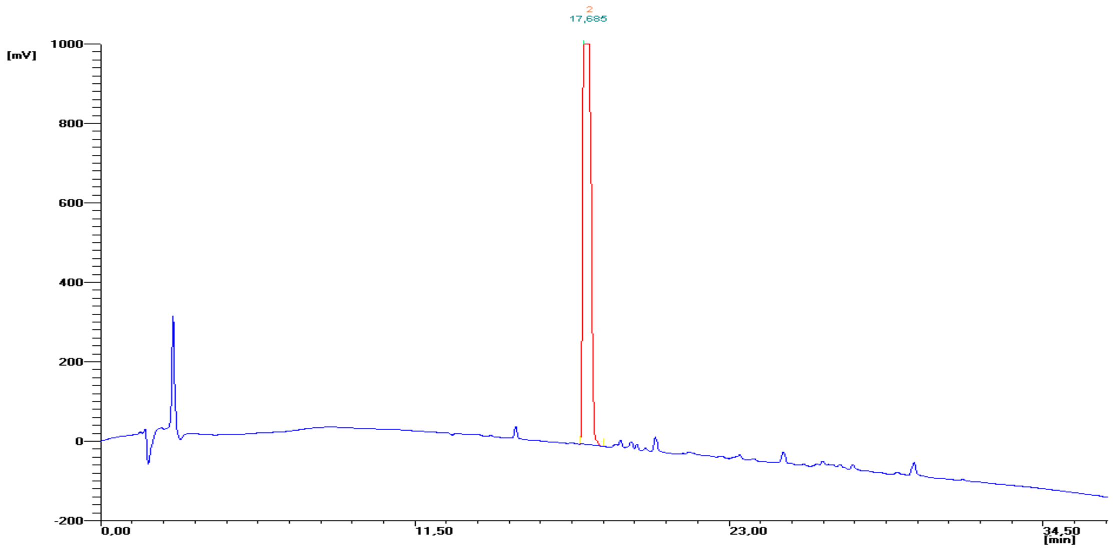

Fig. S1. HPLC spectrum of H-PhePhe-OH (**1**).

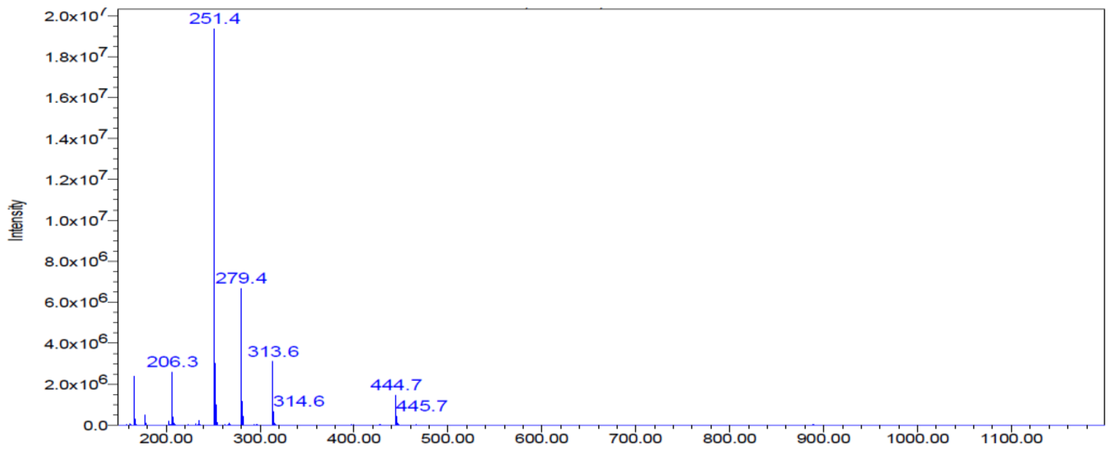

Fig. S2. MS spectrum of H-PhePhe-OH (**1**).

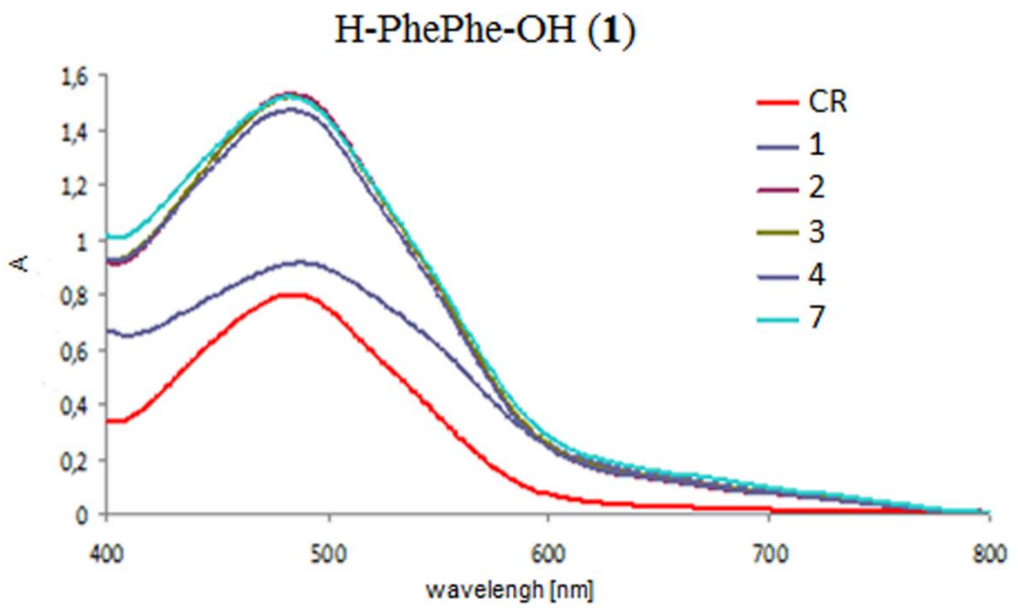

Fig. S3. UV-Vis spectra of H-PhePhe-OH (**1**), incubation with CR.

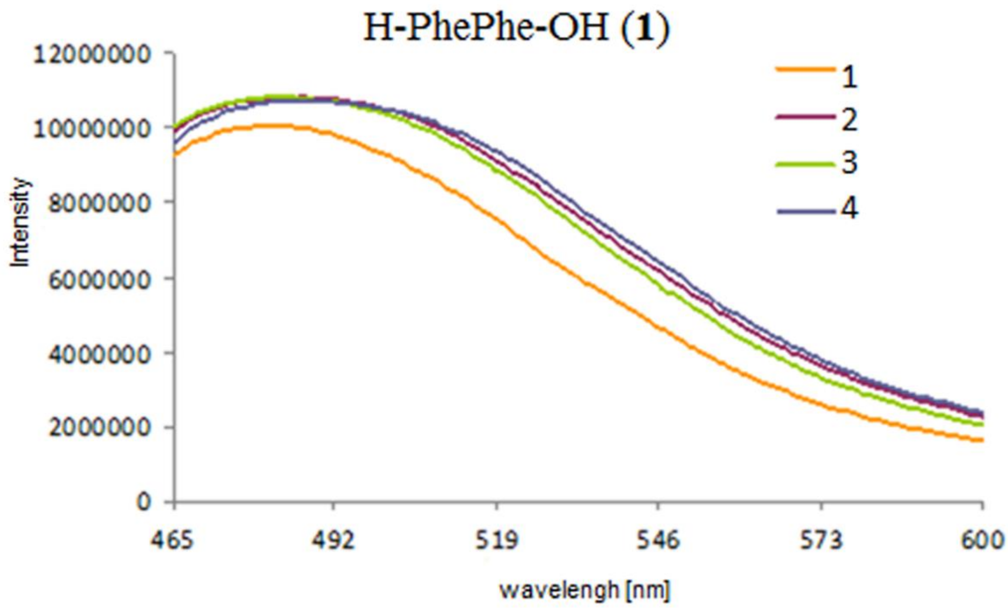

Fig. S4. Fluorescence spectra of H-PhePhe-OH (**1**), incubation with ThT.

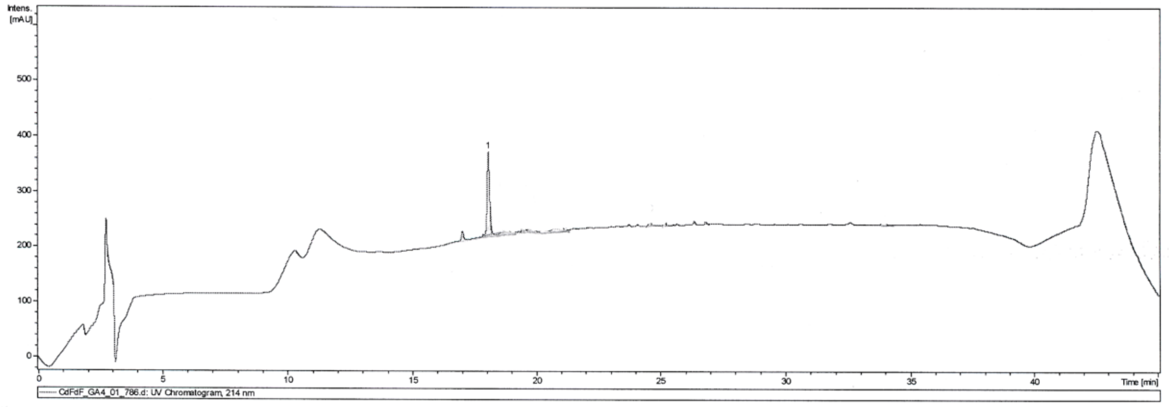

Fig. S5. HPLC spectrum of H-DPheDPhe-OH (**1-*ent***).

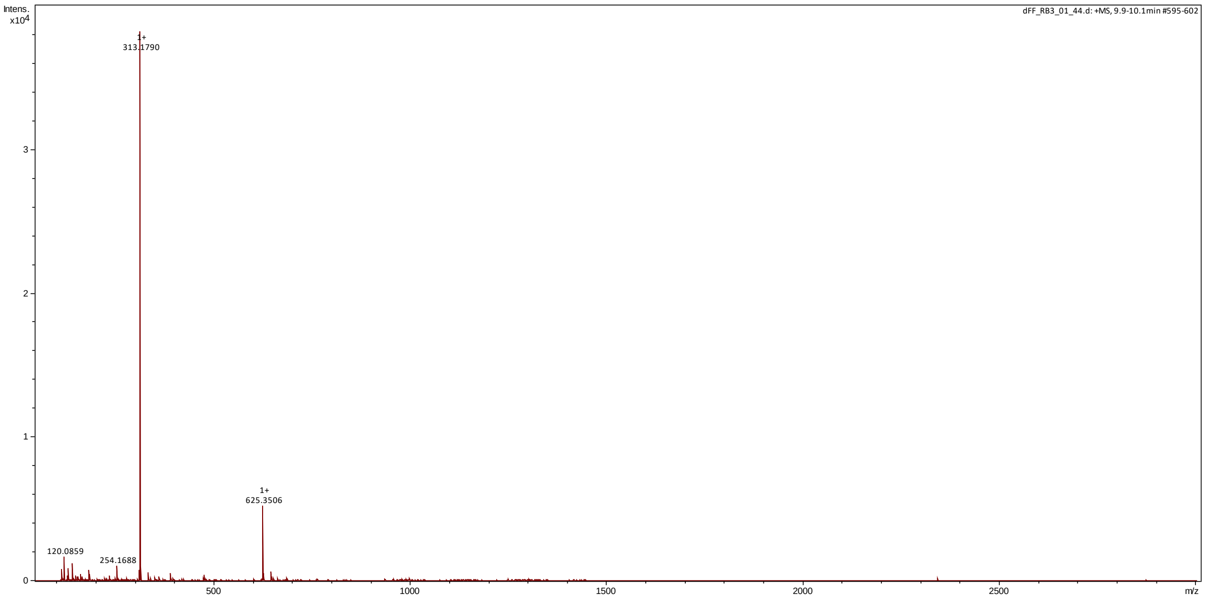

Fig. S6. MS spectrum of H-*D*Phe*D*Phe-OH (**1-*ent***).

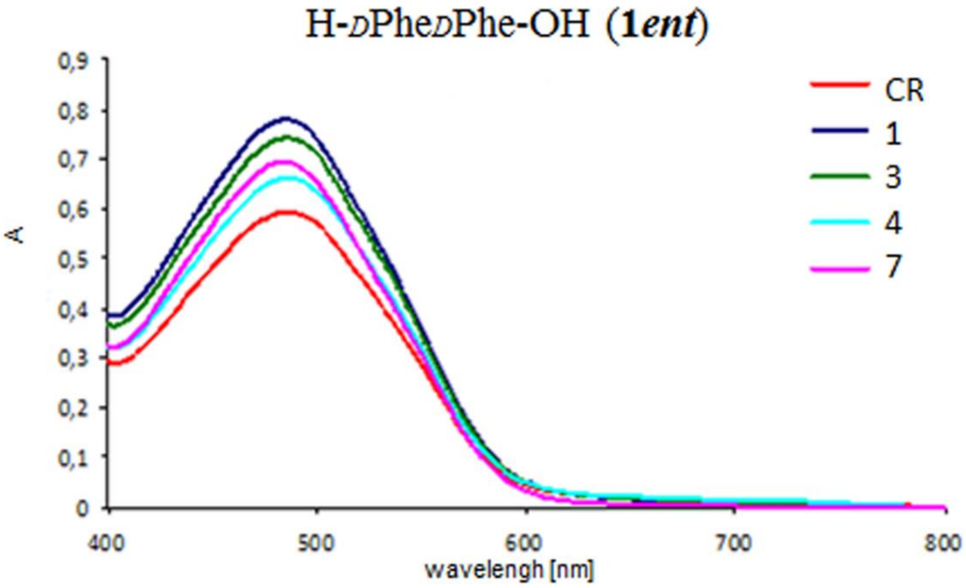

Fig. S7. UV-Vis spectra of H-*D*Phe*D*Phe-OH (**1-*ent***), incubation with CR.

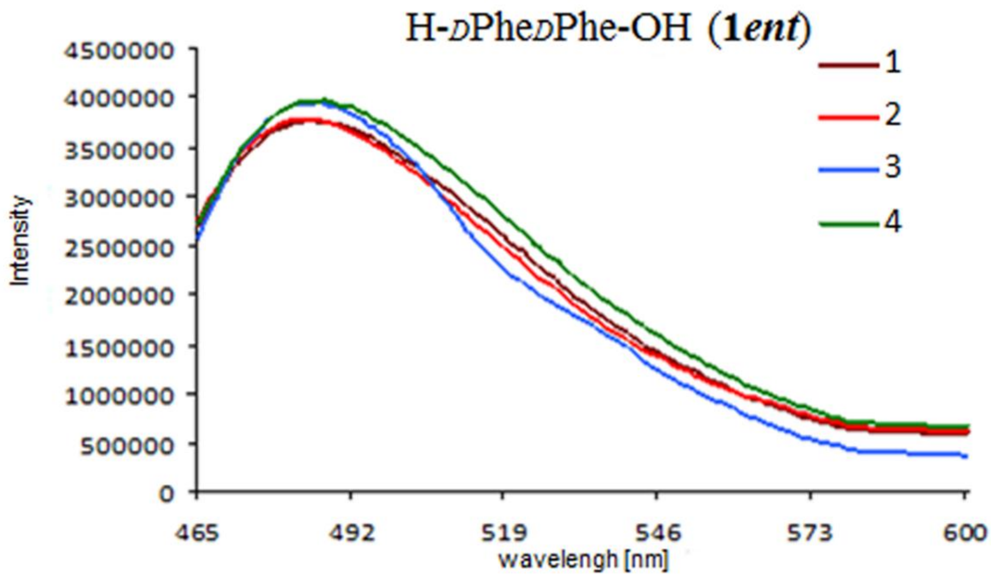

Fig. S8. Fluorescence spectra of H-DPheDPhe-OH (**1-*ent***), incubation with ThT.

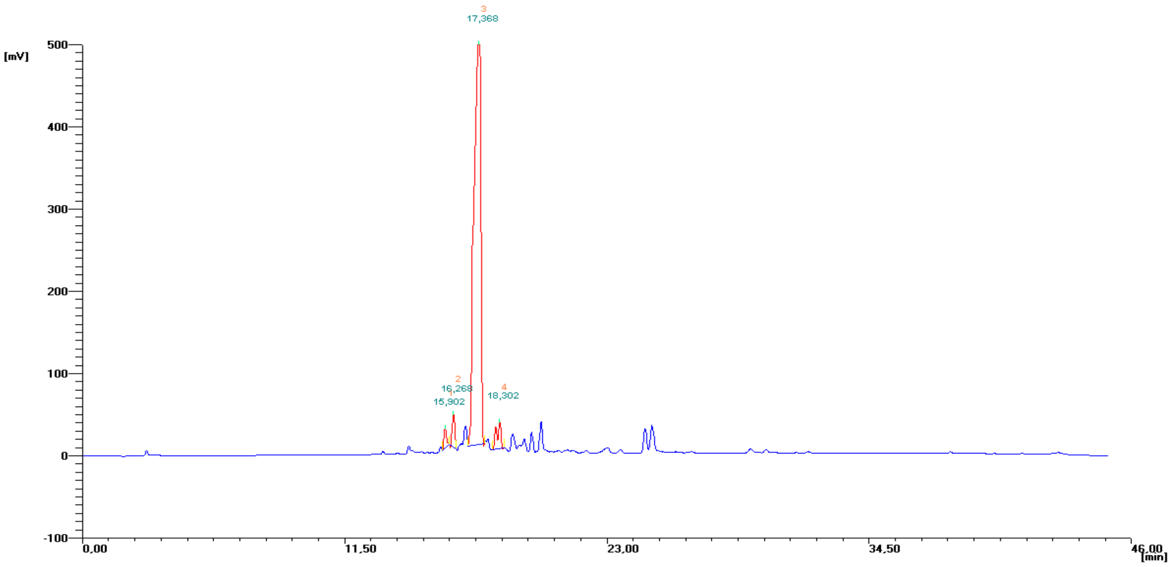

Fig. S9. HPLC spectrum of H-TrpTrp-OH (**2**).

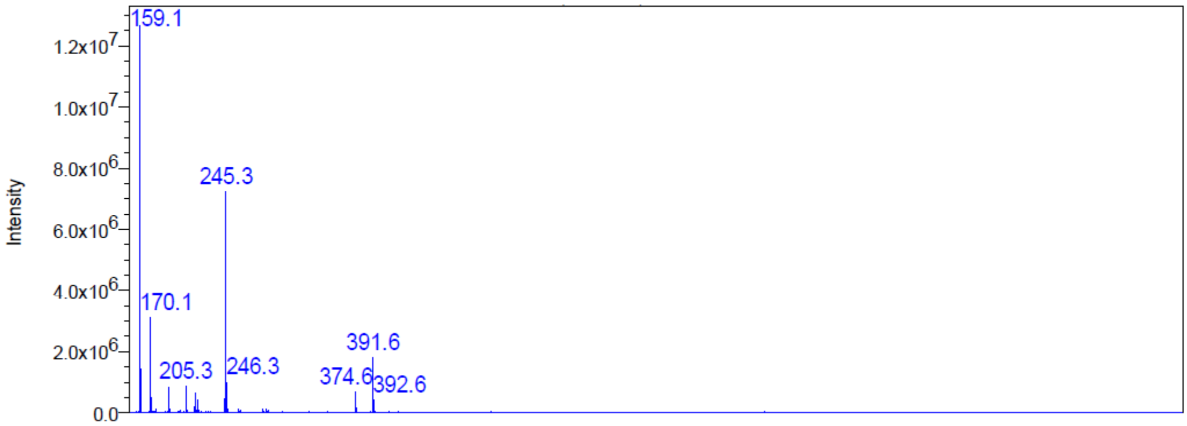

Fig. S10. MS spectrum of H-Trp-Trp-OH (**2**).

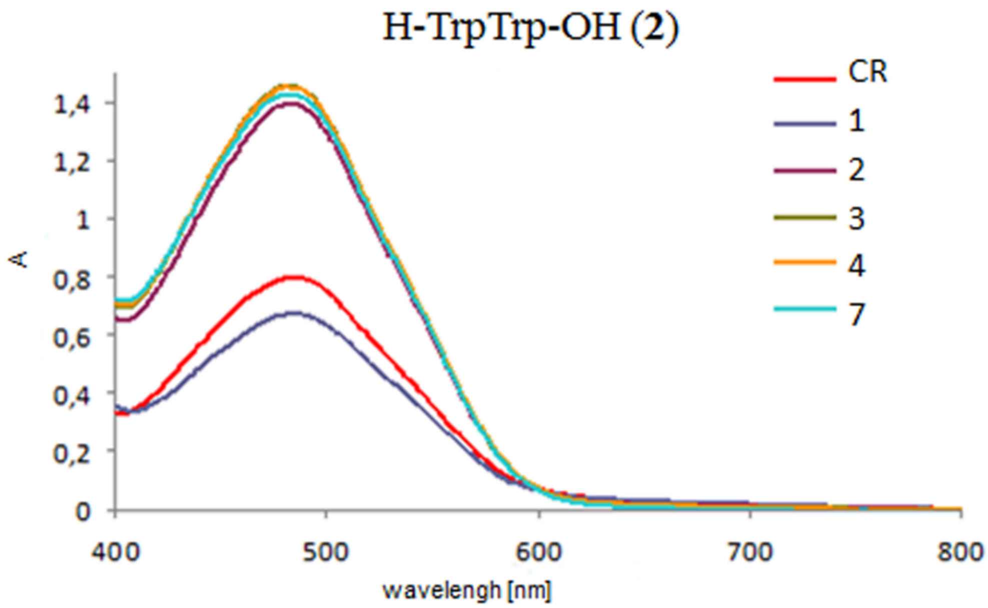

Fig. S11. UV-Vis spectra of H-TrpTrp-OH (**2**), incubation with CR.

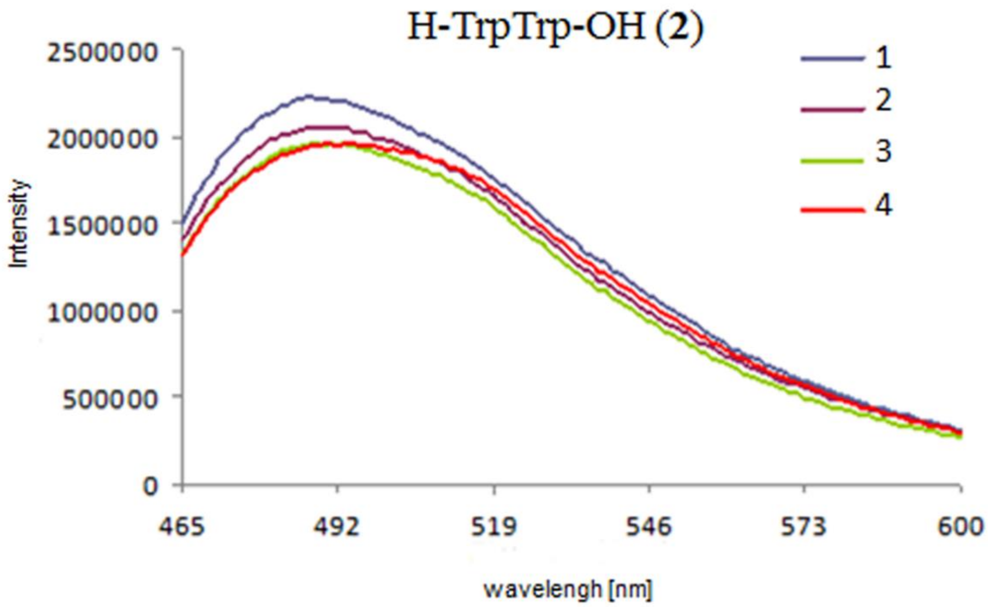

Fig. S12. Fluorescence spectra of H-TrpTrp-OH (**2**), incubation with ThT.

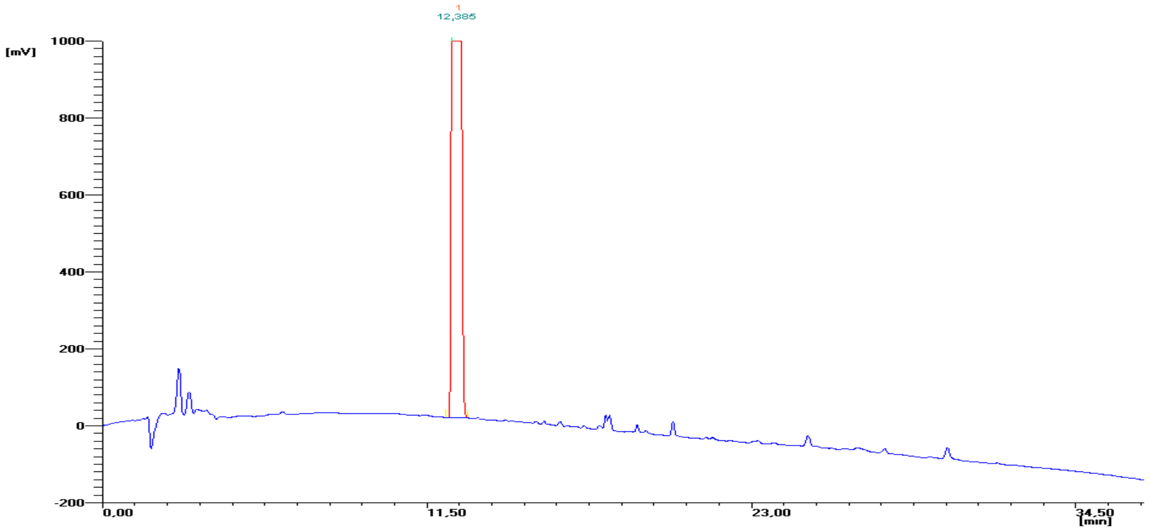

Fig. S13. HPLC spectrum of H-TyrTyr-OH (**3**).

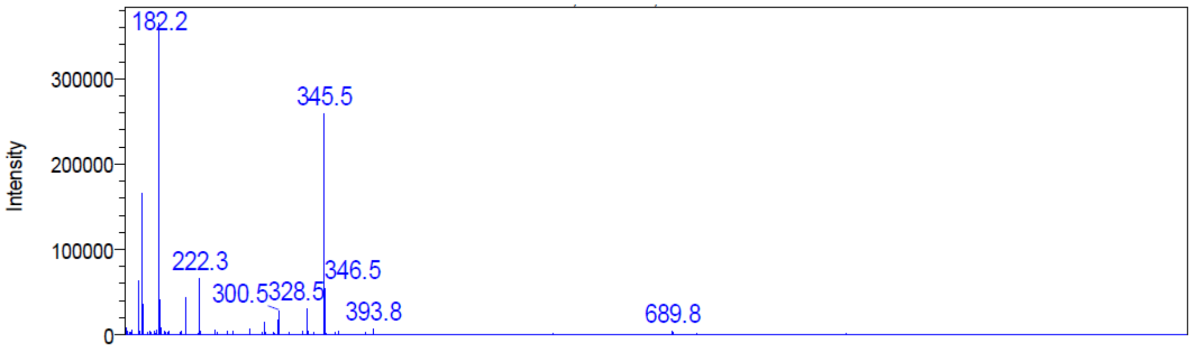

Fig. S14. MS spectrum of H-Tyr-Tyr-OH (**3**).

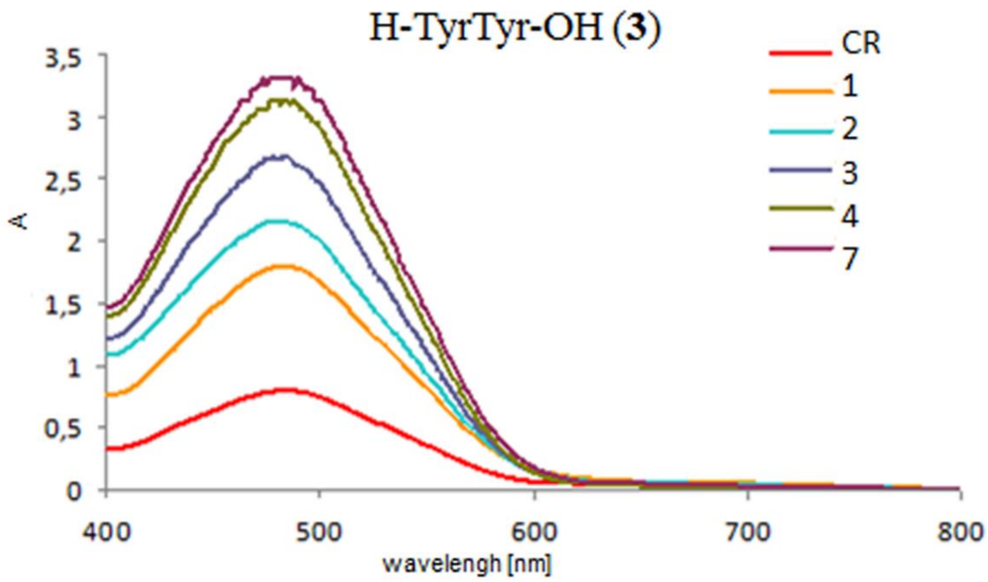

Fig. S15. UV-Vis spectra of H-TyrTyr-OH (**3**), incubation with CR.

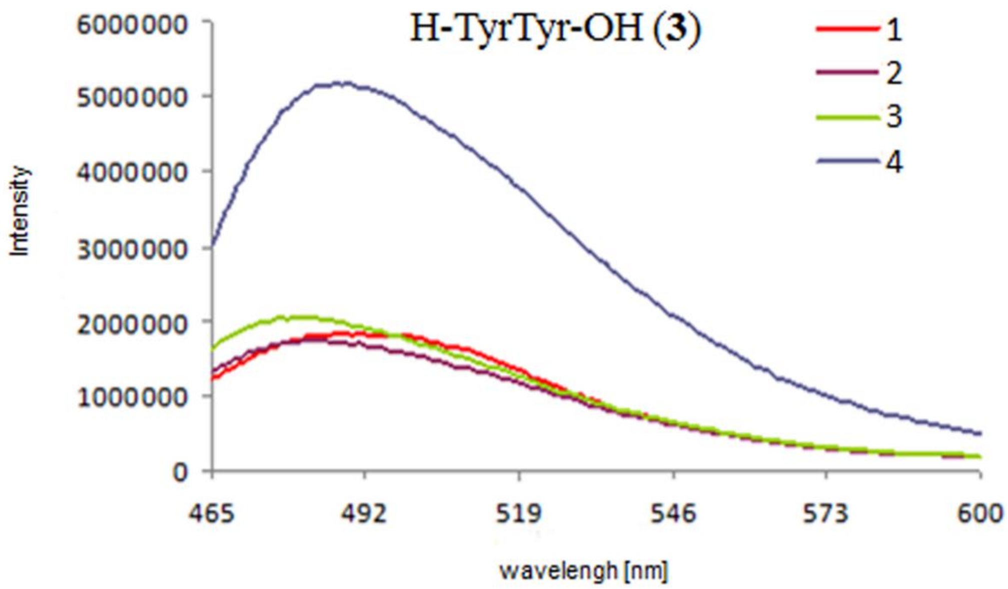

Fig. S16. Fluorescence spectra of H-TyrTyr-OH (**3**), incubation with ThT.

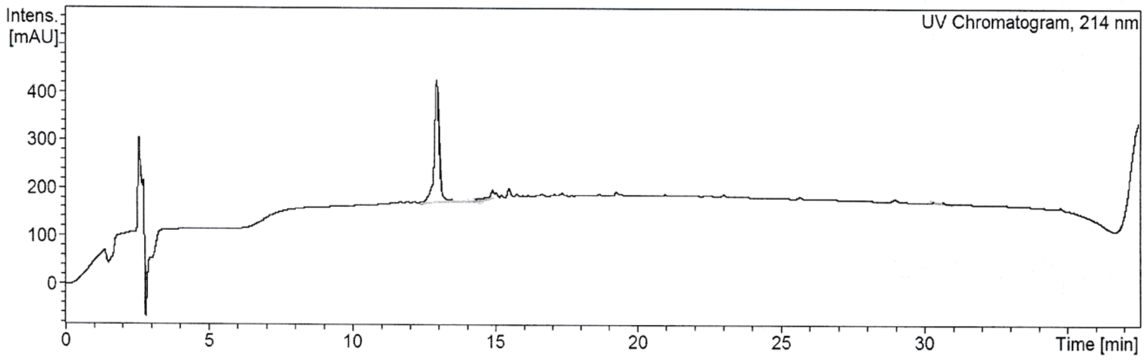

Fig. S17. HPLC spectrum of H-*D*Cys*D*Phe*D*Phe-OH (**4-*ent***).

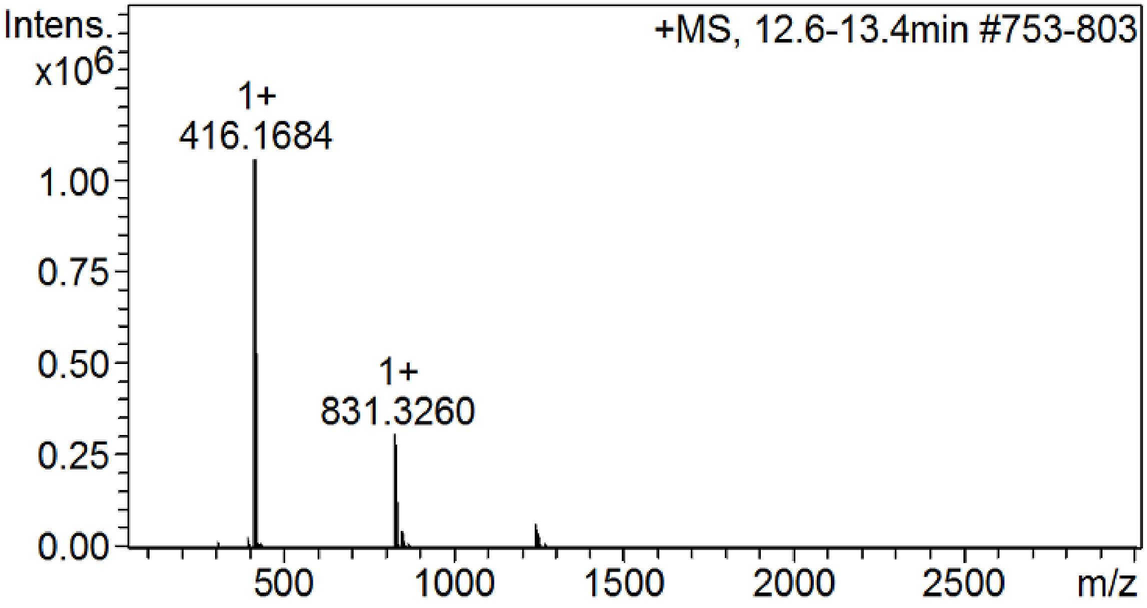

Fig. S18. MS spectrum of H-*D*Cys*D*Phe*D*Phe-OH (**4-*ent***).

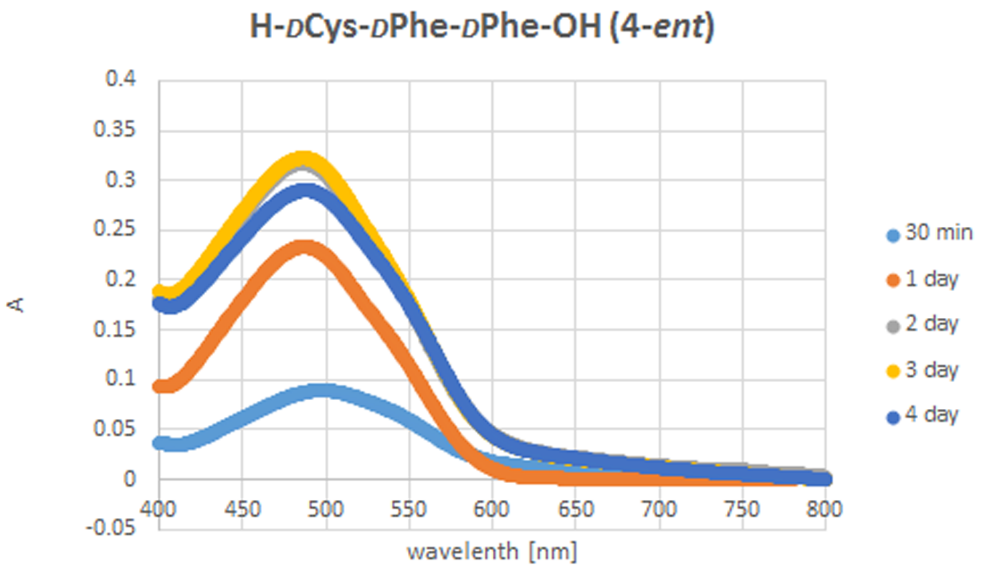

Fig. S19. UV-Vis spectra of H-DCysDPheDPhe-OH (**4-*ent***), incubation with CR.

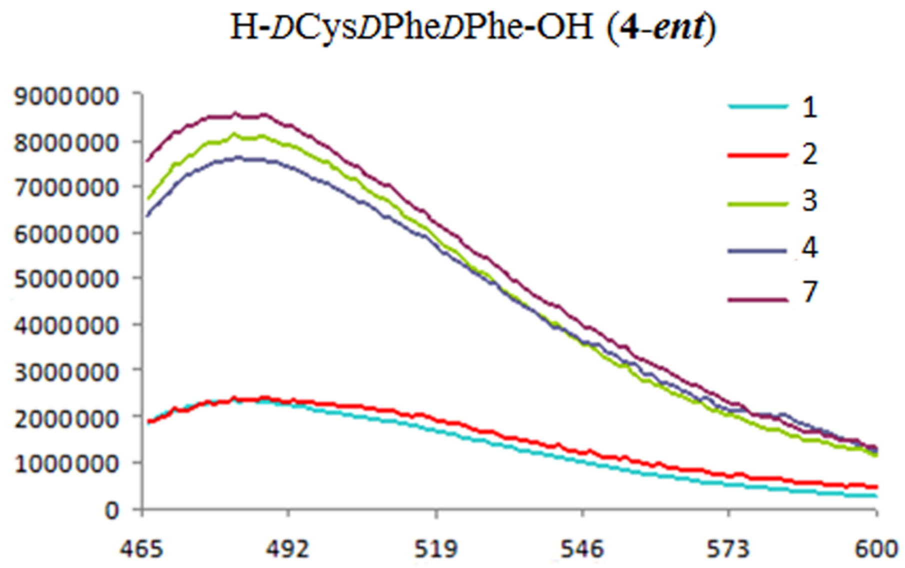

Fig. S20. Fluorescence spectra of H-DCysDPheDPhe-OH (**4-*ent***), incubation with ThT.

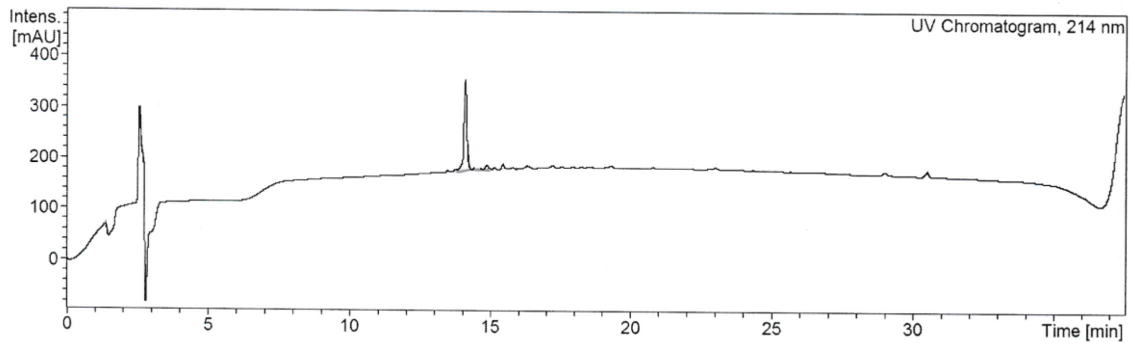

Fig. S21. HPLC spectrum of H-CysTrpTrp-OH (**5**).

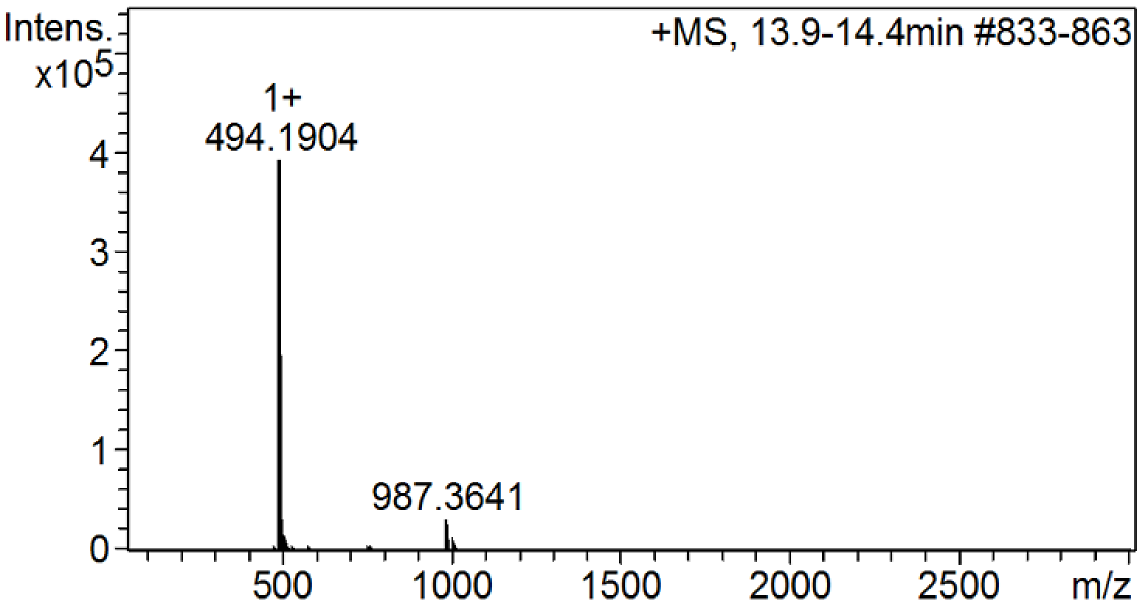

Fig. S22. MS spectrum of H-CysTrpTrp-OH (**5**).

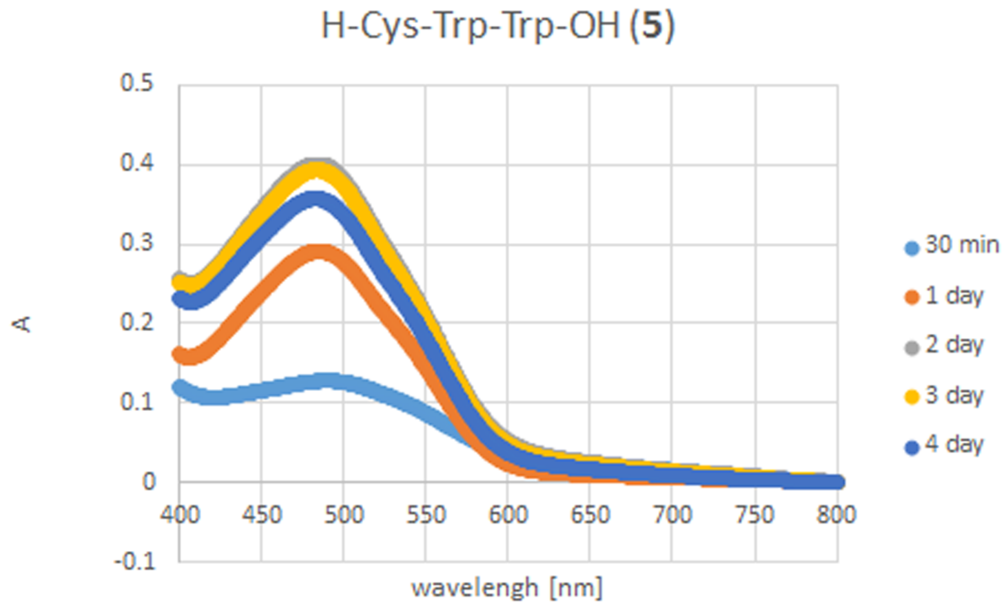

Fig. S23. UV-Vis spectra of H-CysTrpTrp-OH (**5**), incubation with CR.

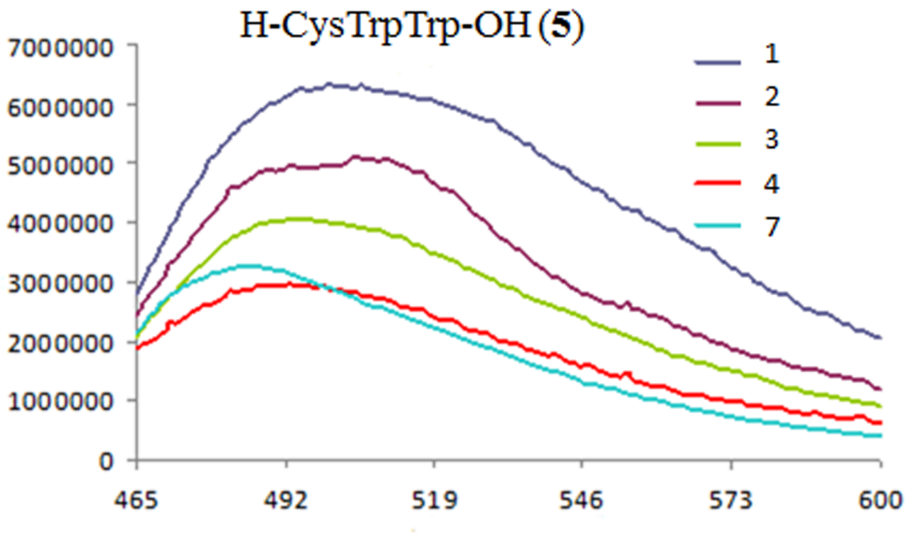

Fig. S24. Fluorescence spectra of H-CysTrpTrp-OH (**5**), incubation with ThT.

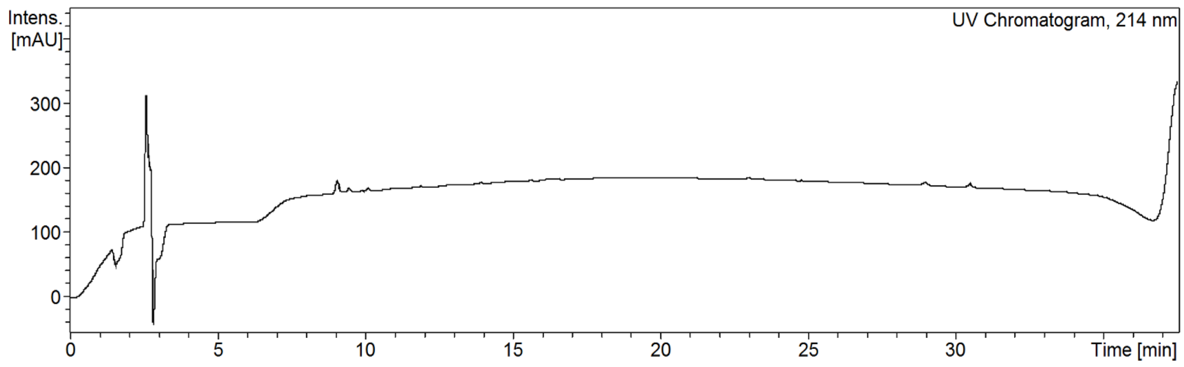

Fig. S25. HPLC spectrum of H-CysTyrTyr-OH (**6**).

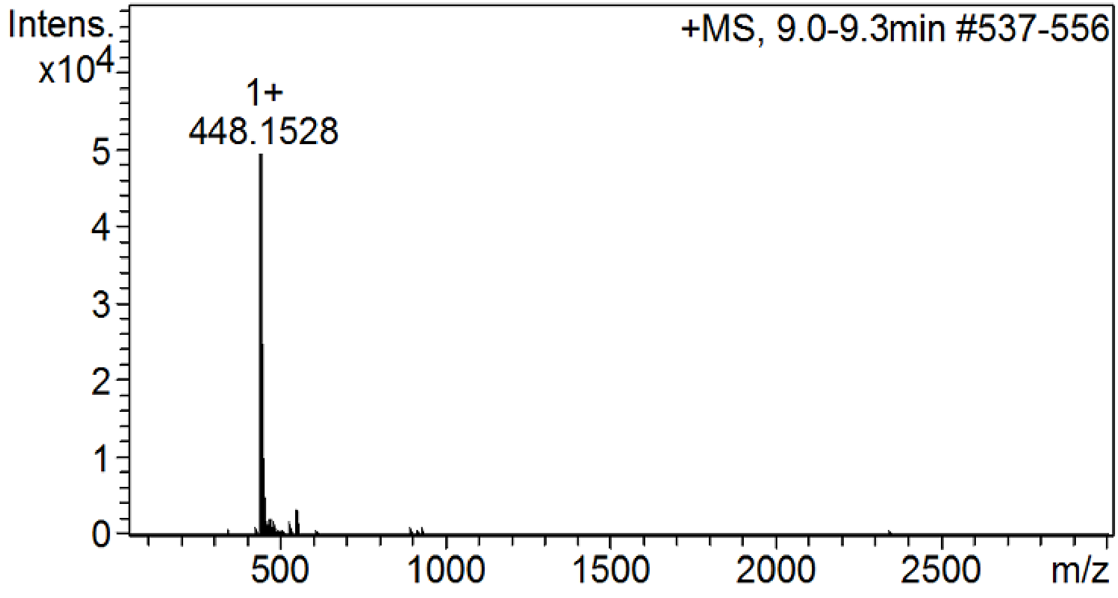

Fig. S26. MS spectrum of H-CysTyrTyr-OH (**6**).

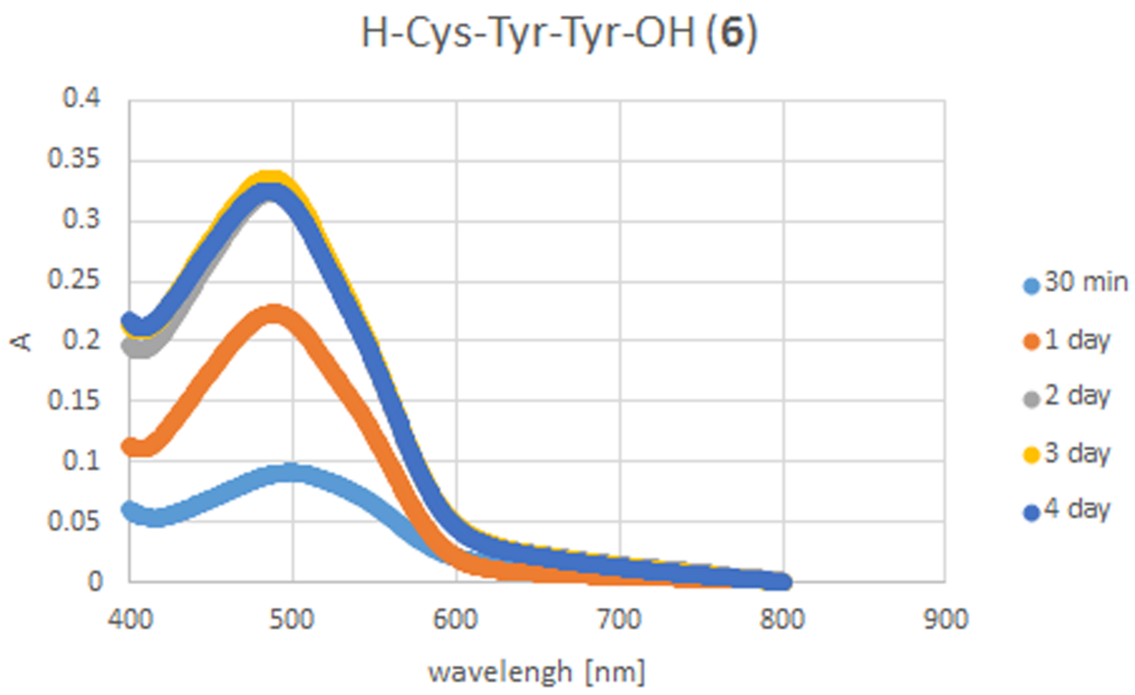

Fig. S27. UV-Vis spectra of H-CysTyrTyr-OH (**6**), incubation with CR.

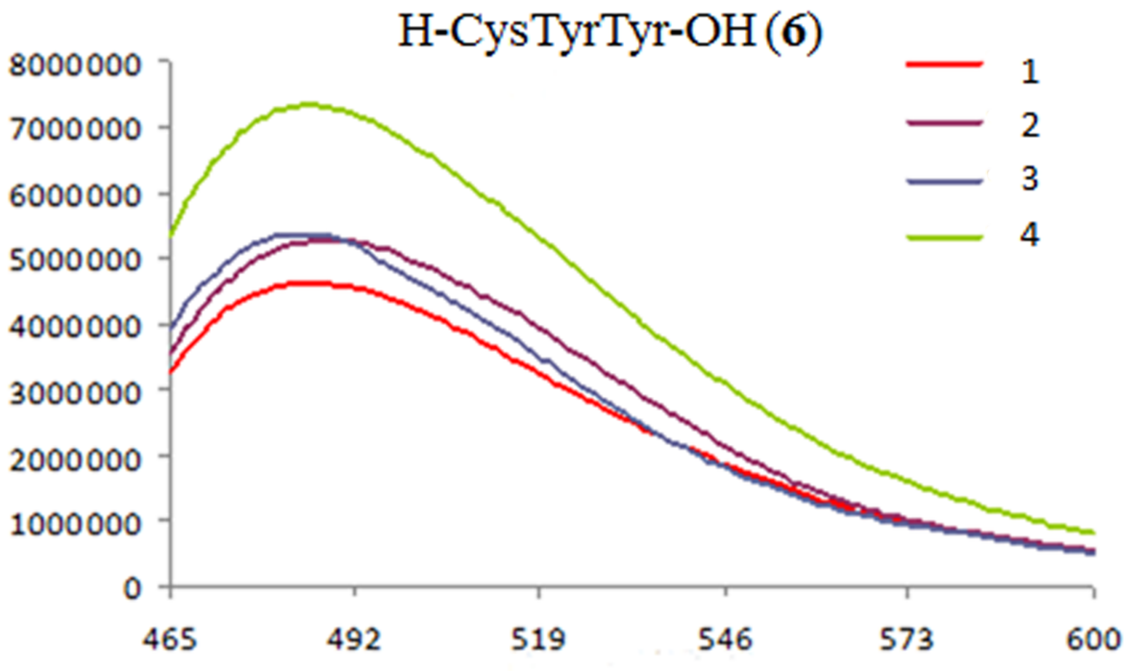

Fig. S28. Fluorescence spectra of H-CysTyrTyr-OH (**6**), incubation with ThT.

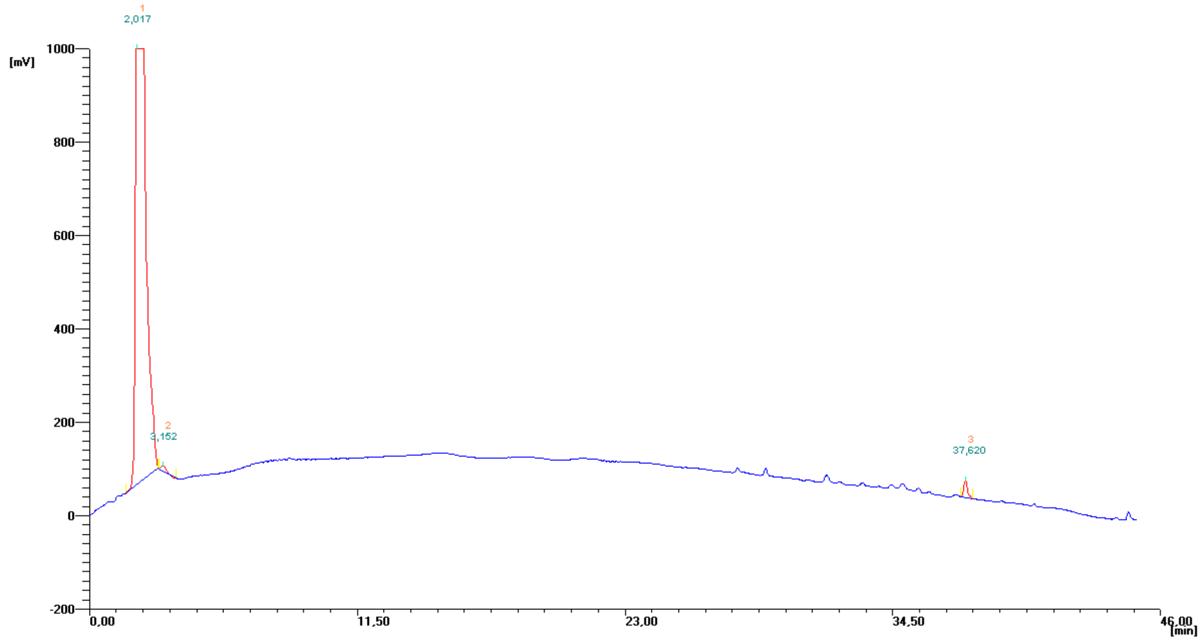

Fig. S29 HPLC spectrum of H-PhePheCys-OH (**7**).

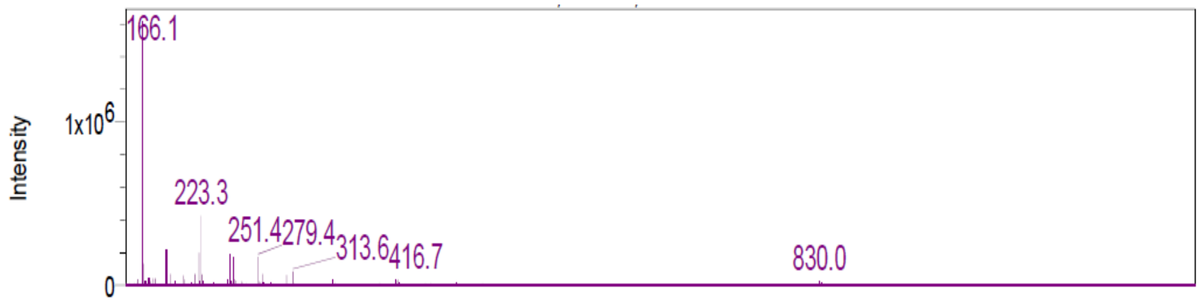

Fig. S30. MS spectrum of H-PhePheCys-OH (**7**).

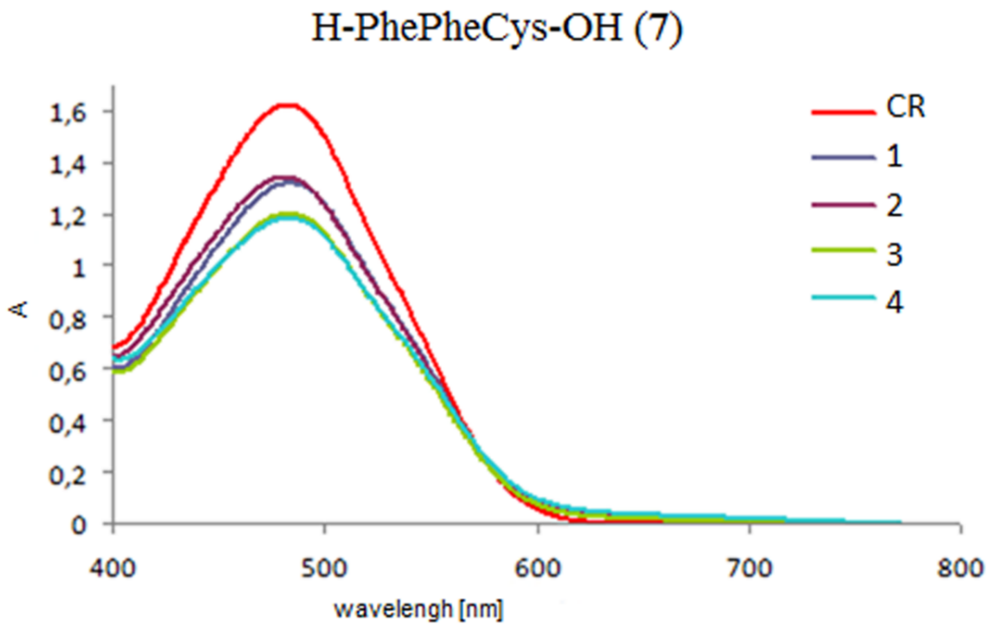

Fig. S31. UV-Vis spectra of H-PhePheCys-OH (**7**), incubation with CR.

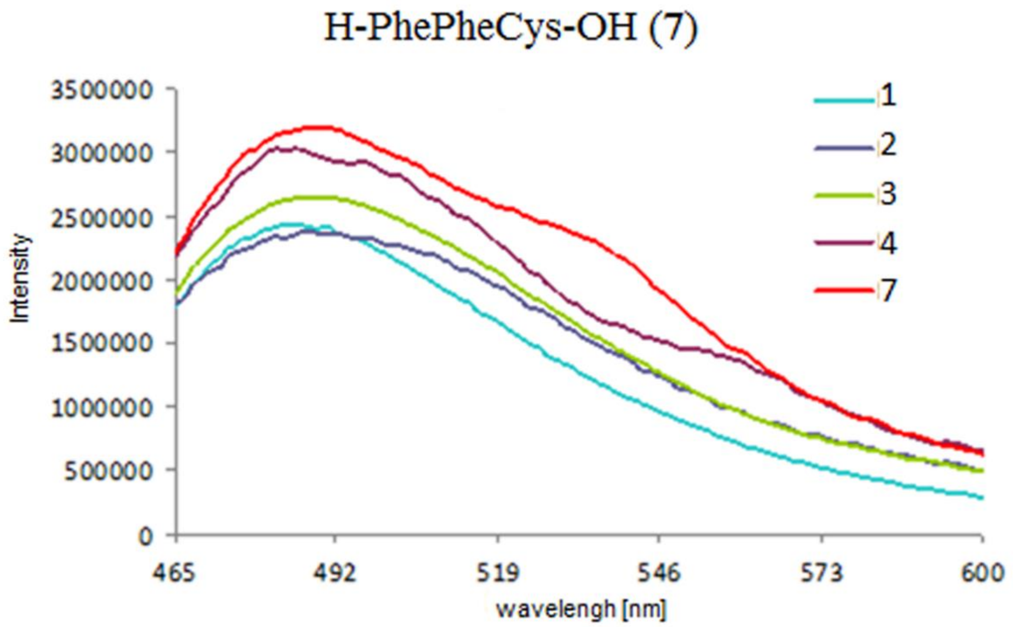

Fig. S32. Fluorescence spectra of H-PhePheCys-OH (**7**), incubation with ThT.

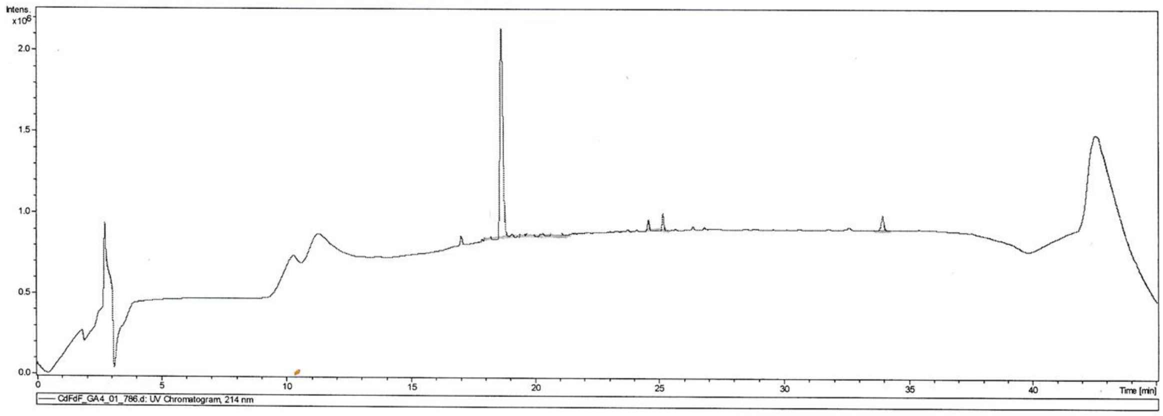

Fig. S33. HPLC spectrum of H-*D*Phe*D*Phe*D*Cys-OH (***7-ent***).

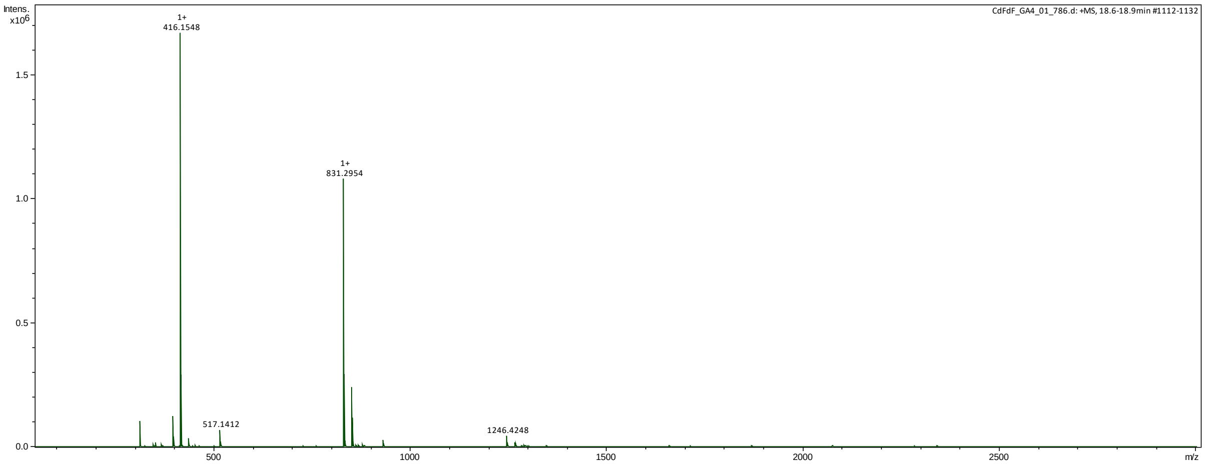

Fig. S34. MS spectrum of H-*D*Phe*D*Phe*D*Cys-OH (***7-ent***).

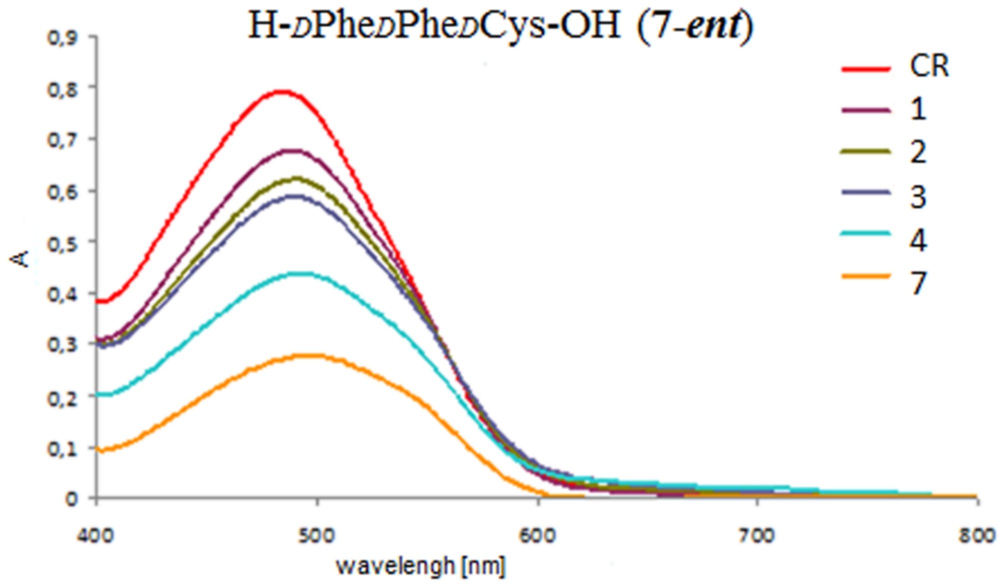

Fig. S35. UV-Vis spectra of H-*D*Phe*D*Phe*D*Cys-OH (**7-*ent***), incubation with CR.

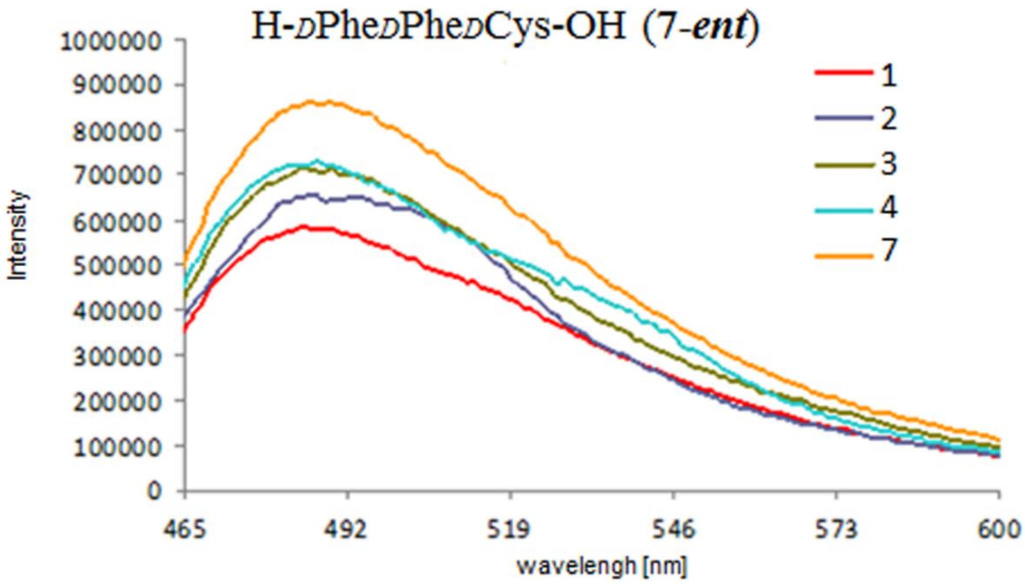

Fig. S36. Fluorescence spectra of H-*D*Phe*D*Phe*D*Cys-OH (**7-*ent***), incubation with ThT.

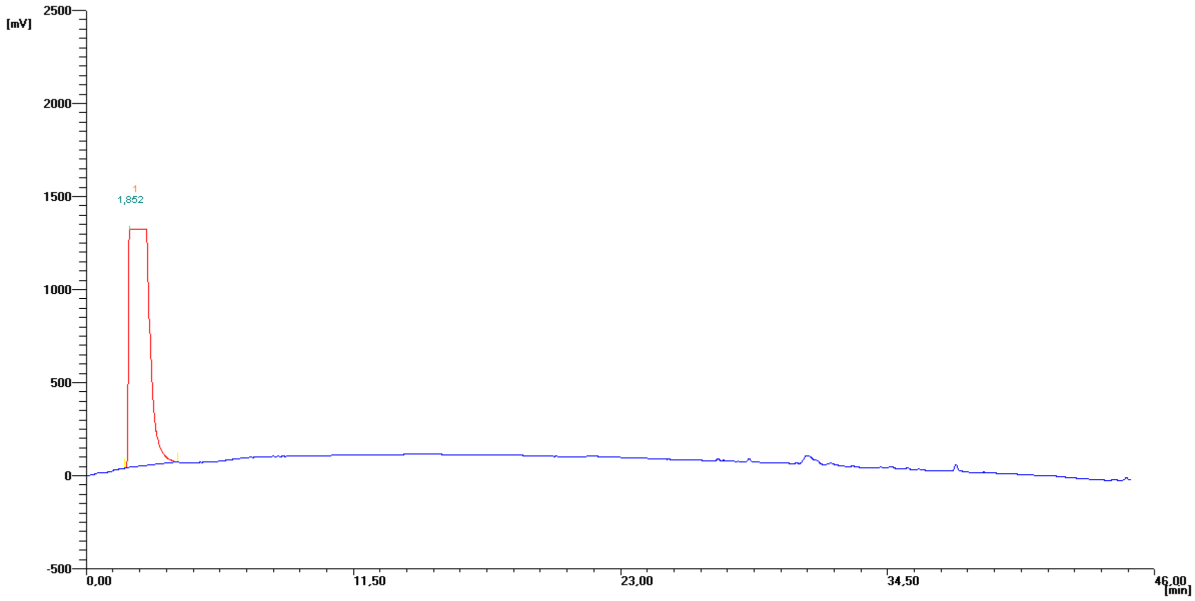

Fig. S37. HPLC spectrum of H-TrpTrpCys-OH (**8**).

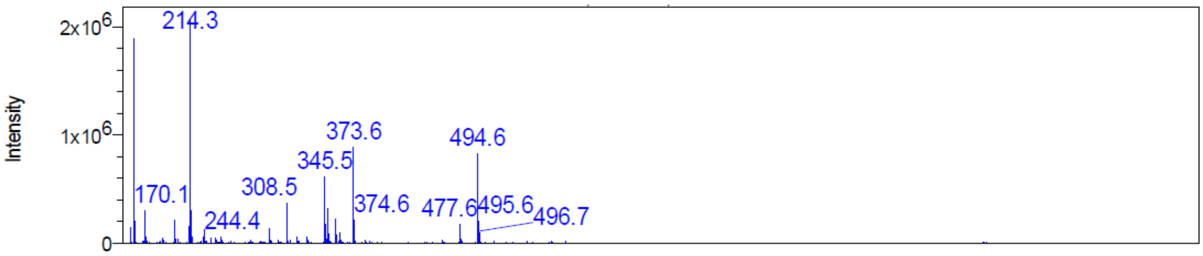

Fig. S38. MS spectrum of H-TrpTrpCys-OH (**8**).

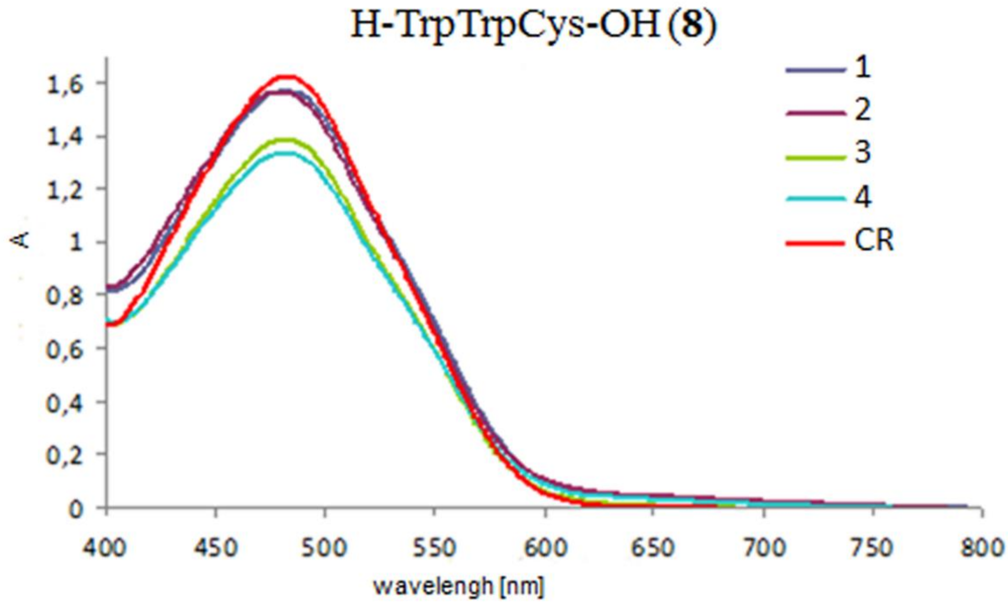

Fig. S39. UV-Vis spectra of H-TrpTrpCys-OH (**8**), incubation with CR.

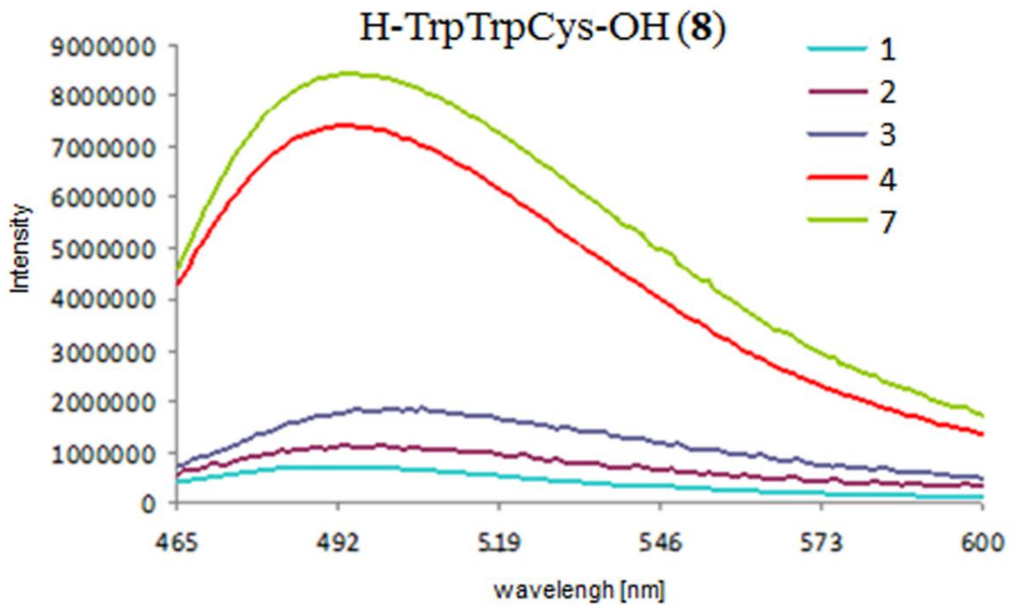

Fig. S40. Fluorescence spectra of H-TrpTrpCys-OH (**8**), incubation with ThT.

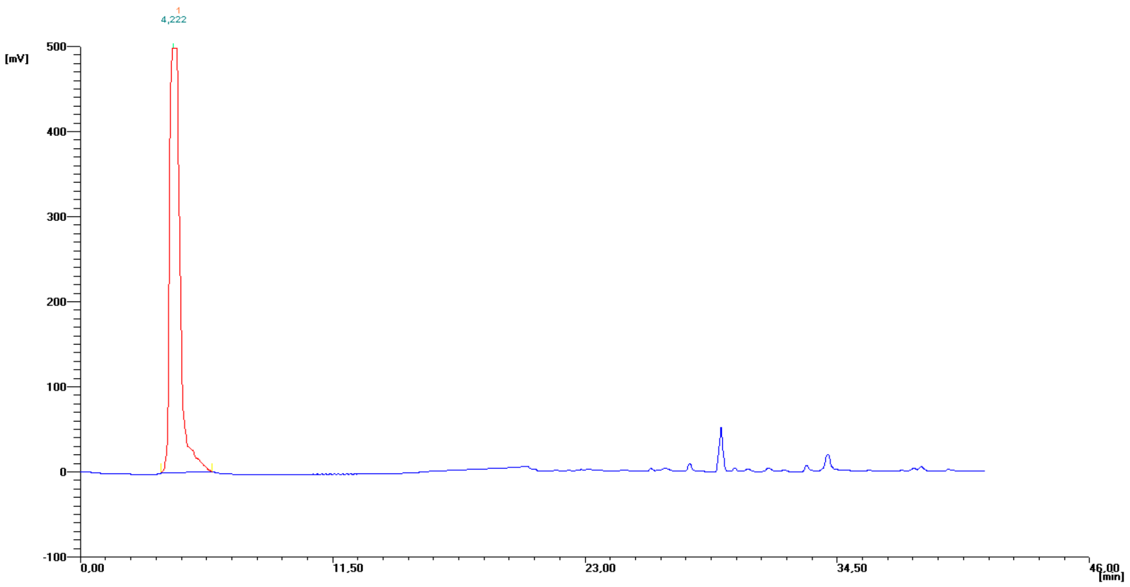

Fig. S41. HPLC spectrum of H-TyrTyrCys-OH (**9**).

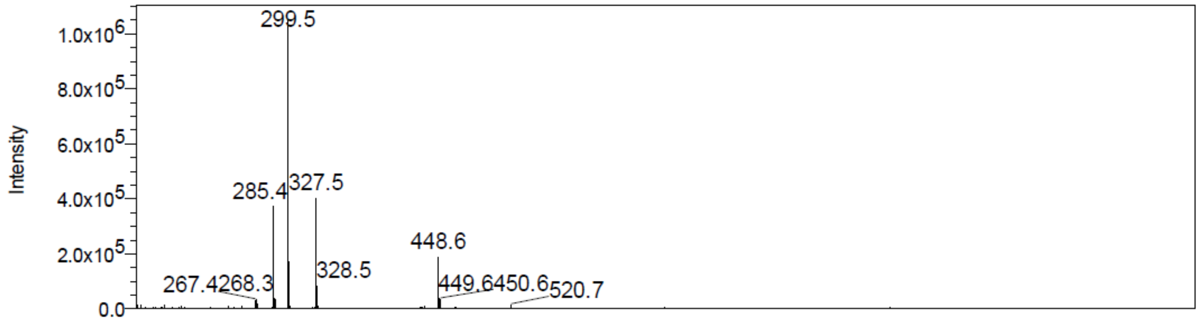

Fig. S42. MS spectrum of H-TyrTyrCys-OH (**9**).

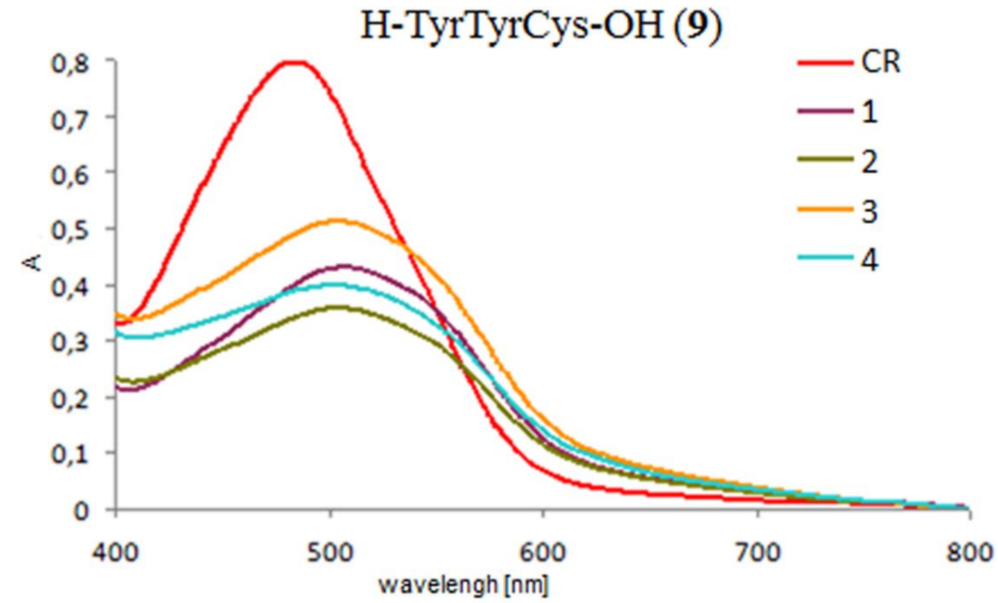

Fig. S43. UV-Vis spectra of H-TyrTyrCys-OH (**9**), incubation with CR.

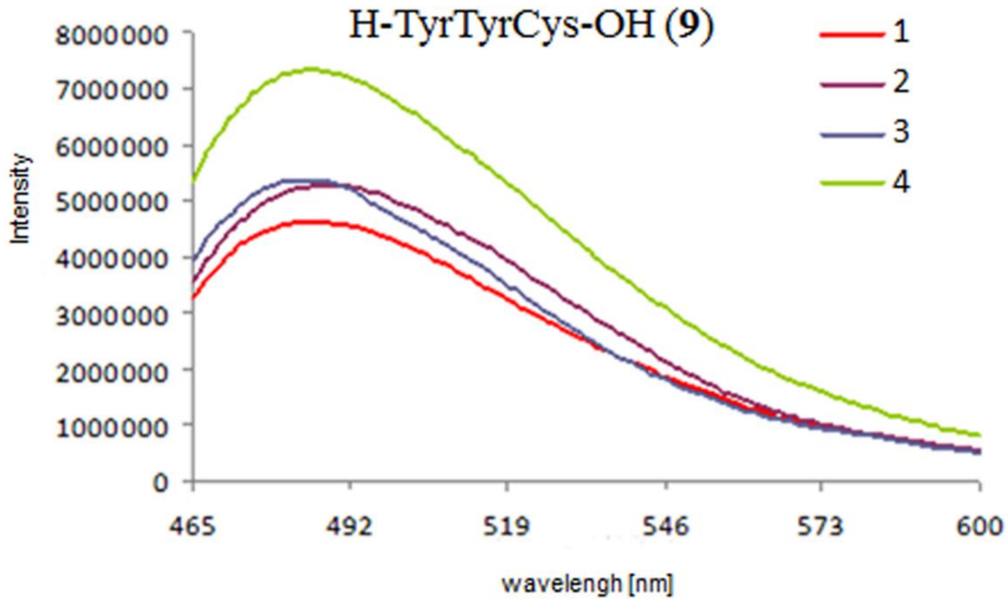

Fig. S44. Fluorescence spectra of H-TyrTyrCys-OH (**9**), incubation with ThT.

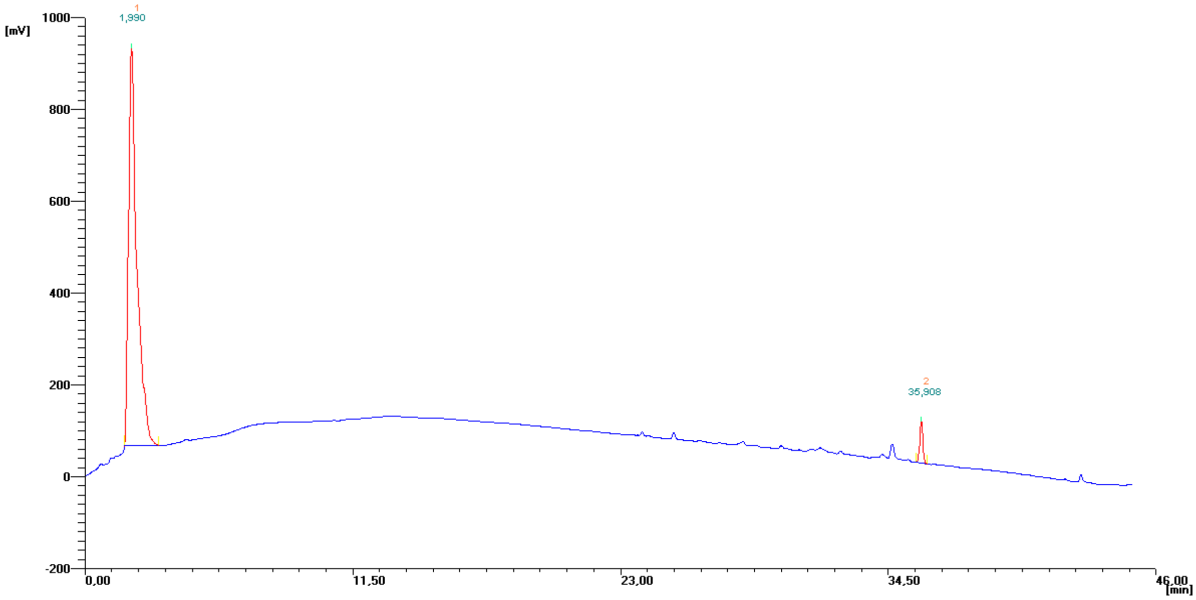

Fig. S45. HPLC spectrum of H-PheCysPhe-OH (**10**).

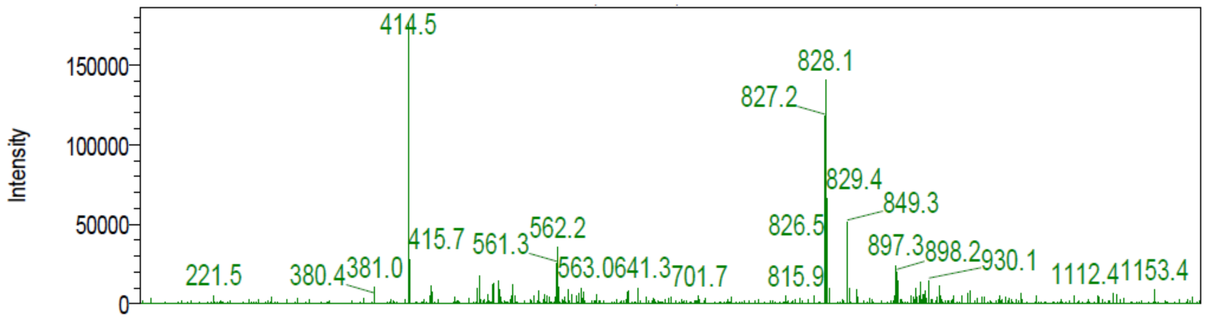

Fig. S46. MS spectrum of H-PheCysPhe-OH (**10**).

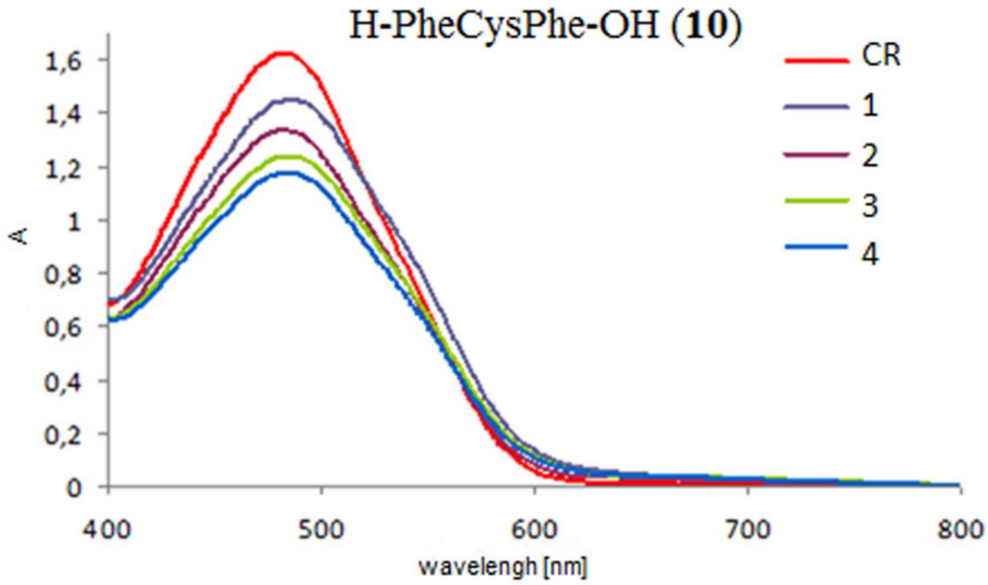

Fig. S47. UV-Vis spectra of H-PheCysPhe-OH (**10**), incubation with CR.

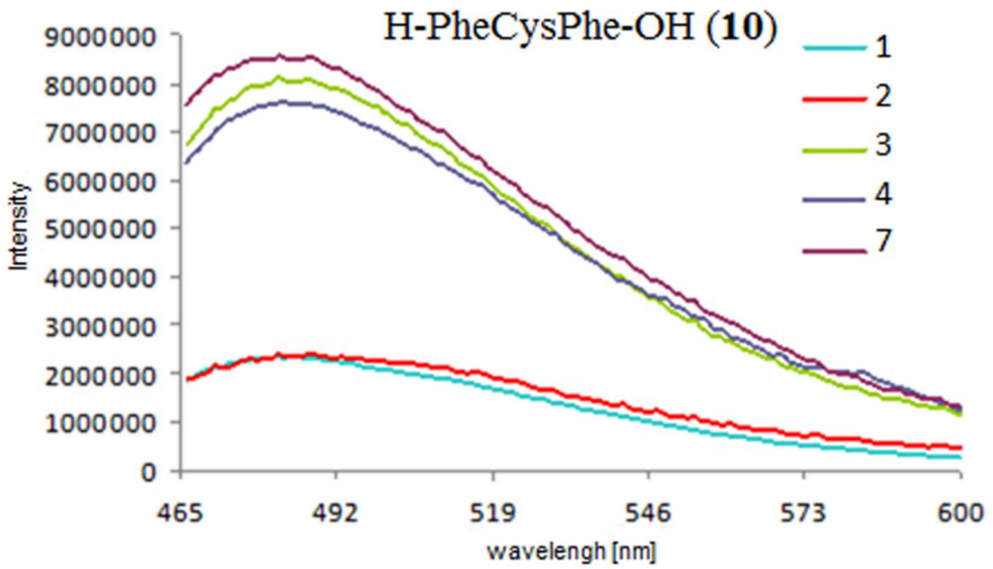

Fig. S48. Fluorescence spectra of H-PheCysPhe-OH (**10**), incubation with ThT.

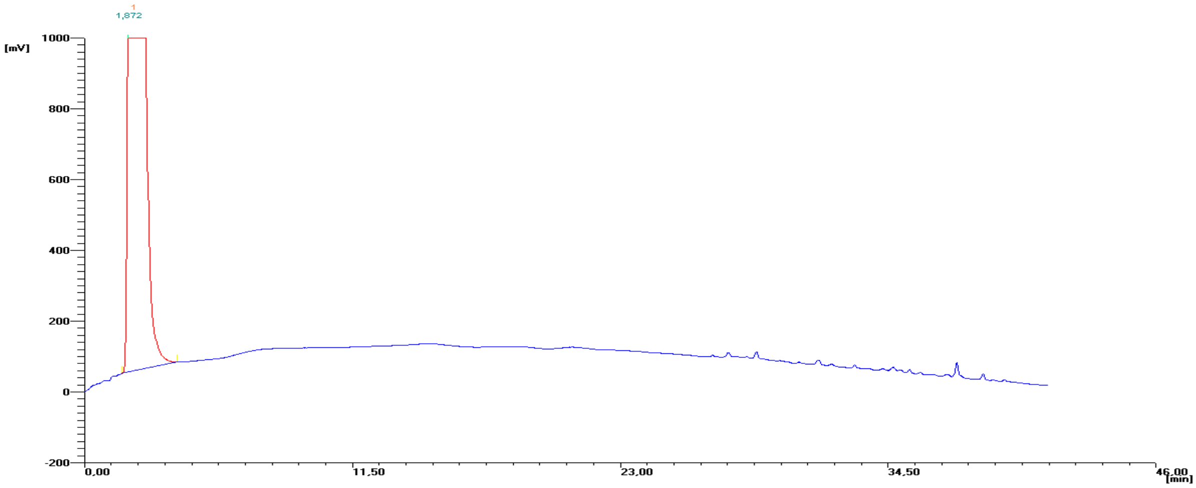

Fig. S49. HPLC spectrum of H-TrpCysTrp-OH (**11**).

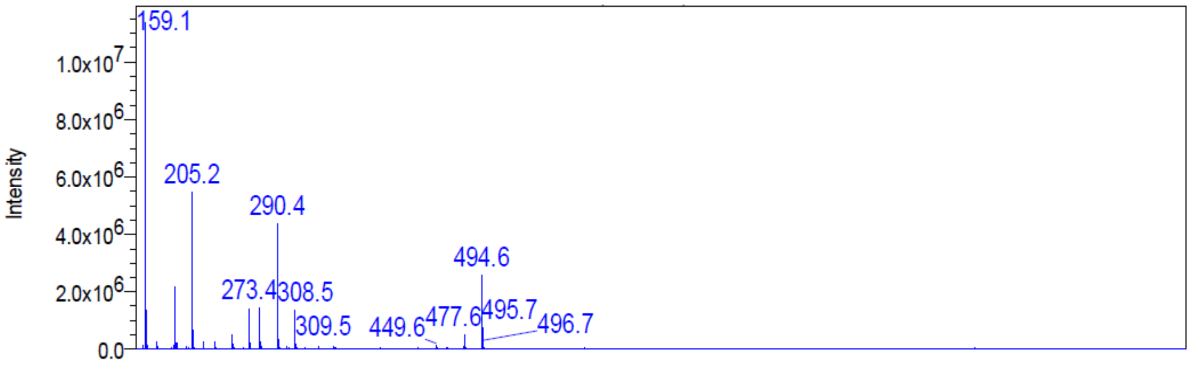

Fig. S50. MS spectrum of H-TrpCysTrp-OH (**11**).

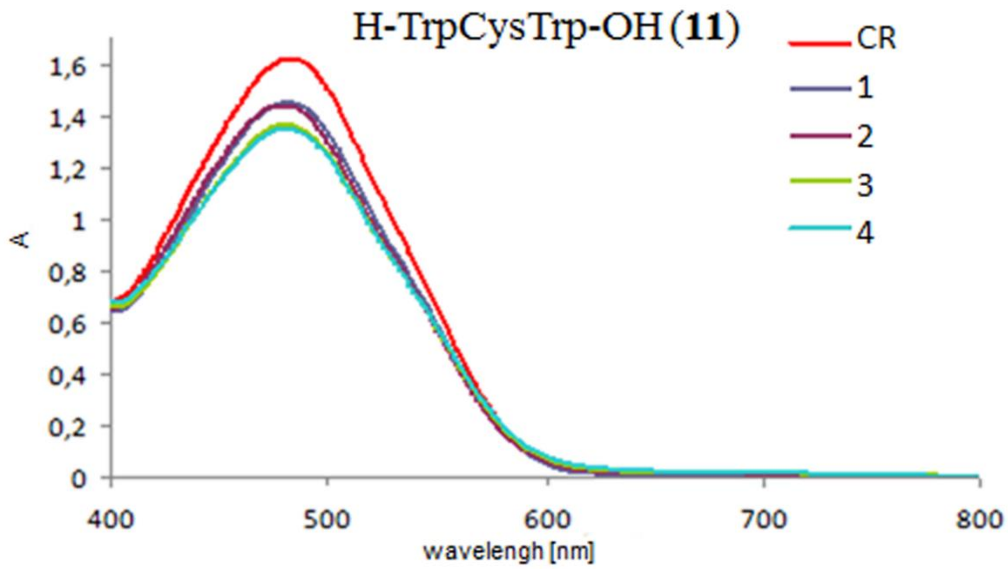

Fig. S51. UV-Vis spectra of H-TrpCysTrp-OH (**11**), incubation with CR.

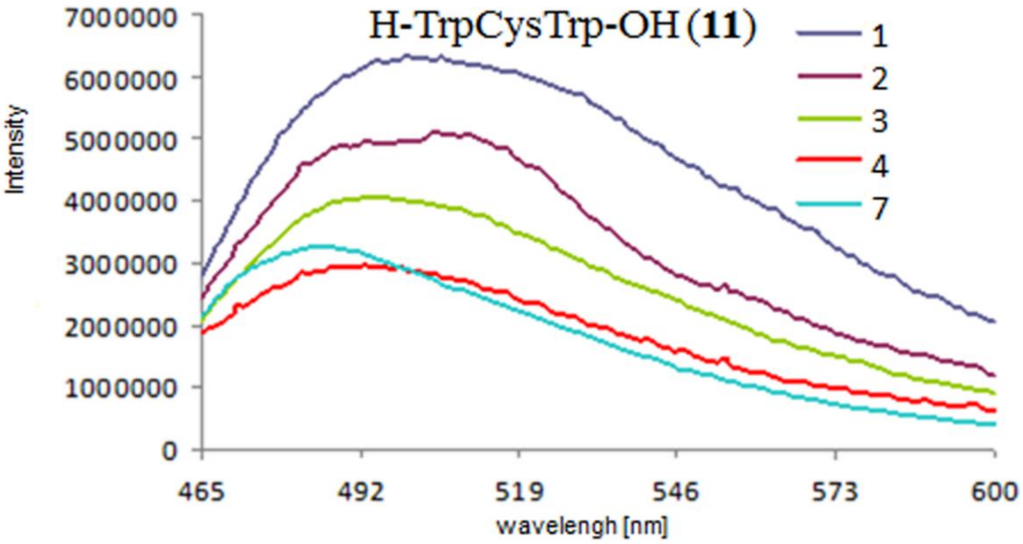

Fig. S52. Fluorescence spectra of H-TrpCysTrp-OH (**11**), incubation with ThT.

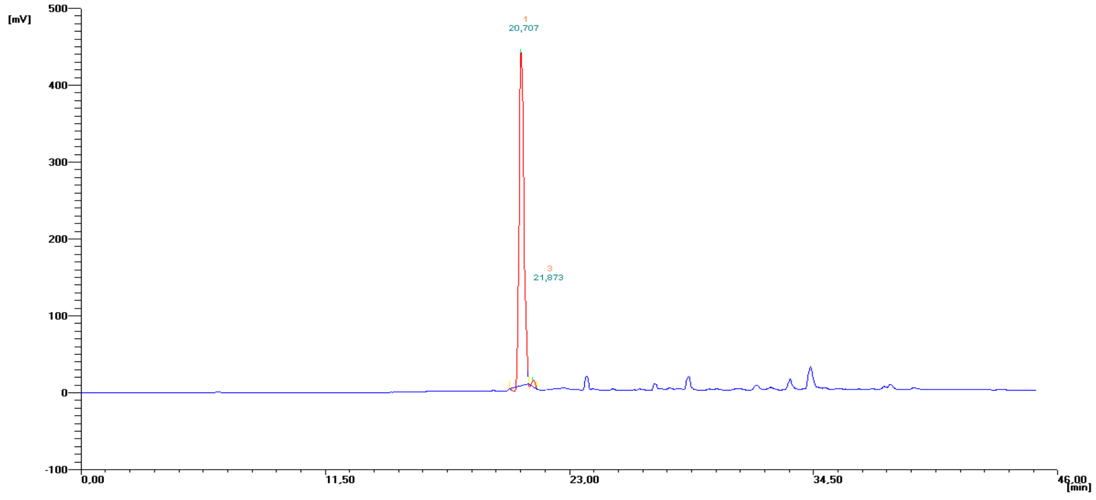

Fig. S53. HPLC spectrum of H-TyrCysTyr-OH (**12**).

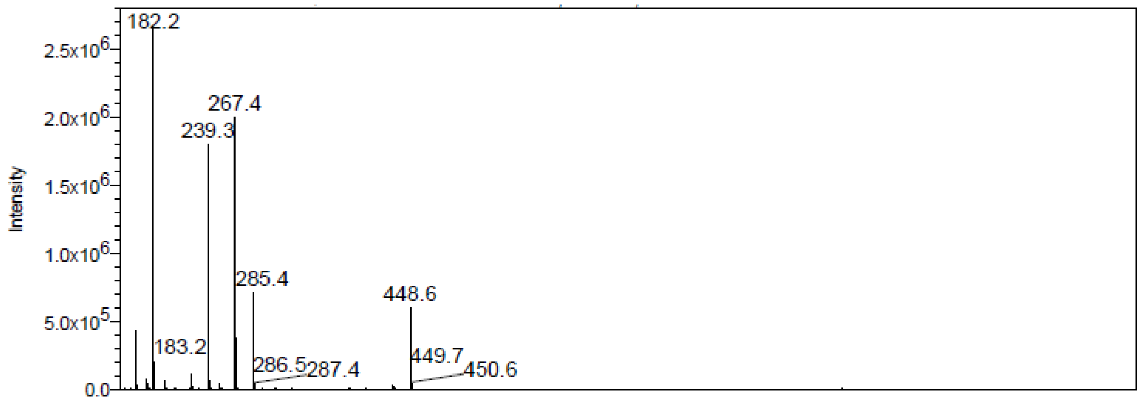

Fig. S54. MS spectrum of H-TyrCysTyr-OH (**12**).

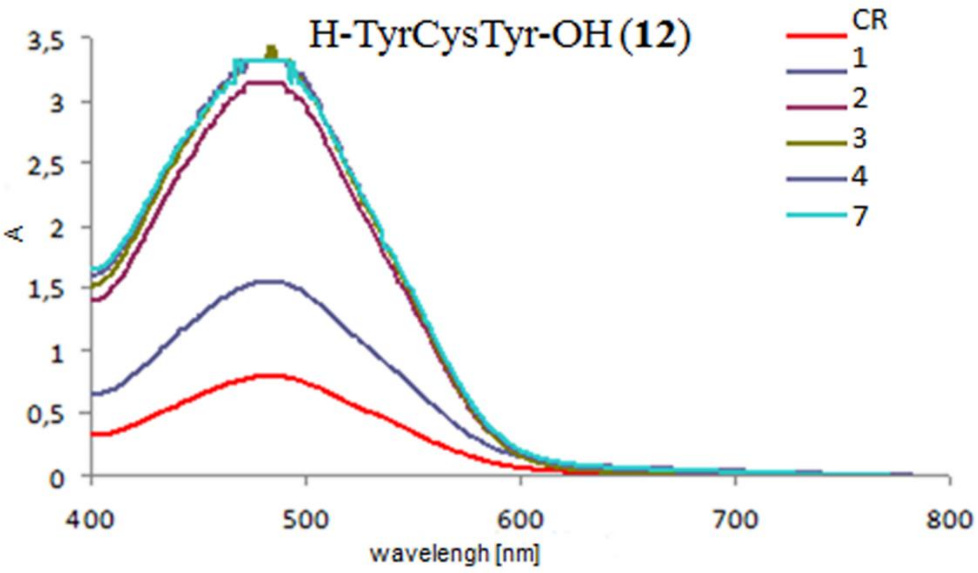

Fig. S55. UV-Vis spectra of H-TyrCysTyr-OH (**12**), incubation with CR.

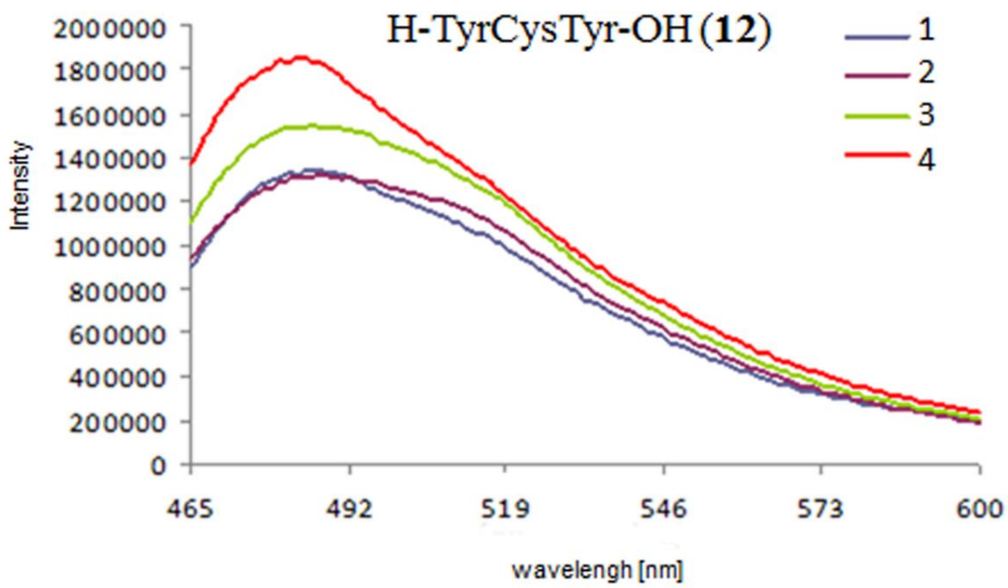

Fig. S56. Fluorescence spectra of H-TyrCysTyr-OH (**12**), incubation with ThT.

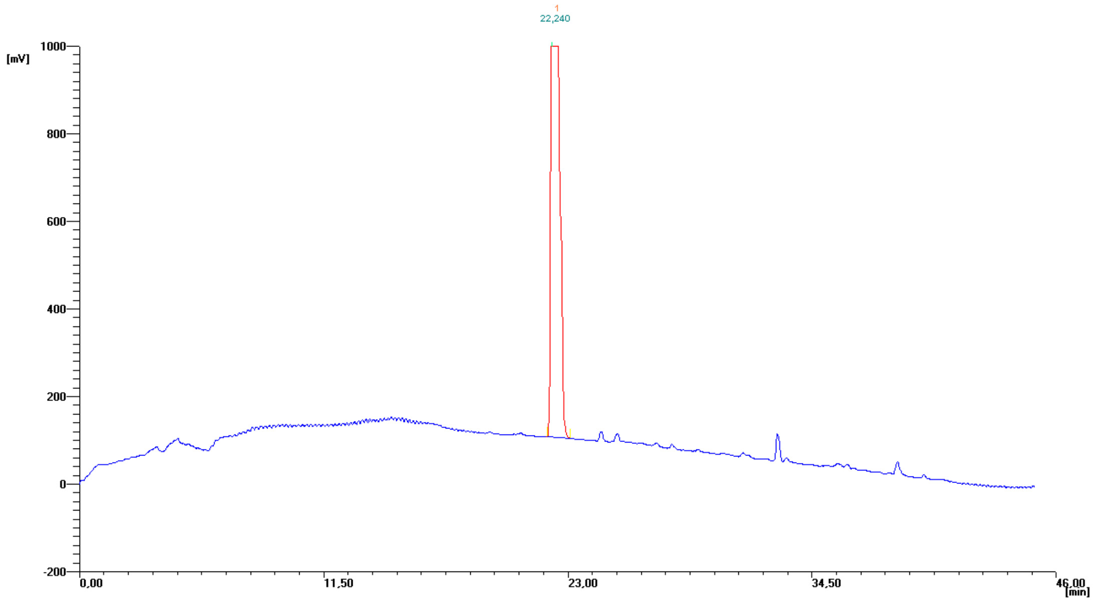

Fig. S57. HPLC spectrum of H-PhePheMet-OH (**13**).

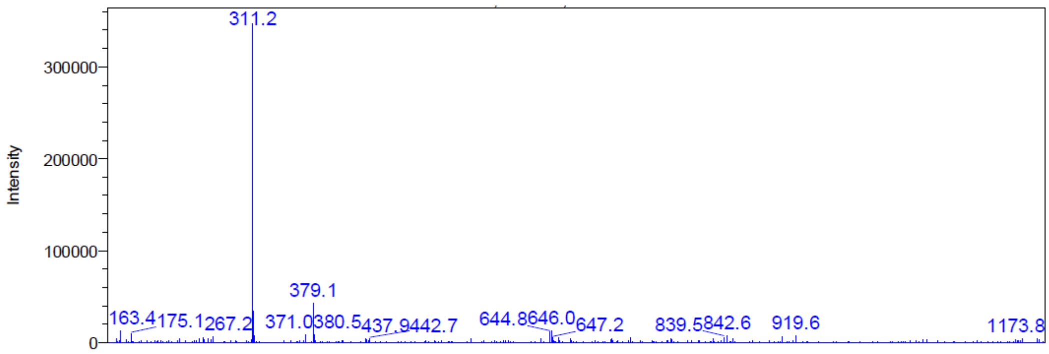

Fig. S58. MS spectrum of H-PhePheMet-OH (**13**).

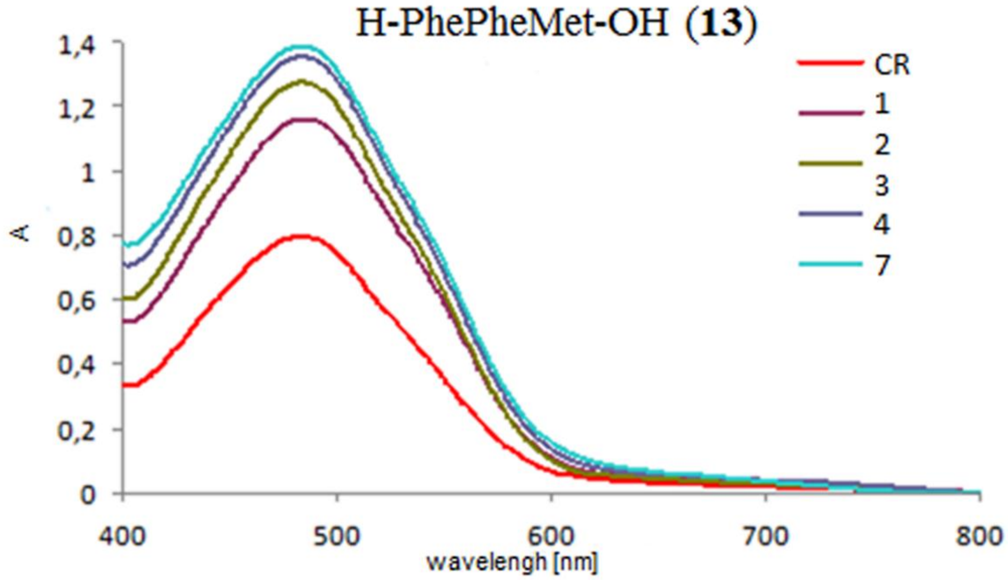

Fig. S59. UV-Vis spectra of H-PhePheMet-OH (**13**), incubation with CR.

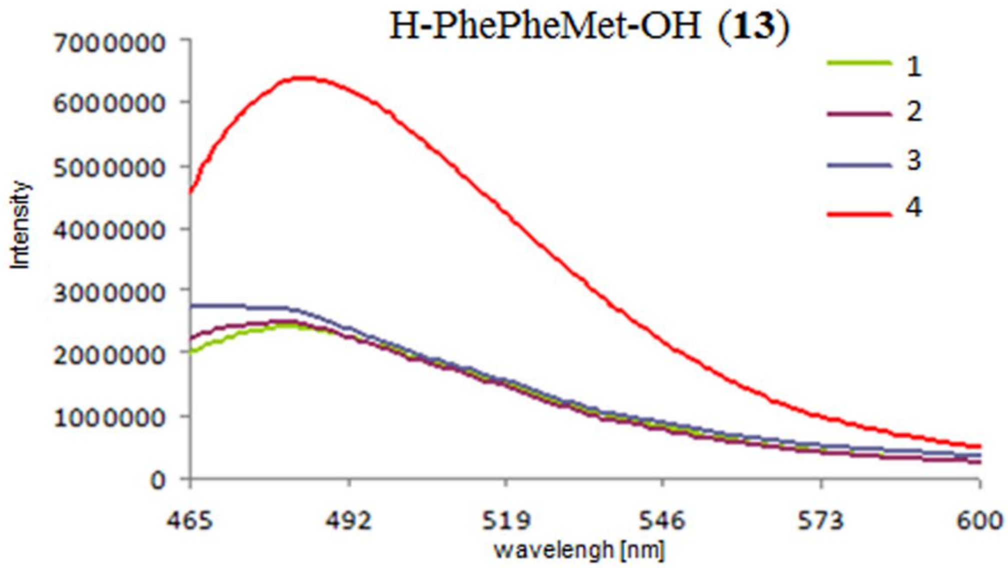

Fig. S60. Fluorescence spectra of H-PhePheMet-OH (**13**), incubation with ThT.

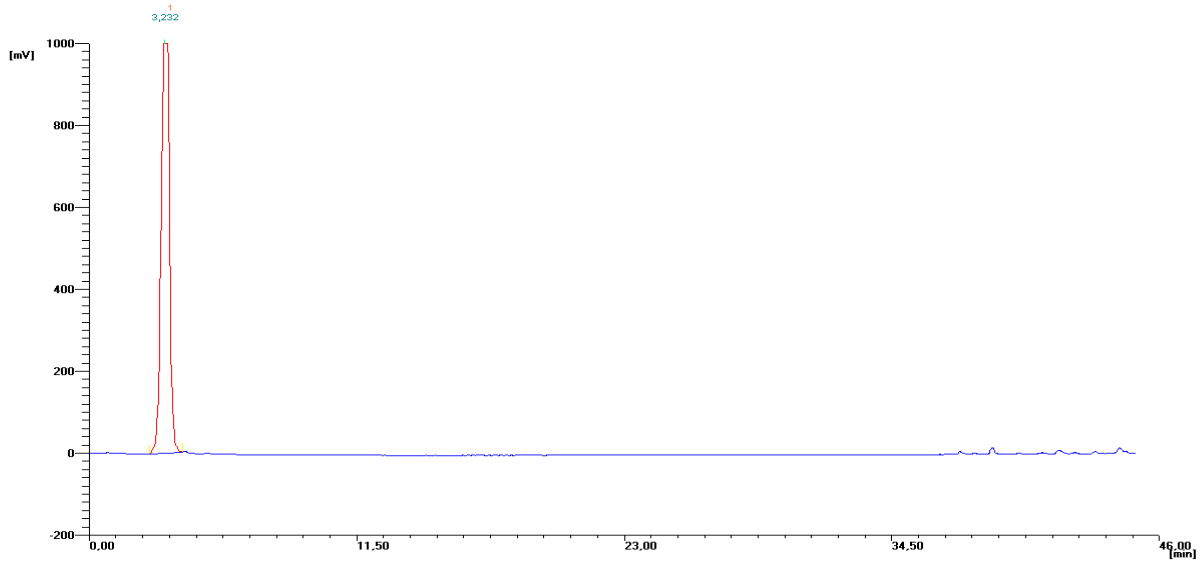

Fig. S61. HPLC spectrum of H-TrpTrpMet-OH (**14**).

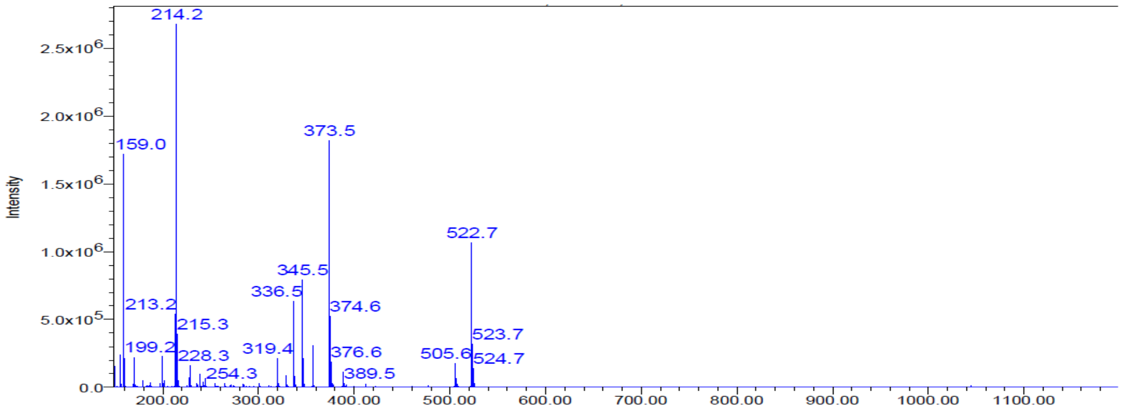

Fig. S62. MS spectrum of H-TrpTrpMet-OH (**14**).

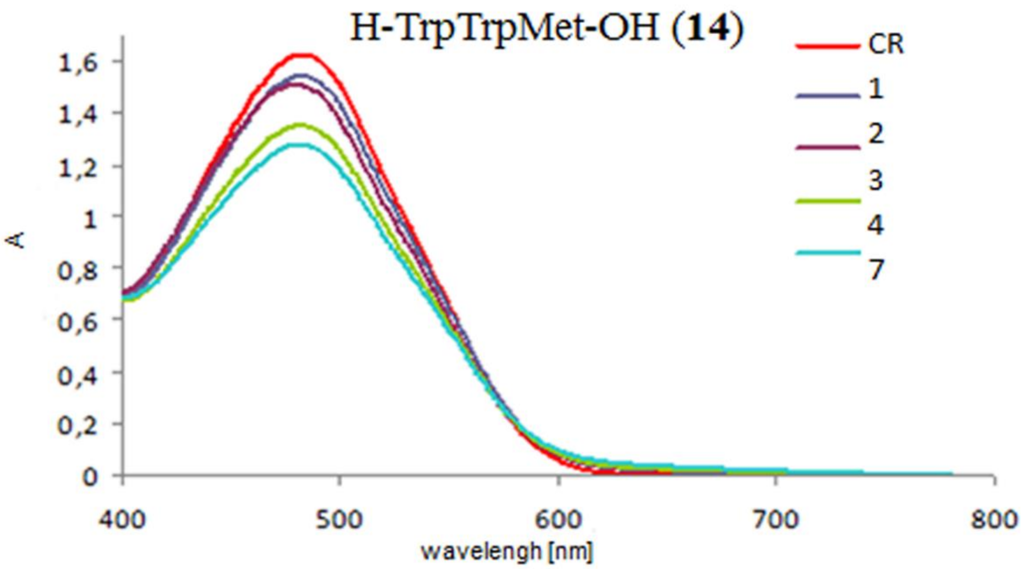

Fig. S63. UV-Vis spectra of H-TrpTrpMet-OH (**14**), incubation with CR.

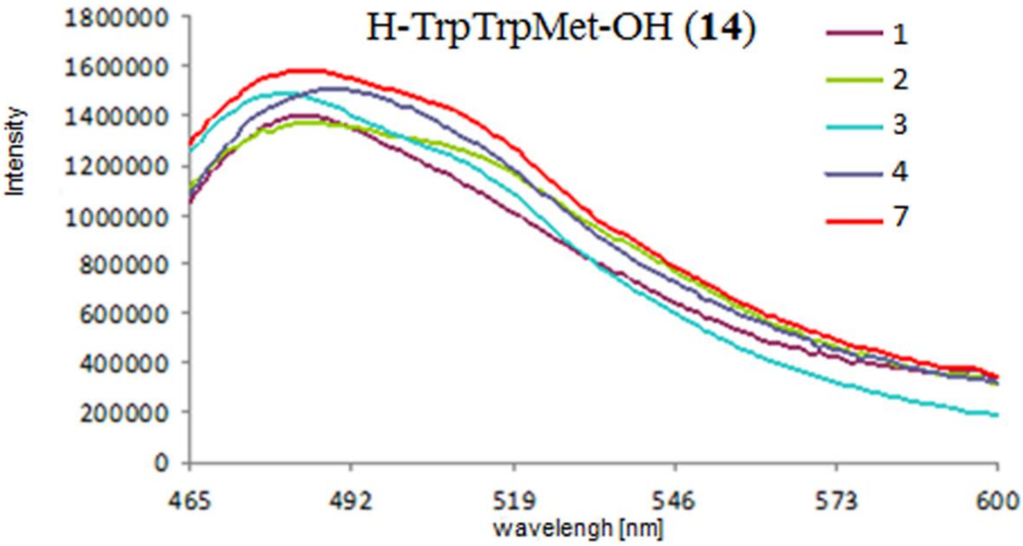

Fig. S64. Fluorescence spectra of H-TrpTrpMet-OH (**14**), incubation with ThT.

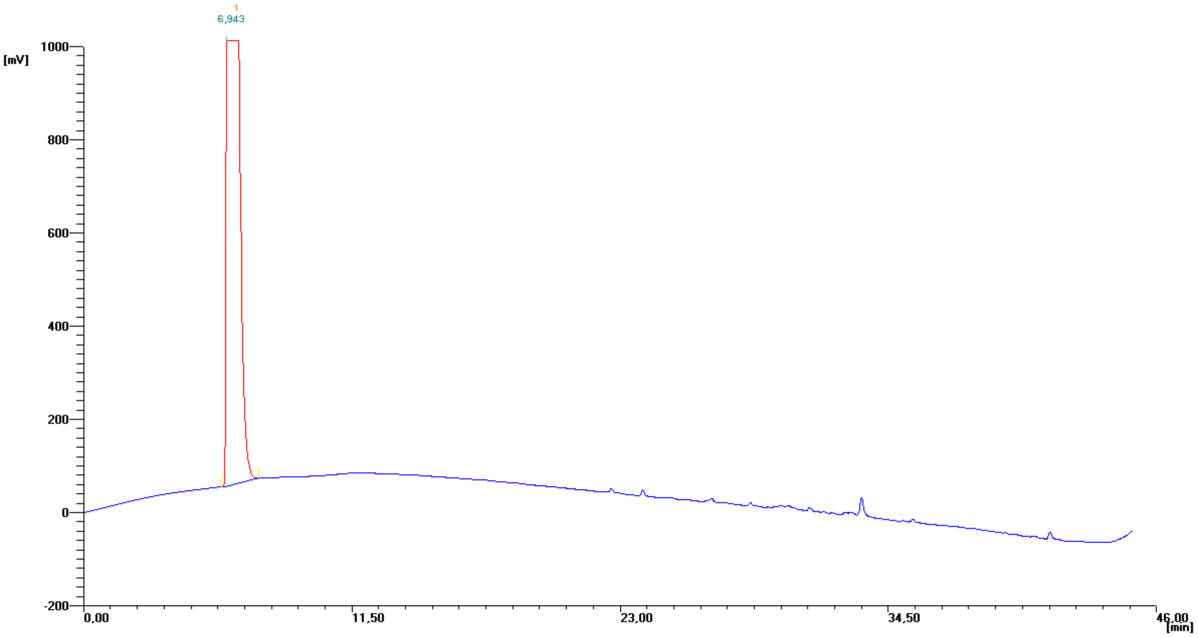

Fig. S65. HPLC spectrum of H-TyrTyrMet-OH (**15**).

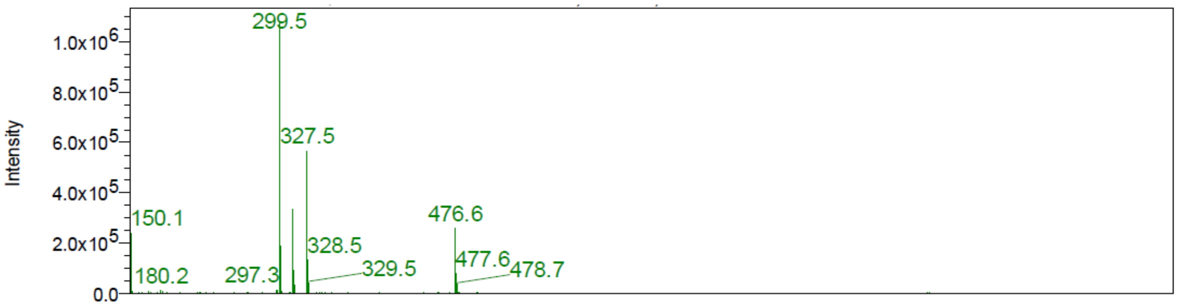

Fig. S66. MS spectrum of H-TyrTyrMet-OH (**15**).

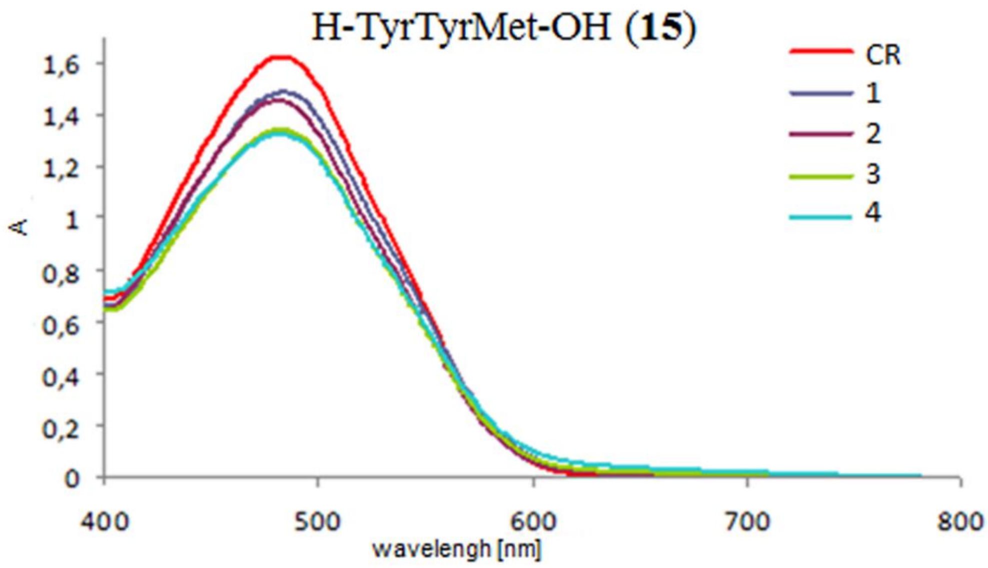

Fig. S67. UV-Vis spectra of H-TyrTyrMet-OH (**15**), incubation with CR.

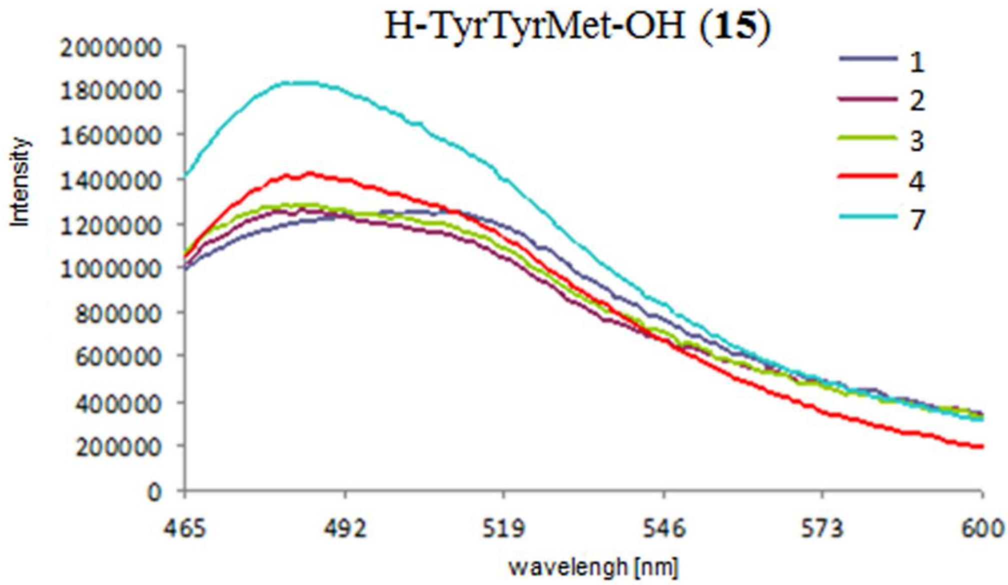

Fig. S68. Florescence spectra of H-TyrTyrMet-OH (**15**), incubation with ThT.

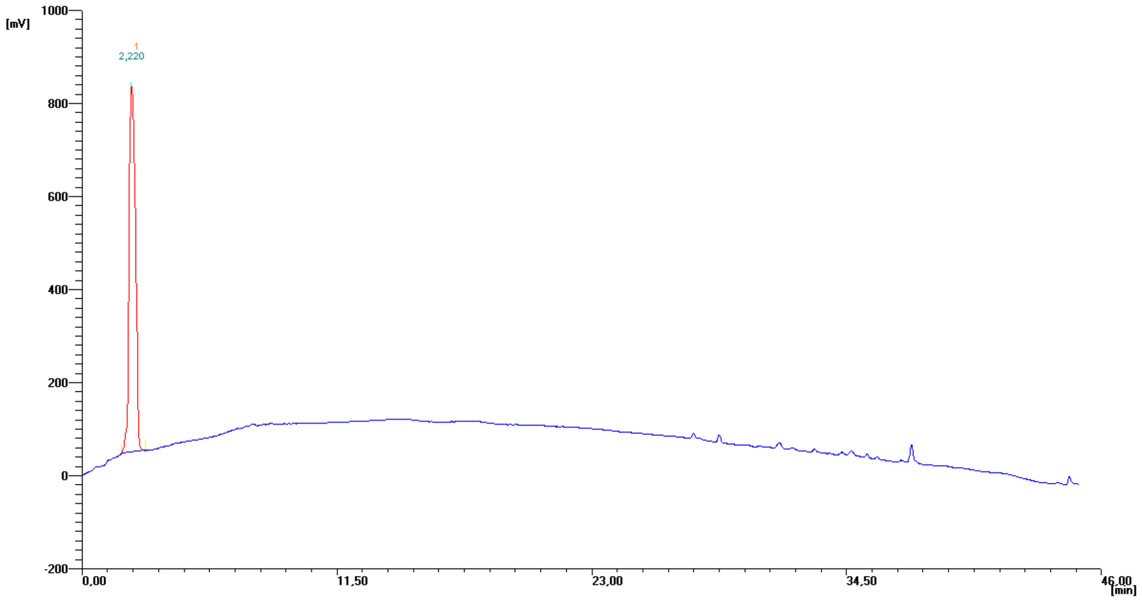

Fig. S69. HPLC of H-PheMetPhe-OH (**16**).

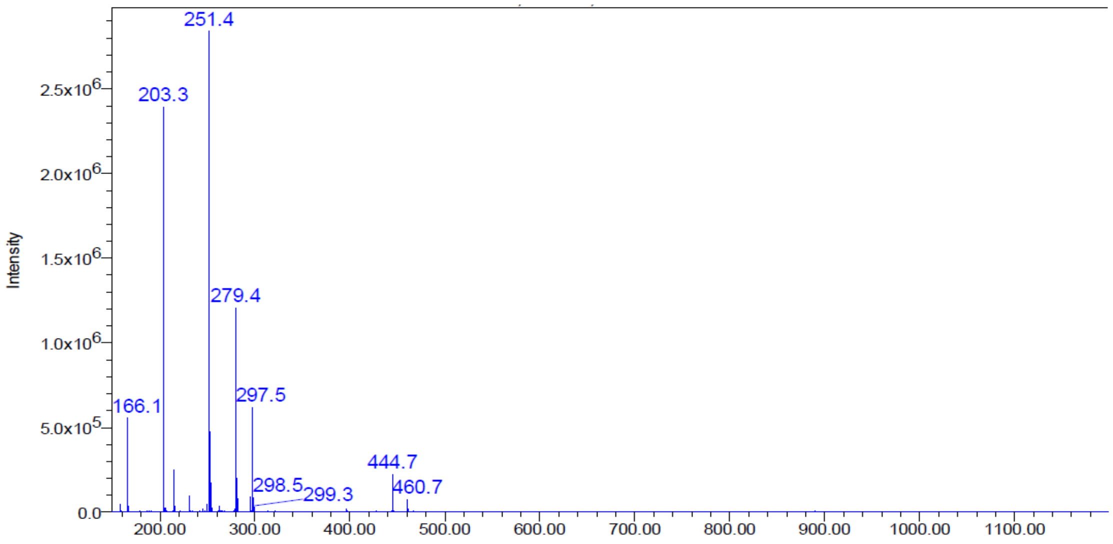

Fig. S70. MS spectrum of H-PheMetPhe-OH (**16**).

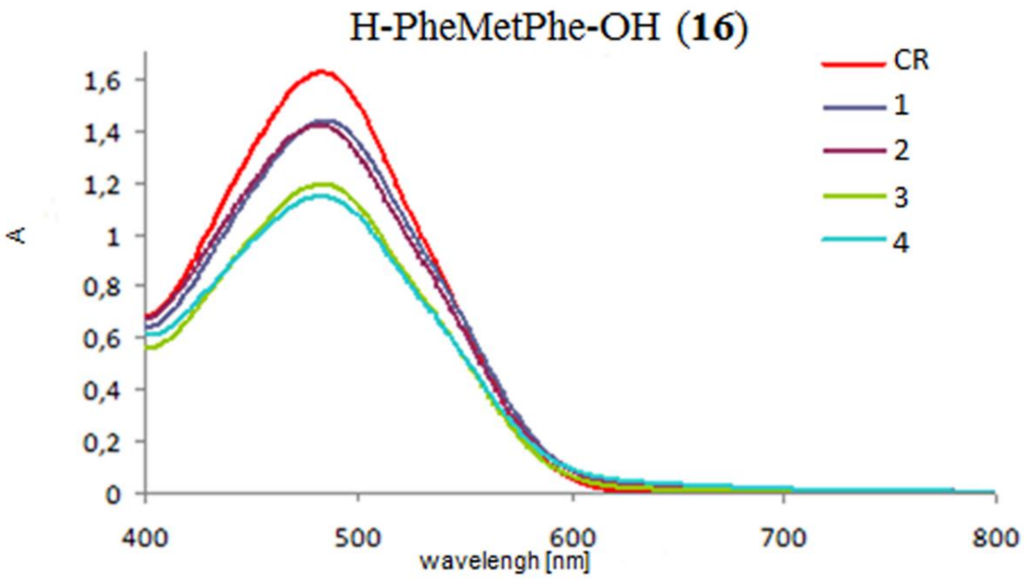

Fig. S71. UV-Vis spectra of H-PheMetPhe-OH (**16**), incubation with CR.

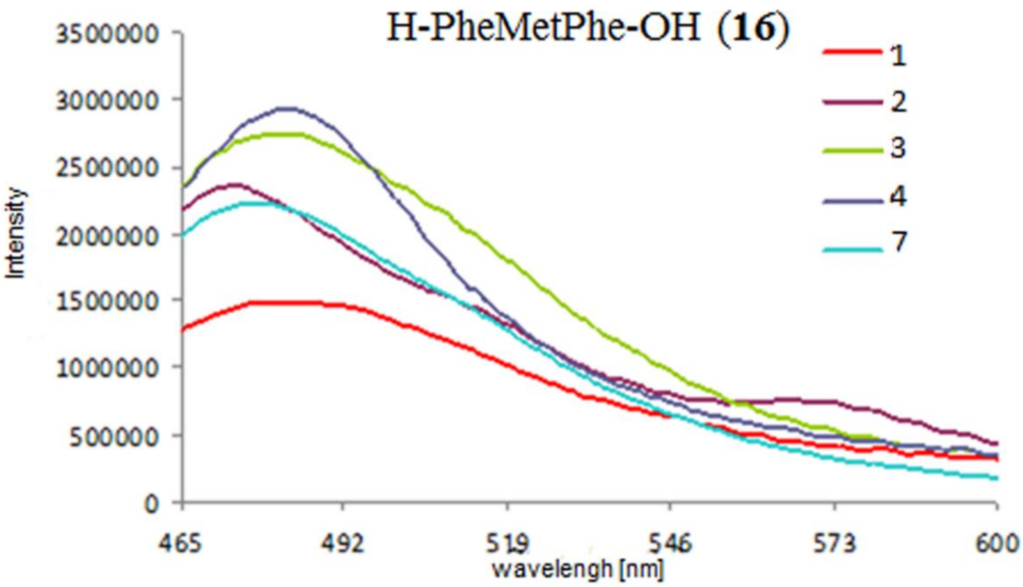

Fig. S72. Fluorescence spectra of H-PheMetPhe-OH (**16**), incubation with ThT.

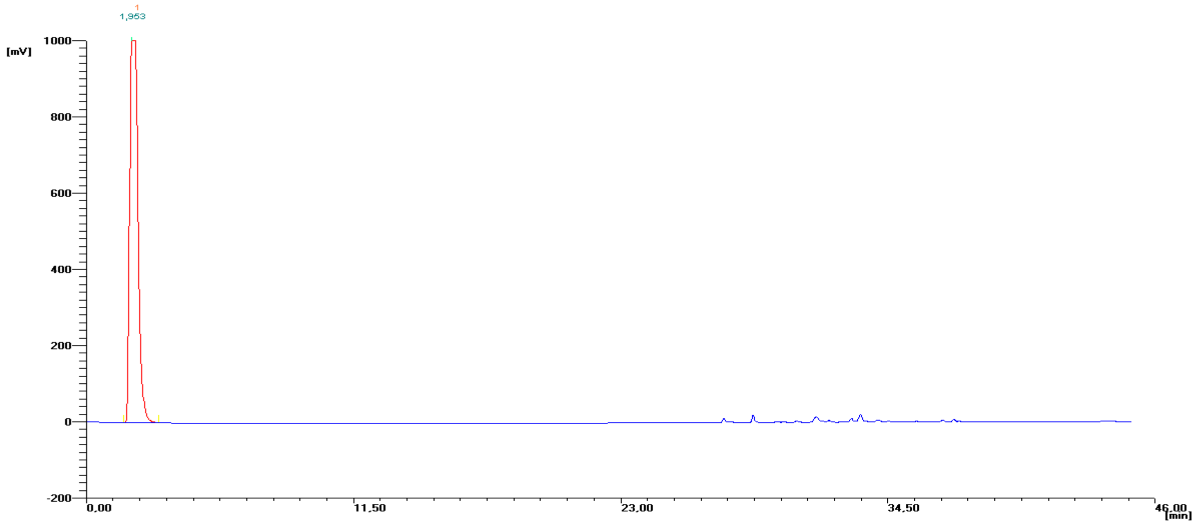

Fig. S73. HPLC of H-TrpMetTrp-OH (**17**).

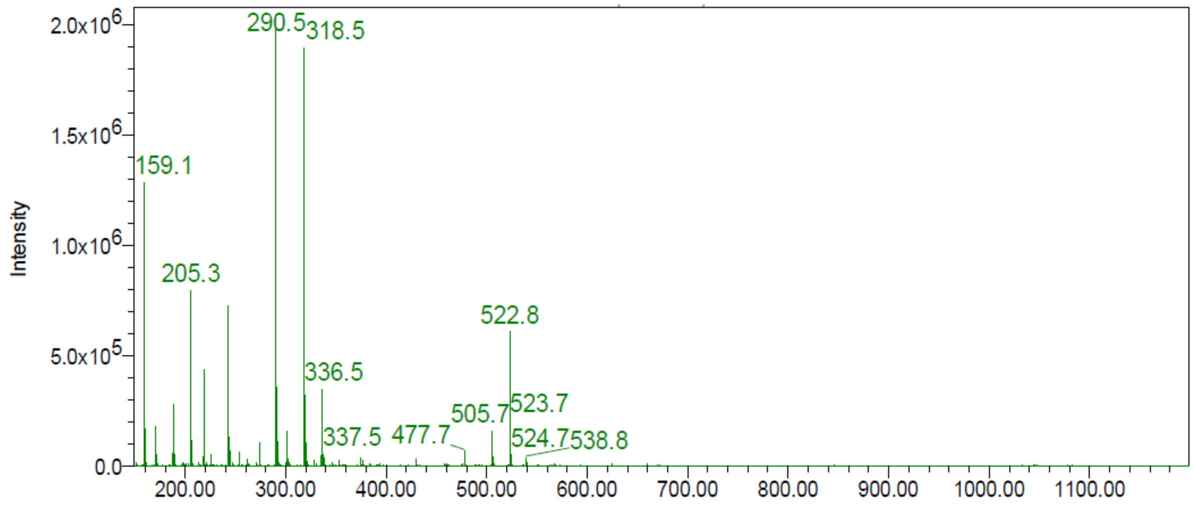

Fig. S74. MS spectrum of H-TrpMetTrp-OH (**17**).

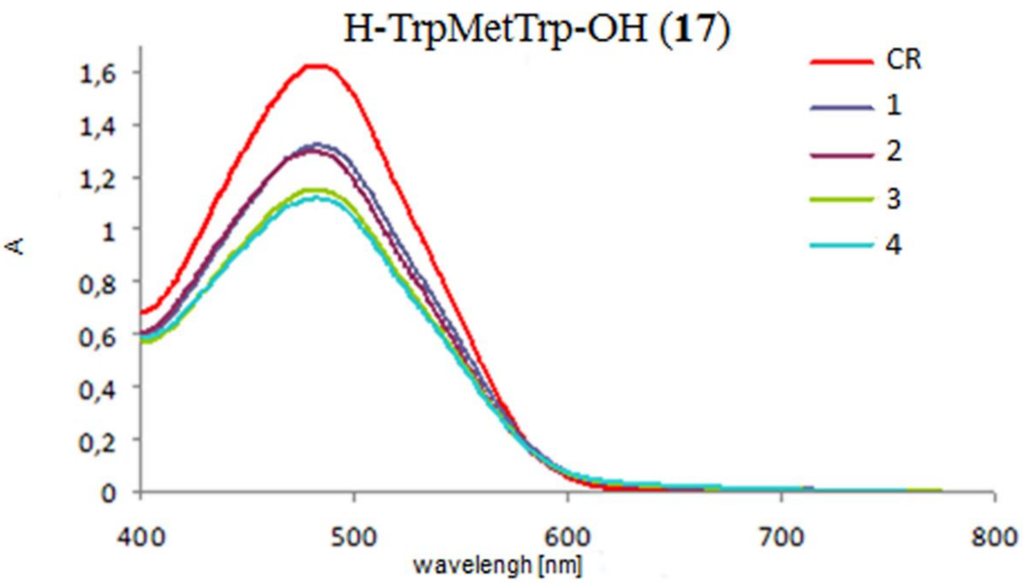

Fig. S75. UV-Vis spectra of H-TrpMetTrp-OH (**17**), incubation with CR.

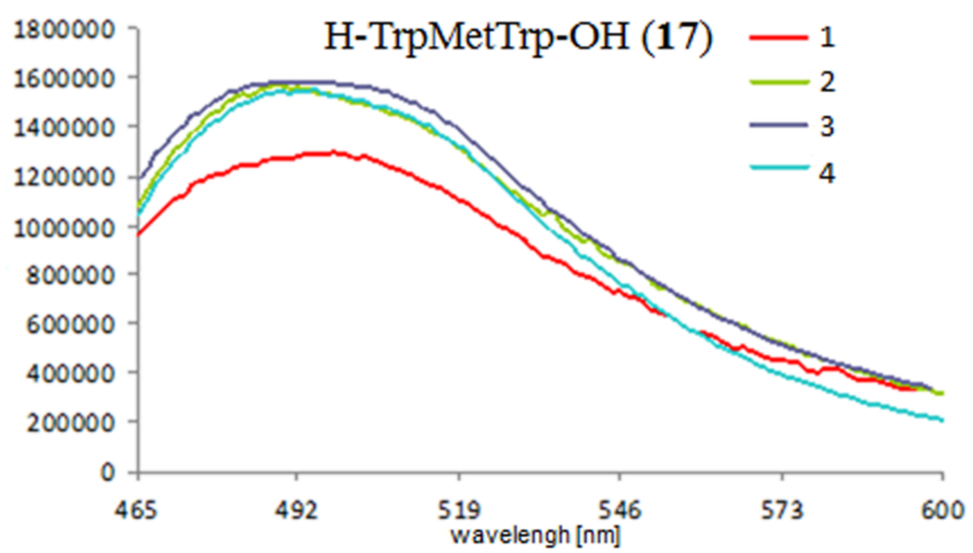

Fig. S76. Fluorescence spectra of H-TrpMetTrp-OH (**17**), incubation with ThT.

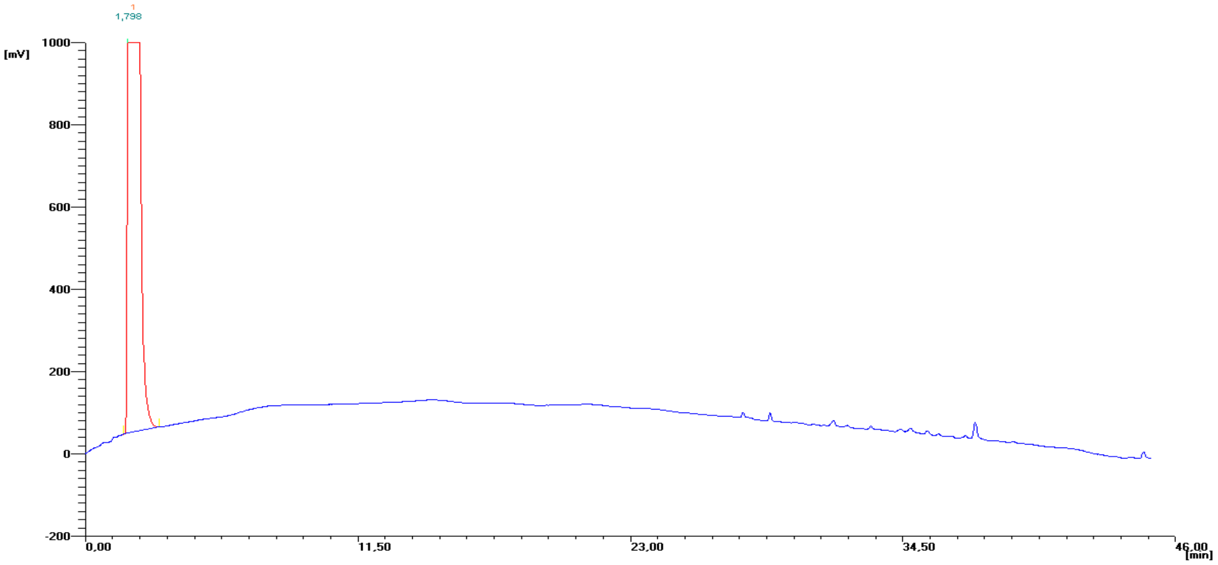

Fig. S77. HPLC of H-TyrMetTyr-OH (**18**).

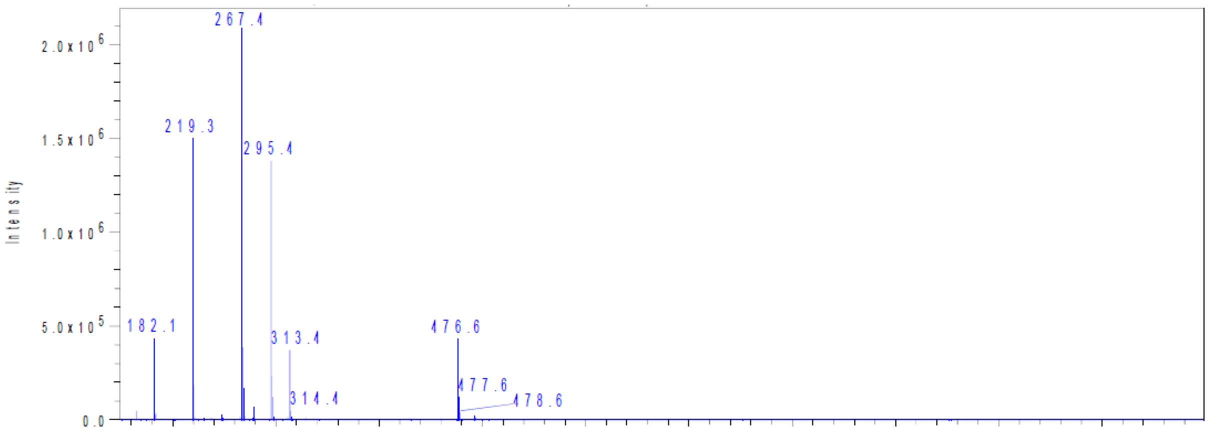

Fig. S78. MS spectrum of H-TyrMetTyr-OH (**18**).

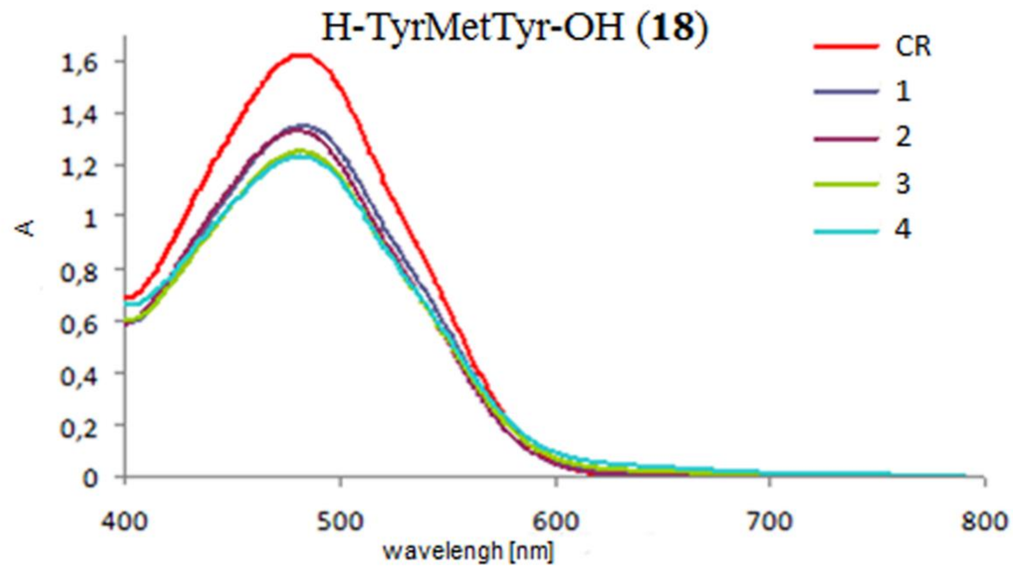

Fig. S79. UV-Vis spectra of H-TyrMetTyr-OH (**18**), incubation with CR.

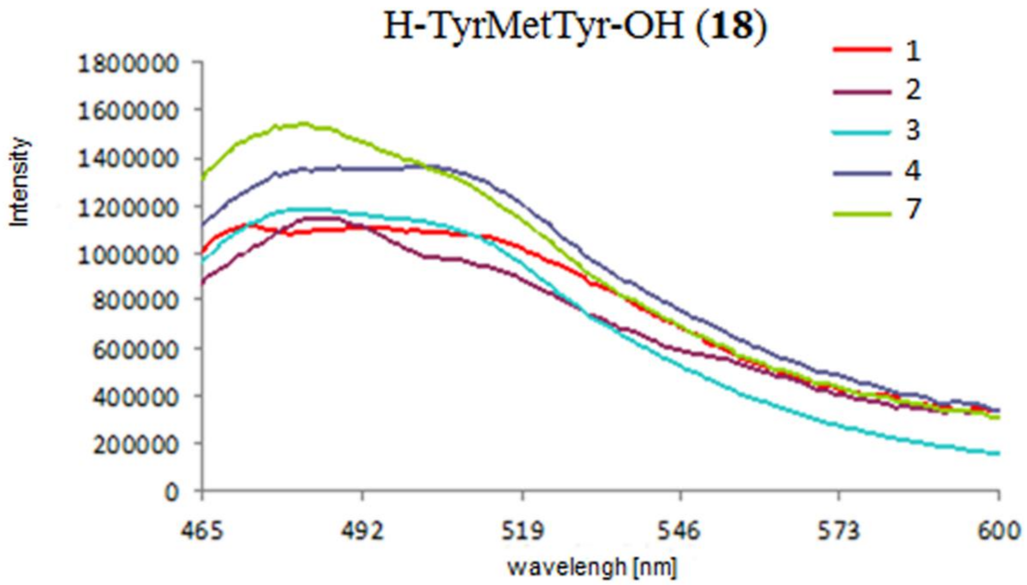

Fig. S80. Fluorescence spectra of H-Tyr-Met-Tyr-OH (**18**), incubation with ThT.

Table S1. Statistics on cytotoxicity studies, endothelial cell line EA.hy 926. Concentration = 0.025%.

		**LIVE**													**DEAD**													
		Ctr	**1-*ent***	**2**	**4-*ent***	**5**	**6**	**7-*ent***	**9**	**11**	**13**	**14**	**15**	**18**	Ctr	**1-*ent***	**2**	**4-*ent***	**5**	**6**	**7-*ent***	**9**	**11**	**13**	**14**	**15**	**18**
**LIVE**	Ctr	-																									
	**1-*ent***	***	-																								
	**2**	***	***	-																							
	**4-*ent***	***	***	***	-																						
	**5**	ns	***	**	ns	-																					
	**6**	ns	**	***	**	**	-																				
	**7-*ent***	***	***	***	ns	ns	***	-																			
	**9**	***	ns	ns	***	***	*	***	-																		
	**11**	ns	ns	**	***	***	***	***	ns	-																	
	**13**	***	ns	ns	***	***	**	**	ns	ns	-																
	**14**	***	***	ns	**	***	***	ns	***	ns	ns	-															
	**15**	ns	ns	**	*	**	***	ns	ns	***	**	***	-														
	**18**	*	**	*	***	**	***	ns	ns	ns	*	***	ns	-													
**DEAD**	Ctr	***	***	**	***	**	**	***	***	***	*	ns	***	***	-												
	**1-*ent***	***	***	*	***	**	*	***	***	***	*	ns	***	***	ns	-											
	**2**	***	***	*	***	**	*	***	***	***	*	ns	***	***	ns	ns	-										
	**4-*ent***	***	***	*	***	**	**	***	***	***	*	ns	***	***	ns	ns	ns	-									
	**5**	***	***	**	***	**	*	***	***	***	*	ns	***	***	ns	ns	ns	ns	-								
	**6**	***	***	*	***	**	*	***	***	***	*	ns	***	***	ns	ns	ns	ns	ns	-							
	**7-*ent***	***	***	*	***	**	*	***	***	***	*	ns	***	***	ns	ns	ns	ns	ns	ns	-						
	**9**	***	***	*	***	**	*	***	***	***	*	ns	***	***	ns	ns	ns	ns	ns	ns	ns	-					
	**11**	***	***	**	***	**	*	***	***	***	*	ns	***	***	ns	ns	ns	ns	ns	ns	ns	ns	-				
	**13**	***	***	*	***	**	**	***	***	***	*	ns	***	***	ns	ns	ns	ns	ns	ns	ns	ns	ns	-			
	**14**	***	***	*	***	**	**	***	***	***	*	ns	***	***	ns	ns	ns	ns	ns	ns	ns	ns	ns	ns	-		
	**15**	***	***	*	***	**	**	***	***	***	*	ns	***	***	ns	ns	ns	ns	ns	ns	ns	ns	ns	ns	ns	-	
	**18**	***	***	*	***	**	*	***	***	***	*	ns	***	***	ns	ns	ns	ns	ns	ns	ns	ns	ns	ns	ns	ns	-

*** $p < 0.001$, ** $p < 0.01$, * $p < 0.05$.

Table S2. Statistics on cytotoxicity studies, endothelial cell line EA.hy 926. Concentration = 0.05%.

		LIVE													DEAD												
		Ctr	**1-*ent***	**2**	**4-*ent***	**5**	**6**	**7-*ent***	**9**	**11**	**13**	**14**	**15**	**18**	Ctr	**1-*ent***	**2**	**4-*ent***	**5**	**6**	**7-*ent***	**9**	**11**	**13**	**14**	**15**	**18**
**LIVE**	Ctr	-																									
	**1-*ent***	***	-																								
	**2**	***	ns	-																							
	**4-*ent***	***	***	***	-																						
	**5**	***	**	***	ns	-																					
	**6**	***	**	***	ns	***	-																				
	**7-*ent***	***	***	ns	ns	***	***	-																			
	**9**	***	ns	ns	ns	***	*	***	-																		
	**11**	***	ns	ns	***	***	***	***	ns	-																	
	**13**	***	ns	ns	ns	***	**	**	ns	ns	-																
	**14**	***	***	ns	***	**	***	ns	***	ns	***	-															
	**15**	***	ns	**	**	ns	ns	ns	ns	ns	ns	**	-														
	**18**	***	**	**	***	**	***	ns	ns	**	**	***	ns	-													
**DEAD**	Ctr	***	***	**	***	**	*	***	***	***	**	ns	***	***	-												
	**1-*ent***	***	***	**	***	**	**	***	***	***	**	ns	***	***	ns	-											
	**2**	***	***	**	***	**	**	***	***	***	**	ns	***	***	ns	ns	-										
	**4-*ent***	***	***	**	***	**	**	***	***	***	**	ns	***	***	ns	ns	ns	-									
	**5**	***	***	**	***	**	*	***	***	***	**	ns	***	***	ns	ns	ns	ns	-								
	**6**	***	***	**	***	**	*	***	***	***	**	ns	***	***	ns	ns	ns	ns	ns	-							
	**7-*ent***	***	***	**	***	**	*	***	***	***	**	ns	***	***	ns	ns	ns	ns	ns	ns	-						
	**9**	***	***	**	***	**	*	***	***	***	**	ns	***	***	ns	ns	ns	ns	ns	ns	ns	-					
	**11**	***	***	**	***	**	*	***	***	***	**	ns	***	***	ns	ns	ns	ns	ns	ns	ns	ns	-				
	**13**	***	***	**	***	**	*	***	***	***	**	ns	***	***	ns	ns	ns	ns	ns	ns	ns	ns	ns	-			
	**14**	***	***	**	***	**	*	***	***	***	**	ns	***	***	ns	ns	ns	ns	ns	ns	ns	ns	ns	ns	-		
	**15**	***	***	**	***	**	*	***	***	***	**	ns	***	***	ns	ns	ns	ns	ns	ns	ns	ns	ns	ns	ns	-	
	**18**	***	***	**	***	**	*	***	***	***	**	ns	***	***	ns	ns	ns	ns	ns	ns	ns	ns	ns	ns	ns	ns	-

*** $p < 0.001$, ** $p < 0.01$, * $p < 0.05$.
